# Data driven discovery of cyber physical systems

**DOI:** 10.1038/s41467-019-12490-1

**Published:** 2019-10-25

**Authors:** Ye Yuan, Xiuchuan Tang, Wei Zhou, Wei Pan, Xiuting Li, Hai-Tao Zhang, Han Ding, Jorge Goncalves

**Affiliations:** 1 School of Artificial Intelligence and Automation, Key Laboratory of Image Processing and Intelligent Control, Huazhong University of Science and Technology, 430074 Wuhan, People’s Republic of China; 2 State Key Lab of Digital Manufacturing Equipment and Technology, Huazhong University of Science and Technology, 430074 Wuhan, People’s Republic of China; 3 School of Mechanical Science and Engineering, Huazhong University of Science and Technology, 430074 Wuhan, People’s Republic of China; 4 Department of Cognitive Robotics, Delft University of Technology, Delft, Netherlands; 5 Department of Plant Sciences, University of Cambridge, Cambridge, CB2 3EA UK; 6 Luxembourg Centre for Systems Biomedicine, University of Luxembourg, 6, Avenue du Swing, L4367 Belvaux, Luxembourg

**Keywords:** Computational science, Nonlinear phenomena

## Abstract

Cyber-physical systems embed software into the physical world. They appear in a wide range of applications such as smart grids, robotics, and intelligent manufacturing. Cyber-physical systems have proved resistant to modeling due to their intrinsic complexity arising from the combination of physical and cyber components and the interaction between them. This study proposes a general framework for discovering cyber-physical systems directly from data. The framework involves the identification of physical systems as well as the inference of transition logics. It has been applied successfully to a number of real-world examples. The novel framework seeks to understand the underlying mechanism of cyber-physical systems as well as make predictions concerning their state trajectories based on the discovered models. Such information has been proven essential for the assessment of the performance of cyber-physical systems; it can potentially help debug in the implementation procedure and guide the redesign to achieve the required performance.

## Abstract (web summary)

Discovery of hybrid dynamical models for real-world cyber-physical systems remains a challenge. This paper proposes a general framework for automating mechanistic modeling of hybrid dynamical systems from observed data with low computational complexity and noise resilience.

## Introduction

Since the invention of computers, software has quickly become ubiquitous in our daily lives. Software controls domestic machines, such as washing and cooking appliances, aerial vehicles such as quadrotors, the scheduling of power generation and the monitoring of human body vital signals. These technologies embed cyber components throughout our physical world, in fact, almost all modern engineering systems involve the integration of cyber and physical systems. The integration of cyber and physical components provides new opportunities and challenges. On one hand, this integration produces new functionality in traditional physical systems, such as brakes and engines in vehicles, intelligent control systems for biochemical processes and wearable devices^1–3^. On the other hand, the integration of cyber components adds new layers of complexity, potentially seriously complicating their design to guarantee the performance. Cyber–physical systems (CPSs), such as modern power grids or autonomous cars, require to be economically and safely integrated into society. In power grids, the failure of transformer taps, capacitors and switching operations alter the dynamics of the grid, which can be extremely costly. We have, after all, already witnessed a massive power outage in Southern California on September 2011 due to a cascading failure from a single line tripping (which was not detected by operators using their model), costing billions of US dollars. In autonomous driving, autonomous cars are expected to be well-operated in multiple complex scenarios from driving on multi-lane highway to turning at intersections while obeying rules. These objectives are achieved through decisions made by high-level software and control by low-level computer systems, realizing the command using a combination of GPS/IMU, camera, radar and LIDAR data^4^. In such complex scenarios, guaranteeing CPS’s performance poses a fundamental challenge.

For performance guarantees, we require reliable models that capture essential dynamics. The central question this study seeks to answer, therefore, is how to reliably and efficiently automate mechanistic modeling of CPSs from data^5^. An appropriate mathematical model of CPS should recognize the hybridity of CPS, which comprises of discrete and continuous components due to the integration of software and physical systems, respectively. Hybrid dynamical systems (detailed below and Supplementary Note 2) use finite-state machines to model the cyber components and dynamical systems for the physical counterparts. Hybrid dynamical models can produce accurate predictions and enable assessments of the CPSs’ performance^6^. This paper presents a new method, namely identification of hybrid dynamical systems (IHYDE), for automating the mechanistic modeling of hybrid dynamical systems from observed data. IHYDE has low computational complexity and is robust to noise, enabling its application to real-world CPS problems.

There are various methods for identifying non-hybrid dynamical systems. Schmidt and Lipson^7^ propose a data-driven approach to determine the underlying structure and parameters of time-invariant nonlinear dynamical systems. Schmidt and Lipson’s method uses symbolic regression to identify the system, balancing model complexity and accuracy. However, symbolic regression has its possible limitations: it can be computationally expensive, does not scale to real large-scale systems, and is prone to overfitting. The work in refs. ^8–10^ expands the vector field or map of the underlying system into suitable function series; then, they use compressive sensing and sparse Bayesian learning techniques to accurately estimate various terms in the expansion. Later, Brunton et al.^11^ apply a sequentially thresholded least-square method to discover ordinary differential equations. Although these recent advances^8–11^ manage to reduce the expensive computational burden using compressive sensing and sparse learning, these methods cannot be applied to hybrid dynamical systems because of the complexity and switching behaviors in hybrid dynamical systems; basically, these algorithms cannot account for an unknown number of unknown subsystems that interact via unknown transition logics.

There have been a number of interesting results in hybrid dynamical system identification over the past two decades^12–23^. Ref. ^13^ gives a comprehensive literature review, summarizing major advances up to 2007. Methods span across several fields, such as algebraic geometry^14^, mixed integer programming^17^, bounded-error^18^, Bayesian learning^19^, clustering-based strategies^20^ and multi-modal symbolic regression^21^. The algebraic geometry^14^ and bounded-error^18^ methods can handle cases with unknown model order and number of subsystem models. However, algebraic–geometric methods cannot deal with discontinuous dynamics. The Bayesian approach in ref. ^19^ exploits available prior knowledge about modes and parameters of hybrid systems, which helps tuning parameters. Clustering-based methods^20^ are suitable for cases with little prior knowledge on physical systems. However, it requires prior knowledge of model order and number of subsystems. Despite clear merits of all these pioneering contributions, most methods focus on the simplest hybrid dynamical models: piecewise affine systems with linear transition rules^18^.

Recent pioneering results in refs. ^15,16^ use compressive sensing^24^ to identify the minimum number of subsystems by recovering a sparse vector-valued sequence from data. These algorithms tradeoff the mismatch between data and model predictions, and the energy of the noise. Breschi et al.^25^ proposes a regression approach based on recursive clustering and multi-class linear separation methods. To solve time-varying parameter identification on stochastic autoregressive models with exogenous inputs (ARX), ref. ^26^ employs expectation maximization algorithms to recursively solve mixed-integer optimizations problems. The work in ref. ^27^ proposes a method based on difference of convex functions programming to optimize non-smooth and nonconvex objective functions. Finally ref. ^28^ aims to identify switched affine models in a set membership framework, ref. ^29^ uses hybrid stable spline algorithms, where Gaussian processes model the impulse response of each subsystem, and ref. ^30^ uses symbolic regression.

IHYDE aims to provide a more flexible and general framework by directly discovering the number of subsystems, their dynamics and the transition logics from data. IHYDE deals with this problem in two parts: first, the algorithm discovers how many subsystems interact with each other and identifies a model for each one; second, the algorithm infers the transition logic between each pair of subsystems. Methods in refs. ^9,11,15^ can be viewed as special cases of the first step in this new framework. IHYDE is illustrated on a number of examples, ranging from power engineering and autonomous driving to medical applications, to demonstrate the algorithm’s application to various types of datasets.

## Results

### The IHYDE algorithm

This section is divided in two major parts. The first presents the proposed inference-based IHYDE algorithm using a simple example—a thermostat, while the second illustrates its applicability to a wide range of systems, from real physical systems to challenging in silico systems, and from linear to nonlinear dynamics and transition rules. Details of both the algorithm and how data was acquired or generated can be found in Supplementary Information.

**Fig. 1:**
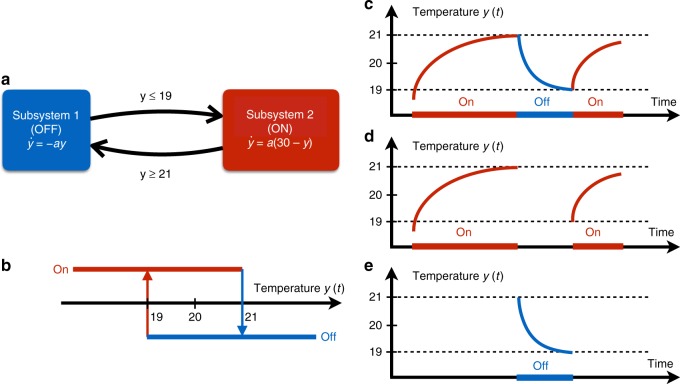
An illustrative toy example on a thermostat. **a** The physical dynamic equations plus the transition rules of the hybrid dynamical system. A transition rule is to turn the heater on when the temperature falls below 19°C, and switch it off when it reaches 21°C. When the heater is off, the temperature *y* dissipates to the exterior at a rate of **−***ay*(*t*) °C per hour, where *a* > 0 is related to the insulation of the room. When the heater is on, it provides a temperature increase rate of 30*a* °C per hour. **b** Visualization of transition rules of the relay hysteresis based on the temperature of the room. **c** A simulation of the temperature of the thermostat system. Red (blue) is associated with the heater on (off). **d**, **e** Separated time series of the temperature corresponding to the heater on (off) from the original temperature data

*The inference-based IHYDE algorithm applied to a thermostat:* This section explains the key concepts of IHYDE using one of the simplest and ubiquitous hybrid dynamical systems: a room temperature control system consisting of a heater and a thermostat. The objective of the thermostat is to keep the room temperature *y*(*t*) near a user specified temperature. At any given time, the thermostat can turn the heater on or off. When the heater is off, the temperature dissipates to the exterior at a rate of **−***ay*(*t*) °C per hour, where *a* > 0 is related to the insulation of the room. When the heater is on, it provides a temperature increase rate of **30***a* °C per hour (Fig. 1a).

Assume a desired temperature is set to 20 °C. Thermostats are equipped with hysteresis to avoid chattering, i.e., fast switching between on and off. A possible transition rule is to turn the heater on when the temperature falls below 19 °C, and switching it off when it reaches 21 °C (Fig. 1b). The goal of IHYDE is to infer both subsystems plus the transition logics from only the observed time-series data of the temperature (Fig. 1c). Next, we shall illustrate the key ideas of the proposed algorithm on this simple example.

*Inferring subsystems:* The first step of the proposed IHYDE algorithm is to iteratively discover which subsystem of the thermostat generated which time-series data. Initially, the algorithm searches for the subsystem that captures the most data, since this subsystem would explain the largest amount of data. In this case, the algorithm would firstly discover subsystem 2 (heater on) since more than half of the data correspond to that subsystem (see Fig. 1c). The time-series portion of the data (Fig. 1d) is then used to find the dynamics of subsystem 2. The algorithm is then repeated on the remaining data (Fig. 1e). In this case, there is only one subsystem left (heater off). Hence, the algorithm classifies all the rest data to a subsystem and identifies the corresponding dynamics.

*Inferring transition logics:* The second and final step is to identify the transition logics between the two subsystems, i.e., what triggered the transitions from on to off and from off to on. Starting with subsystem 2 (heater on) and its associated data in Fig. 1d, the algorithm first learns that no switch occurs when the temperature changes from just below 19 °C to near 21 °C. Since the switch happens when the temperature reaches 21 °C, the algorithm concludes that the switch from on to off happens when *y*(*t*) = 21. In practice, however, the software detects the switches when *y*(*t*) ≥ 21. Similarly, from Fig. 1e, the algorithm learns that the switch from on to off happens when *y*(*t*) ≤19.

In summary, IHYDE automatically learns the dynamics of all subsystems and the transition rules from one subsystem to another. While this is a simple system, as we will show next, this is true even in the presence of a large number of subsystems, potentially with nonlinear dynamics and transition rules.

### Applications

Next, we illustrate how IHYDE can be applied to a wide range of applications, from power engineering to robotics and medicine, showing the flexibility, applicability and power of IHYDE to model complex CPSs. Here, we consider the following examples. (1) Benchmark examples (see Supplementary Note 3, Examples 1, 2, 3 and 4); (2) Autonomous vehicles and robots: design and validation of an autonomous vehicle (see Supplementary Note 3, Example 5); (3) Complex electronics: Chua’a circuit (see Supplementary Note 3, Example 6); (4) Monitoring of industrial processes: monitoring a wind turbine (see Supplementary Note 3, Example 7); (5) Power systems: transmission lines and smart grids (see Supplementary Note 3, Examples 8 and 9); (6) Medical applications: heart atrial active potential monitoring (see Supplementary Note 3, Example 10). To test IHYDE’s performance, these systems will include both experimental and synthetic datasets. Details can be found below and in Supplementary Note 3.

**Fig. 2:**
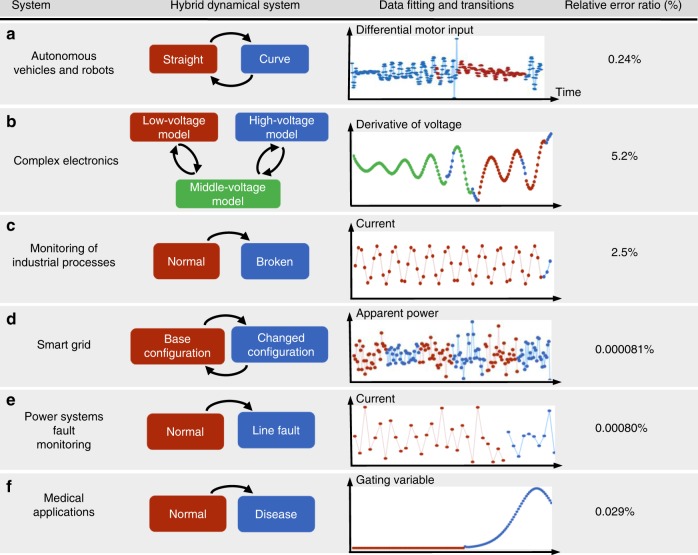
Summary of IHYDE algorithm applied to numerous examples. IHYDE has been applied to six examples in different applications. The first column illustrates the systems, while the second column shows the different subsystems plus the transition rules. Each subsystem is associated with a particular color. The third column shows the original time-series data (dots) in the color associated with the subsystem that generated it, the fitted data from the identified models (lines connecting the dots), and the location of the transitions (changes in colors). The last column presents the relative error ratio^31^ between the true data and the data simulated by the fitted model. A small error ratio indicates a good agreement between the true and discovered systems, and serves as a measure of the performance of IHYDE

Figure 2 contains a summary of the most important systems analyzed in the paper. The first three examples are based on real experimental data, while the other three are based on simulated data. The first two columns illustrate the systems and the corresponding subsystems, respectively, where each subsystem is associated with a particular color. The third column shows the original time-series data (dots) in the color associated with the subsystem that generated it, the fitted data from the identified models (lines connecting the dots), and the location of the transitions (changes in colors). Note that, at this resolution, the original data and the data obtained from the fitted models are indistinguishable. The last column presents the relative error ratio^31^ between the true data and the data simulated by the fitted model. A small error ratio indicates a good agreement between the true and modeled systems, and serves as a measure of the performance of IHYDE. Data are either collected (real systems) or simulated (synthetic systems) and capture all key transitions. As seen in column 3 and column 4 of Fig. 2, IHYDE successfully discovers the original dynamics that generated the data in all examples with extremely high precision (nearly zero identification errors). First, it is able to classify each time point according to the respective subsystem that generated it. Second, it identifies the dynamics of each subsystem with a very small error (<0.03% on all simulated examples). Finally, it correctly identifies the transition rules between subsystems.

*Autonomous vehicles and robots—design and validation:* To demonstrate IHYDE’s usefulness in designing and validating complex systems, we test the algorithm on an autonomous vehicle, custom built in the lab (Fig. 2a). Typically, the design process of complex systems consists of an arduous, time-consuming and trial-and-error based approach: start from an initial design, evaluate its performance and revise it until the performance is satisfied. A primary issue with this iterative approach is that when a design fails to meet desired specifications, many times engineers have little to no insight on how to improve the next iteration. Often, an engineer cannot discern whether the issue is due to poor mechanical design, issues with the software or factors that were not considered. And this is also true with other general complex CPSs that involve interactions between physical/mechanical parts and software.

The autonomous electrical car consists of a body, a MK60t board, a servo motor, a driving motor and a camera. The design goal of the autonomous car was to successfully run through a winding track as quickly as possible. Using an embedded camera, the software captures information of the upcoming road layout to ascertain whether a straightway or a curve is coming up. Based on this information, the motor chooses an appropriate power to match the desired speed control strategy. For the purpose of illustration, we consider a simple controller that provides higher velocities on straightways and lower velocities on curves. In addition, simple feedback controllers help the car follow the chosen speed and stay on the track. The speed-control strategy is based on incremental proportional and integral (PI) control that keeps the car at the correct speed, while the switching rule decides on the correct speed depending on whether a straight or curve is coming up.

For the first-round design, we deliberately swap the straightway and curve speeds to mimic a software bug. As a consequence, the car moves rather slow in the straights and leaves the tracks in the curves (Supplementary Movie 1). While in this case it is rather easy for engineers to spot the software bug; debugging, in general, can be extremely difficult, sometimes only possible by trial and error, and, as a consequence, very time consuming. One would like to check whether these types of bugs could be detected by IHYDE. Indeed, from the data generated by the faulty system, IHYDE compares its discovered models with the to-be-built ones, pinpoints the incorrect speed controllers. Hence, from data alone, IHYDE has successfully discovered both the control strategy and software of the designed car and pinpointed the software bug.

*Complex electronics—Chua's circuit*: Debugging and verifying complex, large-scale electronics can be a daunting experience. Modeling could help identify whether a device has been built according to the desired specifications by identifying faulty connections or incorrect implementations. Simple electrical circuits, such as RLC circuits, are linear and easy to model. However, most electronic circuits introduce both nonlinear dynamics and switches (e.g., diodes and transistors), which can lead to extremely complex behaviors. Thus, modeling such systems can be very challenging.

To illustrate IHYDE’s applicability in this scenario, we build an electronic circuit that exhibits complex behaviors. We choose a well-known system, called the Chua’s circuit^32^, that exhibits chaotic trajectories (Fig. 2b). Chaotic systems constitute a class of systems that depend highly on initial conditions, and makes simulation and modeling very challenging. Our circuit consists of an inductor, two capacitors, a passive resistor and an active nonlinear resistor, which fits the condition for chaos with the least components. The most important active nonlinear resistor is a conceptual component that can be built with operational amplifiers and linear resistors. The resulting nonlinear resistor is piecewise linear, making the Chua’s circuit a hybrid dynamical system with a total of three subsystems and a well-defined transition logic.

After collecting real data measured from the circuit, IHYDE successfully captures the dynamics of system and the transition rules between identified subsystems. In particular, the nonlinear dynamics are consistent with the true parameters of the circuit elements. As with all examples, modeling of the Chua's circuit is achieved using only the data and the basic knowledge of the field (to guide the choice of nonlinearities), without other assumptions on dynamics or switching behaviors. This application demonstrates the capabilities of IHYDE in revealing underlying mechanistic model of complex circuit.

*Monitoring of industrial processes—wind turbines platform*: Next, we consider the problem of real-time monitoring industrial processes. Modeling large-scale industrial processes is challenging due to the large number of parts involved, nonlinear dynamics and switching behaviors. Switches, in particular, are caused by breaking down of parts (due to wear and tear) and turning processes on and off, which introduce discontinuities in the dynamics. We propose IHYDE as a tool to detect these switches as quickly as possible to prevent lengthy and expensive downtimes in industrial processes.

To put IHYDE to the test, we use real data from a wind turbine platform built in ref. ^33^. The data consist of measurements of the current generated by the wind turbine experimental platform under different operating conditions (Fig. 2c). The system included a 380-V power supply, a variable load, a power generator, a motor, a fan, two couplings and a gearbox that transmits the energy generated by the wind wheel to the power generator^33^. We perform experiments under normal and faulty conditions (a broken tooth of gearbox) and down-sample the measuring current of the wind turbine with a period of 0.3 s. In both experiments, the generator speed is 200 revolutions per minute and the load is 1.5 KNm.

IHYDE is tested under two different scenarios: offline modeling, used, for example, at the design stage; and online modeling, for real-time monitoring. In offline modeling, all the data are available for modeling, while in real-time monitoring only past data are available, and the system is continuously modeled as new data are gathered. In offline modeling, IHYDE identifies two linear subsystems, corresponding to the system in the two different conditions. In addition, it correctly detects the fault right after it happens and infers the transition logic. In online modeling, a model predicts the next time-series data point, and compares it with the real one, when this becomes available. If the difference is high, IHYDE detects a transition, builds a new model, and compares it with the old model to pinpoint the location of the fault. This example focuses on online modeling: the fault is detected within only three data points following its occurrence. This application demonstrates the capabilities of IHYDE in online monitoring of industrial processes.

*Power systems—smart grids and transmission lines*: Smart grids have been gaining considerable attention in the last decades and are transforming how power systems are developed, implemented and operated. They considerably improve efficiency, performance and makes renewable power feasible. In addition, it overhauls aging equipment and facilitates real-time troubleshooting, which decreases brownouts, blackouts and surges. As with all critical infrastructures, smart grids require strict safety and reliability constraints. Thus, it is of great importance to design monitoring schemes to diagnose anomalies caused by unpredicted or sudden faults^34^. Here, we consider two examples of power systems: real-time modeling to control smart grids and pinpointing the location of a transmission line failure.

We start by illustrating how IHYDE can model and control smart grids in real-time. Accurate model information is not only necessary for daily operation and scheduling, but also critical for other advanced techniques such as state estimation and optimal power flow computation. However, such information is not always available in distribution systems due to frequent model changes^35^. These changes include: high uncertainty in distributed energy resources, such as components being added and removed from the network; unexpected events, such as line faults and unreported line maintenance; and trigger of automatic control and protection measures. We apply IHYDE to identify network models and infer transition logics, capturing model changes from advanced metering infrastructure data and in real-time. The 33-bus benchmark distribution system^36^ generates the data. It is a hypothetical 12.66 KV system with a substation, 4 feeders, 32 buses and 5 tie switches^36^. The system is not well-compensated and lossy, and is widely used to study network reconfiguration problems. Assume the loads on some remote nodes of a feeder suddenly increase, causing voltage sag. Subsequently, an operator takes switch action for load balancing and voltage regulation. Supplementary Fig. 12 depicts the switching topologies and the real transition logic. Suppose we can measure all active and reactive power consumptions, and voltage phasors of the nodes. Hence, the system is changing between two configurations corresponding to topologies when some switches turn on and off. For each node and subsystem, IHYDE successfully identifies the responding column of the admittance matrices with nearly zero identification errors. The identified admittance matrices at the switching time instants are very different from that of the previous moments, indicating a model switching (corresponding to changes in colors on the data in Fig. 2d). Indeed, the identified logic is consistent with the real logic and demonstrates that IHYDE can reveal voltage drops at specific nodes in real-time and suggest switch action to avoid sharp voltage drops.

To simulate a transmission line failure, we assume that a transmission line fails between two buses in the network. We will use a standard benchmark IEEE 14-bus power network^37^. This system consists of generators, transmission lines, transformers, loads and capacitor banks. Looking directly at the generated data (Fig. 2e), it is not clear when the fault occurred, and much less what happened at the time of failure and where it was located. This is because the power system compensated the failure by rerouting power across other lines. IHYDE, however, can immediately detect the occurrence of this event and determine its location. This is done by estimating the new admittance matrix using only ten measurements following the failure (corresponding to 166.7 ms, according to the IEEE synchrophasor measurements standard C37.118, 2011). Basically, it successfully discovers both subsystems (normal and failure) from data and calculates the difference of the discovered subsystems (leading to the location of the fault). Given the frequency at which Phasor Measurement Units (PMUs) sample voltage and current, IHYDE is able to locate the fault in a few hundred milliseconds after the event occurs, enabling the operators to detect the event, identify its location and take remedial actions in real-time.

*Medical applications—heart atrial active potential monitoring:* The development of medical devices is another active research area. Especially, with the widespread use of wearables and smart devices, there is an exponential growth of data collection. These data require personalized modeling algorithms to extract critical information for diagnosis and treatments. Within this context, we apply IHYDE to model data gathered from a human atrial action potential (AP) system^38^. The human atrial AP and ionic currents that underlie its morphology are of great importance to our understanding and prediction of the electrical properties of atrial tissues under normal and pathological conditions.

The model captures the spiking of the atrial AP. In particular, two gating variables capture the fast and slow inactivation with switching dynamics. Following a spike, these two variables raise, preventing a new spike. Eventually, as the AP returns to low values, the inactivation dynamics switch back, and in time allow a new spike to take place. The goal is to test whether IHYDE can detect these transitions, together with the rules that led to the switch. Two scenarios are considered here: the first scenario assumes the first-principle model parameterization is available while the second not. In the first scenario, IHYDE indeed identifies the two subsystems, together with their dynamics, and pinpoints the changing logic correctly (Fig. 2f). For the second scenario, we repeat the modeling of this system, this time assuming that the choice of dictionary functions is unclear and/or the domain knowledge is lacking. In such cases, we consider a canonical dictionary function, such as polynomials approximations. IHYDE can still detect the transition points. However, the nonlinear dynamics are different than the true ones: as expected, it identifies instead a polynomial approximation of the original nonlinear dynamics. While these dynamics can still be used for simulation and trajectory prediction, they are not in a form that reveals physical meaning. For an interpretable model, we require domain knowledge. Hence, IHYDE provides a reliable model to study the system and to build devices to detect abnormal AP.

## Discussion

This work presents a new framework for identifying CPSs from data. Current state-of-the-art methods assume either parameterization of the system and/or the exact dynamics of subsystems, number of subsystems, or the switching rules. Instead, IHYDE only requires similar assumptions to those in literature. For example, full state measurements, linear dependence of the to-be-identified parameters and the choice of dictionary functions generally guided by the area of the application^9,11^. IHYDE successfully identifies complex mechanistic models directly from data, including the subsystem dynamics and their associated transition logics. The proposed method differs from classical machine learning tools, such as deep neural network models^39^, which typically do not provide insight on the underlying mechanisms of the systems (as the state-variables and learned parameters have no direct meaning). While IHYDE is inspired by prior work in symbolic regression^30^, it has much lower computational complexity due to the use of convex optimization formulation. As a result, it can solve large-scale CPSs, facilitating its application to complex real-world problems.

After IHYDE models a CPS, the resulting model can help verify the design specifications and predict future trajectories. If the CPS model reveals bugs or flaws in the implementation, it can potentially guide the redesign to achieve the required performance. Applications include robotics and automated vehicles, where data-driven models promise to reduce the reliance on trial and error. Furthermore, IHYDE can monitor, detect and pinpoint real-time faults of CPSs (for example, power systems), thereby helping avoid catastrophic failures. As seen in the results section, IHYDE can be applied to a wide range of applications. Supplementary Information includes additional examples on canonical hybrid dynamical systems^30^. As before, IHYDE successfully identifies both the subsystems and the transition rules (Supplementary Note 3, Examples 1–4).

One more thing, IHYDE unifies previous results as it can discover not only hybrid dynamical systems, but also non-hybrid dynamical systems (i.e., time-invariant linear and nonlinear systems^9,11^) as special cases. This is confirmed in (Supplementary Method 1), where IHYDE successfully identifies the original canonical dynamical systems from the data in ref. ^11^ (Supplementary Table 48). Hence, IHYDE provides a unified approach to the discovery of hybrid and non-hybrid dynamical systems.

While the approach has a number of advantages, there are still some open questions. First, it requires a new theory to understand when particular datasets are informative enough to uniquely identify a single (the true) hybrid dynamical system. Identifiability is a central topic in system identification and provides guarantees that there does not exist multiple systems that can produce the same data. This is illustrated in Supplementary Discussions, where we construct several hybrid dynamical systems that yield the exact same data, and hence cannot be differentiated from data alone. A second issue lies in the linear parameterization of the model. For equations whose parameters enter nonlinearly, gradient descent can be applied to obtain a local minimizer, although in this case a global optimum cannot typically be guaranteed. Finally, the choice of dictionary functions is generally guided by the area of the system. Any insight or domain knowledge to construct dictionary functions for hybrid dynamical systems can help reduce computational burden and improve model accuracy. When the domain knowledge in unclear or lacking, canonical dictionary functions, such as polynomials, kernels, Fourier series, can approximate the true dynamics. An example in Supplementary Discussion 3 illustrates how a polynomial series successfully approximates a sinusoid. However, in these cases, the exact original true function may be lost or hard to find, as illustrated in Supplementary Note 3, Example 10. Therefore, while IHYDE can still detect the location of switches, it discovers different dynamics based on the choice of the canonical dictionary function. Nevertheless, these dynamics can still be used for prediction since they still approximate the main dynamics of each subsystem (see for example, Supplementary Discussion 3).

## Methods

### The theoretical foundation of IHYDE algorithm

Motivated by the above example, we shall give a formal definition of hybrid dynamical systems. Physical systems are characterized by inputs ${\mathbf{u}}(t) \in {\mathbb{R}}^m$ and outputs ${\mathbf{y}}(t) \in {\mathbb{R}}^n$. Based on these variables, at any given time a particular mode *m*(*t*) is chosen from a possible total of *K* modes, i.e., *m*(*t*) ∈ {1, 2, $\ldots, K$}. Each mode corresponds to a particular set of physical parameters. The physical system evolves according to sets of differential equations: $\frac{{{\mathrm{{d}}}{\mathbf{y}}(t)}}{{{\mathrm{{d}}}t}} = {\mathbf{F}}_k\left( {{\mathbf{y}}(t),{\mathbf{u}}(t)} \right),k = 1,2, \ldots ,K,$ where each **F**_*k*_(**y**(*t*), **u**(*t*)) is related to the dynamics of subsystem *k*. Assume **y**(*t*) and **u**(*t*) are sampled at a rate *h* > 0, i.e. sampled at times 0, *h*, 2*h*, 3$h, \ldots$. For fast enough sampling (for small sampling period *h*), one of the simplest method to approximate derivatives is to consider $\frac{{{\mathrm{{d}}}{\mathbf{y}}(t)}}{{{\mathrm{{d}}}t}} \approx \frac{{{\mathbf{y}}(t + h) - {\mathbf{y}}(t)}}{h},$ which yields the discrete-time system ${{\mathbf{y}}(t + h) = {\mathbf{y}}(t) + h\ {\mathbf{F}}_{k}({\mathbf{y}}(t),{\mathbf{u}}(t)) \ {\buildrel{\Delta}\over {=}}\ {\bf{f}}_k({\mathbf{y}}(t),{\mathbf{u}}(t)),k = 1,2, \ldots ,K.}$ At any given time, the decision of the transition logic to switch to another subsystem is governed by transition rules of the form $m(t + h) = {\cal{T}}(m(t),{\mathbf{y}}(t),{\mathbf{u}}(t)).$ Hence, the current input–output variables **y**(*t*), **u**(*t*) plus the current subsystem mode *m*(*t*) determine, via a function ${\cal{T}}$, the next subsystem mode. Without loss of generality, we can rescale the time variable *t* so that *h* = 1. Thus, we can construct the following mathematical model for hybrid dynamical systems

$$\begin{array}{*{20}{l}} {m(t + 1)} \hfill & = \hfill & {{\cal{T}}(m(t),{\mathbf{y}}(t),{\mathbf{u}}(t)),} \hfill \\ {{\mathbf{y}}(t + 1)} \hfill & = \hfill & {{\mathbf{f}}(m(t),{\mathbf{y}}(t),{\mathbf{u}}(t)) = \left\{ {\begin{array}{*{20}{l}} {{\mathbf{f}}_1({\mathbf{y}}(t),{\mathbf{u}}(t)),} \hfill & {{\mathrm{if}}\,m(t) = 1,} \hfill \\ \hfill { \vdots ,} & \hfill\vdots \\ {{\mathbf{f}}_K({\mathbf{y}}(t),{\mathbf{u}}(t)),} \hfill & {{\mathrm{if}}\,m(t) = K.} \hfill \end{array}} \right.} \hfill \end{array}$$

Given the mathematical definition of the hybrid dynamical systems, we can then propose the IHYDE algorithm for discovering such systems from data.

### Inferring subsystems

Let **Y** and **U** denote column vectors containing all the samples of **y**(*t*) and **u**(*t*), respectively, for *t* = 1, 2, …, *M* + 1, where *M* + 1 is the total number of samples. The first step to identify the subsystems is to construct a library **Φ**(**Y**, **U**) of nonlinear functions from the input-output data. The exact choice of nonlinear functions in this library depends on the actual application. For example, for simple mechanical systems, **Φ** would consist of constant, linear and trigonometric terms; in biological networks, **Φ** would consist of polynomial (mass action kinetics) and sigmoidal (enzyme kinetics) terms. Let

$${\mathbf{Y}} = \left[ {\begin{array}{*{20}{c}} | & | & | & | \\ {{\mathbf{y}}(1)} \hfill & {{\mathbf{y}}(2)} & \ldots & {{\mathbf{y}}(M)} \\ | & | & | & | \end{array}} \right]^T,{\mathbf{U}} 	= \left[ {\begin{array}{*{20}{c}} | & | & | & | \\ {{\mathbf{u}}(1)} & {{\mathbf{u}}(2)} & \ldots & {{\mathbf{u}}(M)} \\ | & | & | & | \end{array}} \right]^T,\\ \overline {\mathbf{Y}} 	{\buildrel{\Delta}\over {=}} \left[ {\begin{array}{*{20}{c}} | & | & | & | \\ {\mathbf{{y}}(2)} & {\mathbf{{y}}(3)} & \ldots & {{\mathbf{y}}(M + 1)} \\ | & | & | & | \end{array}} \right]^T.$$

As an illustration, for polynomials (assuming **U** = 0 for notational simplicity), we would have $${\mathbf{\Phi}} ({\mathbf{Y}},{\mathbf{U}}) = \left[ {\begin{array}{*{20}{l}} 1 \hfill & {\mathbf{Y}} \hfill & {{\mathbf{Y}}^{P_2}} \hfill & \cdots \hfill \end{array}} \right]$$ where higher polynomials are denoted as ${\bf{Y}}^{P_2}$, ${\bf{Y}}^{P_3}$, etc. For instance, ${\bf{Y}}^{P_2}$ denotes quadratic nonlinearities^11^:

$${\mathbf{Y}}^{P_2} = \left[ {\begin{array}{*{20}{l}} {y_1^2(1)} \hfill & {y_1(1)y_2(1)} \hfill & \cdots \hfill & {y_n^2(1)} \hfill \\ {y_1^2(2)} \hfill & {y_1(2)y_2(2)} \hfill & \cdots \hfill & {y_n^2(2)} \hfill \\ \vdots & \vdots & \ddots \hfill & \vdots \\ {y_1^2(M)} \hfill & {y_1(M)y_2(M)} \hfill & \cdots \hfill & {y_n^2(M)} \hfill \end{array}} \right].$$

Basically, each column of **Φ**(**Y**, **U**) represents a candidate function for a nonlinearity in *f*_*k*_. Define the residual as

$${\mathbf{Z}} {\buildrel{\Delta}\over {=}} \left[ {\begin{array}{*{20}{l}} {{\mathbf{z}}_1} \hfill & {{\mathbf{z}}_2} \hfill & \ldots \hfill & {{\mathbf{z}}_n} \hfill \end{array}} \right] = \overline {\mathbf{Y}} - {\mathbf{\Phi}}{\mathbf{W}} - {\mathbf{\Xi}},$$

where $${\mathbf{\Xi}} = \left[ {\begin{array}{*{20}{l}} {{\boldsymbol{\xi}}_1, \ldots ,{\boldsymbol{\xi}}_n} \hfill \end{array}} \right]$$, ***ξ***_*i*_ are realizations of i.i.d Gaussian random variable ***ξ*** (i.e., ${\boldsymbol{\xi}}\sim {\cal{N}}(0,\lambda {\mathbf{I}})$) to model uncertainty and noise and $${\mathbf{W}} = \left[ {\begin{array}{*{20}{l}} {{\mathbf{w}}_1} \hfill & {{\mathbf{w}}_2} \hfill & \ldots \hfill & {{\mathbf{w}}_n} \hfill \end{array}} \right]$$ is a matrix of coefficients, where ${\mathbf{w}}_i \in {\mathbb{R}}^{P \times 1}$ and *P* < *M* is the total number of candidate functions in the library. The nonzero elements in **W** determine which nonlinearities are active^9,11^ and the corresponding parameters.

The first objective is to find the sparsest possible **Z** that the most input–output data are fitted, i.e.,

$$\begin{array}{*{20}{l}} {{\mathbf{Z}}^ {\ast}} = {{\mathrm{arg}}\mathop {{{\mathrm{min}}}}\limits_{\mathbf{Z}} \parallel {\mathbf{Z}}\parallel _{{\ell} _0},} \hfill \\ \hskip 23pt {{\hbox{subject to}}:{\mathbf{Z}} = \overline {\mathbf{Y}} - {\mathbf{\Phi}}{\mathbf{W}} - {\mathbf{\Xi}}.} \hfill \end{array}$$

As a result, the indexes of the zero entries of Z^*^ correspond to the indexes for input–output that can be fitted by a single subsystem. This initial idea is an extension of those presented in ref. ^15^, yet the major difference is that we propose a robust Bayesian algorithm that works even for noisy data with better performance (see Supplementary Method 1 for comparison).

Assume, without loss of generality, that the dictionary matrix **Φ** is full rank. A key step is to define a transformation matrix ${\mathbf{\Theta}} \in {\mathbb{{R}}}^{(M - P) \times M}$ whose rows **{Θ**[1, :],$\ldots$,**Θ**[*M* − *P*, :]} form a basis for the left null space of **Φ**. Then, it follows that ${\mathbf{\Theta}}\overline {\mathbf{Y}} = {\mathbf{\Theta}}{\mathbf{Z}} + {\mathbf{\Theta}} {\mathbf{\Xi}}$. Let $\widetilde {\overline {\mathbf{Y}} } {\buildrel{\Delta}\over {=}} {\mathbf{\Theta}}\overline {\mathbf{Y}}$ and **Π** = **ΘΘ**^*T*^, then

1 $$P(\widetilde {\overline {\mathbf{Y}} }|{\mathbf{Z}}) = {\cal{N}}(\widetilde {\overline {\mathbf{Y}} }|{\mathbf{\Theta}} {\mathbf{Z}},\lambda {\mathbf{\Pi}}) \propto {\mathrm{exp}}\left[ { - \frac{1}{{2\lambda }}\left\Vert (\widetilde {\overline {\mathbf{Y}} } - {\mathbf{\Theta}} {\mathbf{Z}})^T{\mathbf{\Pi}}^{ - 1}(\widetilde {\overline {\mathbf{Y}} } - {\mathbf{\Theta}} {\mathbf{Z}})\right\Vert_F^2} \right].$$

Each column of $\widetilde {\overline {\mathbf{Y}} }$ (i.e., $\widetilde {\overline {\mathbf{y}} }_i$) can be identified independently for *i* = 1, ⋯, *n* (let **z**_*i*_ be the *i*th column of **Z**)

2 $$P(\widetilde {\overline {\mathbf{y}} }_i|{\mathbf{z}}_i) = {\cal{N}}(\widetilde {\overline {\mathbf{y}} }_i|{\mathbf{\Theta}} {\mathbf{z}}_i,\lambda {\mathbf{\Pi}}) \propto {\mathrm{exp}}\left[ { - \frac{1}{{2\lambda }}(\widetilde {\overline {\mathbf{y}} }_i - {\mathbf{\Theta}} {\mathbf{z}}_i)^T{\mathbf{\Pi}}^{ - 1}(\widetilde {\overline {\mathbf{y}} }_i - {\mathbf{\Theta}} {\mathbf{z}}_i)} \right].$$

We introduce the Gaussian likelihood in Eq. (2) and the variational prior

$$P({\mathbf{z}}_{i}) = \max_{\gamma_{j} {> } {0}} {\mathop{\prod}\limits_{j}} {\mathcal{N}} (z_{ji}|0,{\gamma}_{j}){\varphi} ({\gamma}_{j}) = \max_{{\mathbf{\Gamma}} {\succ} {0}} {\mathcal{N}}({\mathbf{z}}_{i}|\mathbf{0},{\mathbf{\Gamma}}){\mathop{\prod}\limits_{j}}{\,} {\varphi} ({\gamma}_{j}),$$

where ${\mathbf{\Gamma}} {\buildrel{\Delta}\over {=}} {\mathrm{diag}}\{{\boldsymbol{\gamma}} \}$ and *φ*(*γ*_*j*_) is a nonnegative function. The target is to maximize the marginal likelihood as

3 $${\int} {{\cal{N}} (\widetilde {\overline {\mathbf{y}} }_{i}|{\mathbf{\Theta}} {\mathbf{z}}_{i},\lambda {\mathbf{\Pi}}){\cal{N}}({\mathbf{z}}_{i}|{\mathbf{0}},{\mathbf{\Gamma}}){\mathop {\prod}\limits_{j}} \varphi (\gamma _j)d{\mathbf{z}}_{i}.}$$

Using results in ref. ^9^, we can get the following optimization problem jointly on z_*i*_ and *γ*,

$$\min_{{\mathbf{z}}_{i},{\boldsymbol{\gamma}}} \frac{1}{\lambda }\left( {\widetilde {\overline {\mathbf{y}} }_i - {\mathbf{\Theta}} {\mathbf{z}}_i} \right)^{T}{\mathbf{\Pi}}^{-1}\left( {\widetilde {\overline {\mathbf{y}} }_i - {\mathbf{\Theta}} {\mathbf{z}}_i} \right) + {\mathbf{z}}_i^T{\mathbf{\Gamma}}^{ - 1}{\mathbf{z}}_i + {\hbox{log det}}\ (\lambda {\mathbf{\Pi}} + {\mathbf{\Theta}} {\mathbf{\Gamma}} {\mathbf{\Theta}}^T) + \mathop {\sum}\limits_j {\log} \varphi ({\gamma}_{j}).$$

For the case of uniform priors, let *φ*(*γ*_*j*_) = 1. This program can be formulated as a convex–concave procedure (CCCP), i.e., where the first part of the function

4 $$\begin{array}{*{20}{l}} {u({\mathbf{z}}_i,{\boldsymbol{\gamma}} ) = {\displaystyle\frac{1}{\lambda }}\left( {\widetilde {\overline {\mathbf{y}} }_i - {\mathbf{\Theta}} {\mathbf{z}}_i} \right)^T{\mathbf{\Pi}}^{ - 1}\left( {\widetilde {\overline {\mathbf{y}} }_i - {\mathbf{\Theta}}{\mathbf{z}}_i} \right) + {\mathbf{z}}_i^T{\mathbf{\Gamma}}^{ - 1}{\mathbf{z}}_i} \hfill \end{array}$$

is jointly convex in **z**_*i*_ and ***γ***, and the second part

5 $$\begin{array}{*{20}{l}} {s({\boldsymbol{\gamma}} ) = {\hbox{log det}}\ (\lambda {\mathbf{\Pi}} + {\mathbf{\Theta}} {\mathbf{\Gamma}} {\mathbf{\Theta}}^T)} \hfill \end{array}$$

is concave in ***γ***.

Now the high-level plan is to optimize over each set of variables iteratively based on CCCP, as follows:

6 $${{\mathbf{z}}_i^{k + 1}} = {\rm{arg}} \min_{{\mathbf{z}}_i} u({\mathbf{z}}_i,{\boldsymbol{\gamma}}^k), \\ {{\boldsymbol{\gamma }}^{k + 1}} = {\rm{arg}} \min_{{\boldsymbol{\gamma}} \ge 0} u({\mathbf{z}}_i^k,{\boldsymbol{\gamma}}) + \nabla _{\boldsymbol{\gamma }}s({\boldsymbol{\gamma }}^k)^T{\boldsymbol{\gamma }}.$$

Then we propose our algorithm to solve the above procedure and the pseudo code is summarized in Algorithm 1.

### Algorithm 1

Reweighted $\ell _1$ type algorithm.

1. Initialize the unknown **z**_*i*_ as a unit vector;

2. A tunable hyperparameter *λ*;

3. for *k* = 1, …, *K*_max_ **do**

4.

7 $${\mathbf{z}}_i^{k + 1} = {\rm{arg}} \min_{{\mathbf{z}}_i} \frac{1}{2}\left( {\widetilde {\overline {\mathbf{y}} }_i - {\mathbf{\Theta}} {\mathbf{z}}_i} \right)^T{\mathbf{\Pi}}^{ - 1}\left( {\widetilde {\overline {\mathbf{y}} }_i - {\mathbf{\Theta}} {\mathbf{z}}_i} \right) + \lambda \mathop {\sum}\limits_j {|\alpha _j^k \cdot z_{ji}|};$$

5.  $${\gamma}_{j}^{k + 1} = \left| {\frac{{z_{ji}^{k + 1}}}{{{\alpha }_{j}^{k}}}} \right|,{\alpha } _{j}^{k + 1} = \left({{\boldsymbol{\theta}} _{j}^{T}({\lambda } {\mathbf{\Pi}} + {\mathbf{\Theta}} {\mathbf{\Gamma}}^{k + 1}{\mathbf{\Theta}}^{T})^{ - 1}{{\boldsymbol{\theta}}}_{j}}\right)^{\frac{1}{2}}$$;

6.  **if** a stopping criterion is satisfied **then**

7.   Break.

8:  **end if**

9:  **end for**

This step classifies which time points correspond to which subsystem. The second objective identifies the actual dynamics of each subsystem. The algorithm starts with subsystem *k* (we neglect the index ***k*** for notational simplicity below), which is the one associated with the largest number of time points. Let ${\cal{I}}$ denote those time points associated with subsystem *k*. Once every data point is associated to different subsystems, next, we shall infer the dynamics of every subsystem. We set up yet another sparse regression problem to determine the sparse vectors of coefficients. The sparse coefficients $${\mathbf{W}} {\buildrel{\Delta}\over {=}} \left[ {\begin{array}{*{20}{l}} {{\mathbf{w}}_1} \hfill & \ldots \hfill & {{\mathbf{w}}_n} \hfill \end{array}} \right]$$ of subsystem *k* are then identified by solving the following optimization problem

$${\mathbf{W}}^ \ast = {\mathrm{arg}}\min_{{\mathbf{w}}_i} \frac{1}{2}\parallel \overline {\mathbf{Y}} [{\cal{I}},:] - {\mathbf{\Phi }}[{\cal{I}},:]{\mathbf{W}}\parallel _F^2 + \lambda _{\mathbf{w}}\mathop {\sum}\limits_{i = 1}^n {\left\| {{\mathbf{w}}_i} \right\|_{\ell _1}} ,$$

where *λ*_**w**_ is a hyperparameter that trades off estimation error and model complexity. These hyperparameters are principally tuned using results in Supplementary Method 1. We then remove the data points in ${\cal{I}}$ that has already been fitted by the subsystem. Once we have the new $\overline {\mathbf{Y}}$ and **Θ**, we can solve the same problem with the remaining time points (where the corresponding elements of $\overline {\mathbf{Y}}$ and the corresponding row of **Θ** are nonzero) using the exact same procedure. IHYDE repeats these two steps iteratively until all subsystems have been identified and no data are left. The number of iterations gives the minimum number of subsystems. Further details are found in Supplementary Algorithm 2.

### Inferring transition logics

Once every data point has been classified to different subsystems, define *η*_*i*_(*t*) as the set membership: it equals to 1 only if the subsystem *i* is active at discrete-time *t*, otherwise it equals to 0. These functions are known from the information in the subsystem identification above. Here, we are interested in learning what functions trigger the switch from one subsystem to another. Define also step(*x*), which equals to 1 if *x* ≥ 0, and 0 otherwise. Mathematically, we are searching for a nonlinear function *g*, such that step(*g*(**y**(*t*), **u**(*t*))) specifies the membership. Due to non-differentiability of step functions at 0, we alternatively relax the step function to a sigmoid function, i.e., $\eta _j(t + 1) \approx \frac{1}{{1 + {\mathrm{{e}}}^{ - g({\mathbf{y}}(t),{\mathbf{u}}(t))}}}$^30^, where *j* is a potential subsystem that can jump to at time *t* + 1. If we can parameterize *g*(**y**(*t*), **u**(*t*)) as a linear combination of over-determined dictionary matrix, i.e., $g({\mathbf{y}}(t),{\mathbf{u}}(t)) {\buildrel{\Delta}\over {=}} {\mathbf{\Psi }}({\mathbf{Y}},{\mathbf{U}})[t,:]{\mathbf{v}}$, in which **Ψ** can be constructed similarly to **Φ** in the previous subsection and **v** is a vector of to-be-discovered parameters. We formulate the following optimization problem:

8 $$\min_{\mathbf{v}} \mathop {\sum}\limits_{t = 1}^M {\eta _i} (t)\left\| {\eta _j(t + 1) - \frac{1}{{1 + e^{ - g({\mathbf{y}}(t),{\mathbf{u}}(t))}}}} \right\|_{\ell _2}^2.$$

Further details can be found in Supplementary Algorithm 3. It should be noted that the optimization problem in Eq. (8) is also convex in **v**, which yields a computationally efficient solution.

## Supplementary information

Supplementary Information

Description of Additional Supplementary Files

Supplementary Movie 1

## Data Availability

All data needed to evaluate the conclusions in the paper are available at https://github.com/HAIRLAB/CPSid except datasets from ref. ^30^ (Supplementary Note 3, Example 1–4).

## Code availability

The code implementation is available at https://github.com/HAIRLAB/CPSid.
