# Supplementary material for: Data driven discovery of cyber physical systems
Source: Nat Commun. 2019 Oct 25;10:4894. doi: 10.1038/s41467-019-12490-1 (PMC6814766; doi:10.1038/s41467-019-12490-1)
Supplement: Supplementary file 1 — Supplementary Information [file 41467_2019_12490_MOESM1_ESM.pdf]

# Supplementary Information: Data Driven Discovery of Cyber Physical Systems

Ye Yuan et al.

## SUPPLEMENTARY NOTES

### Supplementary Note 1: Notations

$\mathbb{R}^n$: denotes the n-dimensional Euclidean space.

$\mathbb{Z}$: denotes the set of integers, $\ldots, -1, 0, 1, \ldots$.

$\|\mathbf{x}\|_{\ell_0}$: the $\ell_0$-norm of a vector $\mathbf{x}$, i.e., $\|\mathbf{x}\|_{\ell_0} = \sum_{i=1}^{n} |x_i|^0$ (defining $0^0 = 0$).

$\|\mathbf{x}\|_{\ell_1}$: the $\ell_1$-norm of a vector $\mathbf{x}$, i.e., $\|\mathbf{x}\|_{\ell_1} = \sum_{i=1}^{n} |x_i|$.

$\|\mathbf{x}\|_{\ell_2}$: the $\ell_2$-norm of a vector $\mathbf{x}$, i.e., $\|\mathbf{x}\|_{\ell_2} = (\sum_{i=1}^{n} |x_i|^2)^{1/2}$.

$\|\mathbf{A}\|_F$: the Frobenius-norm of a matrix $\mathbf{A}$, i.e., $\|\mathbf{A}\|_F = (\text{trace}(\mathbf{A}^T\mathbf{A}))^{1/2}$.

$\mathbf{A}$: for a matrix $\mathbf{A} \in \mathbb{R}^{M\times N}$, $\mathbf{A}[i,j] \in \mathbb{R}$ denotes the element in the $i^{th}$ row and $j^{th}$ column, $\mathbf{A}[i,:] \in \mathbb{R}^{1\times N}$ denotes its $i^{th}$ row, $\mathbf{A}[:,j] \in \mathbb{R}^{M\times 1}$ denotes its $j^{th}$ column.

$\boldsymbol{\alpha}$: for a column vector $\boldsymbol{\alpha} \in \mathbb{R}^{N\times 1}$, $\boldsymbol{\alpha}[i]$ denotes its $i^{th}$ element.

$\mathbf{I}_k$: a $k$-dimensional identity matrix.

$\mathbf{0}_k$: a $k$-dimensional zero matrix.

### Supplementary Note 2: Introduction to Hybrid Dynamical Systems

A dynamical system describes how state variables (typically physical quantities) evolve with respect to time. Following definitions in [1], we define three types of variables.

- continuous state variables: if the state variable takes value in $\mathbb{R}^n$ for $n \geq 1$.
- discrete state variables: if the state variable takes value in a finite set, for example, $\{1, 2, 3, \ldots\}$.
- hybrid state variables: if a part of the state variables are continuous and the other discrete.

Based on the time set over which the state evolves, we classify the dynamical systems as:

- continuous time: if the set of time is a subset of the real line $\mathbb{R}$. Normally we use $t \in \mathbb{R}$ to denote the continuous time. The evolution of the state-variables in continuous time can be described as ordinary differential equations.
- discrete time: if the set of time is a subset of the integers. Normally we use $k \in \mathbb{Z}$ to denote discrete time. The evolution of the state-variables in discrete time can be described as difference equations.

A hybrid dynamical system $\mathcal{H}$, is defined as a tuple, $\mathcal{H} = (\mathcal{W}, \mathcal{M}, \mathcal{F}, \mathcal{T})$ with the following definitions:

- $\mathcal{W}$ defines a subspace in $\mathbb{R}^{m+n}$ for input-output variables $\mathbf{u}(t) \in \mathbb{R}^m, \mathbf{y}(t) \in \mathbb{R}^n$;
- $\mathcal{M}$ defines a countable, discrete set of modes in which only a single mode, $m(t) \in \{1, 2, \ldots, K\}$, is occupied at a given time;
- $\mathcal{F}$ defines a countable discrete set of first-order differential equations:
$$\mathcal{F} = \left\{ \frac{d\mathbf{y}(t)}{dt} = \mathbf{F}_k\left(\mathbf{y}(t), \mathbf{u}(t)\right)\right) \mid k = 1, 2, \ldots, K \right\}.$$
- $\mathcal{T}$ defines a countable discrete set of transitions, where $\mathcal{T}_{i \to j}$ denotes a Boolean expression that represents the condition to transfer from mode $i$ to $j$.

The signals $\mathbf{y}(t)$ and $\mathbf{u}(t)$ are sampled at a rate $h > 0$, i.e. sampled at times $0, h, 2h, 3h....$ For fast enough sampling (or low $h$), standard system identification typically obtains first a discrete-time system, and then coverts it to a continuous-time system [2]. One of the simplest methods to approximate derivatives is to consider

$$\frac{d\mathbf{y}(t)}{dt} \approx \frac{\mathbf{y}(t+h) - \mathbf{y}(t)}{h},$$

which yields the discrete-time system

$$\mathbf{y}(t+h) = \mathbf{y}(t) + h\ \mathbf{F}_k(\mathbf{y}(t), \mathbf{u}(t)) \triangleq \mathbf{f}_k(\mathbf{y}(t), \mathbf{u}(t)), \qquad k \in \{1, 2, \ldots, K\}.$$

For simplification of notation, assume the system can be written as

$$\mathbf{y}(t+h) = \mathbf{f}_k(\mathbf{y}(t), \mathbf{u}(t)) \triangleq \mathbf{I}_k(\mathbf{y}(t)) + \mathbf{h}_k(\mathbf{u}(t)), \qquad k \in \{1, 2, \ldots, K\}.$$

Hence, the class of systems considered is discrete-time, Markovian and nonlinear. While this is already a very rich class of systems, it can be easily extended to more general nonlinear systems, including, for example, dynamics of non-separable nonlinear functions of $(\mathbf{y}(t), \mathbf{u}(t))$.

Without loss of generality, we can rescale the time variable $t$ so that $h = 1$. Thus, we can construct a mathematical model for hybrid dynamical systems

$$\begin{aligned} m(t+1) &= \mathcal{T}(m(t), \mathbf{y}(t), \mathbf{u}(t)), \\ \mathbf{y}(t+1) &= \mathbf{f}(m(t), \mathbf{y}(t), \mathbf{u}(t)) = \begin{cases} \mathbf{f}_1(\mathbf{y}(t), \mathbf{u}(t)), & \text{if } m(t) = 1, \\ \vdots, & \vdots \\ \mathbf{f}_K(\mathbf{y}(t), \mathbf{u}(t)), & \text{if } m(t) = K. \end{cases} \end{aligned} \tag{1}$$

**Example 1** *Consider again the temperature control system in Supplementary Figure 1, consisting of a heater and a thermostat. The variables in this model are the room temperature $y(t) \in \mathbb{R}$ and the operating mode of the heater (on or off). Assuming a sampling time of $h > 0$, we obtain the following approximate difference equations (discretized from an ordinary differential equation) for the temperature*

$$\textit{Subsystem } 1\ (\textit{heat off}): \ \frac{y(t+1) - y(t)}{h} \approx -ay(t), \Rightarrow y(t+1) = (1 - ah)y(t).$$
$$\textit{Subsystem } 2\ (\textit{heat on}): \ \frac{y(t+1) - y(t)}{h} \approx -a(y(t) - 30), \Rightarrow y(t+1) = (1 - ah)y(t) + 30ah.$$

*These two equations model how the temperature changes under the heater off or on, respectively. The transition logics between the two subsystems are*

$$\textit{Transition logic from subsystem 1 to 2 } \mathcal{T}_{1\to 2}: \ y \leq 19,$$
$$\textit{Transition logic from subsystem 2 to 1 } \mathcal{T}_{2\to 1}: \ y \geq 21,$$

*representing the controller of the operating mode of the heater. Given this hybrid dynamical system, we can study its stability or simulate it to check possible state trajectories. Note that, in practice, hybrid dynamical systems, such as this one, are usually unknown or only partially known. The goal of this paper is to infer both the above subsystems and the transition logics (Fig. 1(a)) from only time-series data of the temperature in Fig. 1(c).*

**Supplementary Note 3: Examples**

This section applies IHYDE to more than ten examples ranging from power systems to robotics, showcasing the wide range of applicability of the proposed IHYDE method. The data structure of each dataset is shown in Supplementary Table 3.

**Example 1: Hysteresis Relay**

One of the most common Cyber Physical Systems is the Hysteresis Relay. It is found, for example, in almost all thermostats: the heater is turned on when the temperature is below a threshold, and turned off when the temperature is above another threshold. Typically, the low and high temperature switching are different to avoid frequent switching, which could damage the system. The Hysteresis Relay can be found in physical, chemical, engineering and biological applications.

The datasets for discovery are generated by Ly et. al. in [3]. The additive noise level varies from 0% to 6% in 2% increments, i.e., $N_p = \frac{\sigma_{\text{noise}}}{\sigma_y} \times 100\%$, where $\sigma_{\text{noise}}$ is the noise variance and $\sigma_y$ is the variance of the measurement. We apply the proposed IHYDE to data generated by an unknown Hysteresis Relay to discover its hybrid dynamical model (shown in Supplementary Figure 3a). Supplementary Table 4 shows the detailed information about this data. The discovered systems are shown in Supplementary Table 5 and Supplementary Table 6 using 2000 data-points respectively.

Using IHYDE for subsystems identification, we successfully identify that there are only two subsystems. In addition, the two identified subsystems are consistent with or close to the true ones from both noiseless and noisy data. Specifically, with or without redundant dictionary functions, we are able to identify the true systems, achieving very similar discovery results. This, in other words, demonstrates that the IHYDE is able to discover the true subsystems, the number of subsystems together with parameterizations of every subsystem.

Once all subsystems have been identified and all data points have been classified, IHYDE identifies the transition logics between subsystems. When there is no redundant dictionary function (i.e., when prior knowledge is available about the structure of transition logics), IHYDE is able to precisely identify the correct transition logics. The identified results are shown in Supplementary Table 7. When there exists redundant dictionary functions, IHYDE still successfully identifies the transition logics. The identified results are shown in Supplementary Table 8.

**Example 2: Continuous Hysteresis Loop**

A Continuous Hysteresis Loop is yet another classical hybrid system– here we use the Preisach model [3] for data simulation. In this setup, each subsystem has its own input-output behavior while the transitions occur when the input hits certain thresholds as shown in Supplementary Figure 3b. The detailed information is summarized in Supplementary Table 9. We apply the IHYDE to reverse engineering the Continuous Hysteresis Loop using 2000 data points generated by [3].

The identified systems are shown in Supplementary Table 10 and Supplementary Table 11. In contrast with the previous Hysteresis Relay example, the IHYDE will obtain false classification results as the noise level increases. Yet, IHYDE is still able to identify the actual subsystem dynamics up to some precision.

Once all subsystems have been identified and all data points have been classified, IHYDE identifies the transition logics between subsystems. Supplementary Table 12 shows that IHYDE can find the true transition logics without redundant dictionary functions. Even when there exists redundant basis functions, IHYDE is able to precisely identify the correct transition logics. The identified results are shown Supplementary Table 13.

**Example 3: Phototaxis Robot**

Consider a Phototaxis Robot with a hybrid dynamical system model shown in Supplementary Figure 3c [4], the robot has phototaxis movement: it approaches, avoids, or remains stationary depending on the color of light. As described in [3], the output $y$ is velocity of the robot. There are five inputs: $u_1$ and $u_2$ are the absolute positions of the robot and the light, respectively, while $\{u_3, u_4, u_5\}$ is a binary, one-hot encoding of the light color, where 0 indicates the light is off and 1 indicates the light is on.

Similar to previous examples, 2000 data points are used. The detailed information is shown in Supplementary Table 14 and Supplementary Table 15. Even the IHYDE obtains false data classification results as the noise level increases (shown in Supplementary Table 16 and in Supplementary Table 17). Yet, IHYDE is still able to identify the actual subsystem dynamics without redundant dictionary functions when noise intensity is low. When there exists redundant dictionary functions, IHYDE can identify all the subsystems when there is

no noise. When noise level increases, IHYDE still identifies the right number of subsystems, except the third identified subsystem is different from the true one, i.e., $y = 0$.

Again, once all subsystems have been identified and all data points have been classified, IHYDE identifies the transition logics between subsystems. IHYDE is able to precisely identify the correct transition logics both when there is no redundant dictionary function (Supplementary Table 18) and when there is (Supplementary Table 19). At a first glance, the inferred transition logic is different from the actual ones. Given $u_3, u_4, u_5$ are binary values, still, the inferred transition logics are equivalent to the actual ones.

**Example 4: Nonlinear Hybrid System**

Consider the Nonlinear Hybrid System shown in Supplementary Figure 3d. This example is a system without any physical counterpart, yet it is useful to evaluate the capabilities of IHYDE for finding nonlinear expressions. The system consists of three subsystems, where all of the behaviors and transition logics consist of nonlinear equations which cannot be modeled via parametric regression. All the expressions are a function of the variables $u_1$ and $u_2$, the discriminant functions are not linearly separable and the transitions are modally dependent.

Detailed information for this system is summarized in Supplementary Table 20 and Supplementary Table 21. Using 2000 data points in dataset generated by [3], the identified results are shown in Supplementary Table 22 and Supplementary Table 23. The IHYDE successfully identifies that there are three subsystems that generate the datasets. In addition, the three identified subsystems are consistent with or close to the true ones from both noiseless and noisy data. IHYDE precisely identifies the correct transition logics with and without redundant dictionary functions (Supplementary Table 24 and Supplementary Table 25).

**Example 5: Autonomous Car**

This example presents the results of IHYDE applying to an autonomous car built in our lab. The autonomous car consists of a body, a MK60t board, a servo motor, tow driving motors and a camera. During execution, the embedded camera captures the upcoming road

layouts to check whether there is an upcoming straightaway or curve. Naturally, the car will drive faster on straightaways and slower on the curves.

Based on this design principle, we would like to design a hybrid dynamical system with two subsystems and simple transition logic to realize this goal as shown in the right panel of Supplementary Figure 5. The car measures current speed by encoder and calculates the $\Delta u$, a control input to the motor. The speed control strategy is based on an incremental PI control algorithm, which is widely used in control systems. The incremental PI algorithm is developed from position PI algorithm. The position PI model is described as below and can be seen in Supplementary Figure 4. $r(t)$ represents the input of the whole system (the expected speed $v_{\text{expect}}(t)$) and $c(t)$ represents the output of the whole system (the real speed observed $v(t)$).

In the figure, $u(t)$ is the output of the controller and it can be calculated from $e(t)$:

$$u(t) = P\left[e(t) + \frac{1}{T_{\mathrm{I}}}\int_0^t e(t)dt\right], \tag{2}$$

where $P$ is the constant for the proportional control, $T_{\mathrm{I}}$ is the time constant for the integral control. In the Laplace domain, Eq. (2) is equivalent to $U(s) = D(s)E(s)$, where $U(s)$ and $E(s)$ are the Laplace transform of $u(t)$ and $e(t)$ respectively, $D(s)$ represents the transfer function of the controller:

$$D(s) = \frac{U(s)}{E(s)} = P\left(1 + \frac{1}{T_{\mathrm{I}}s}\right). \tag{3}$$

Since the controller is implemented by a computer, it must be first converted to discrete time. The integral can be approximated by

$$\int_0^t e(t)dt \approx \sum_{i=0}^{k} Te(i) \Rightarrow \frac{de(t)}{dt} \approx \frac{e(k) - e(k-1)}{T}. \tag{4}$$

So we obtain the following control law

$$u(k) = P\left[e(k) + \frac{T}{T_{\mathrm{I}}}\sum_{i=0}^{k} e(i)\right]. \tag{5}$$

The position PI algorithm is usually approximated by an incremental PI algorithm:

$$\Delta u(k) \triangleq u(k) - u(k-1) = P[e(k) - e(k-1)] + Ie(k), \tag{6}$$

where $I \triangleq \frac{PT}{T_I}$. In the autonomous car example, we have

$$r(k) = v_{\text{expect}}(k),\ c(k) = v(k),\ e(k) = v_{\text{expect}}(k) - v(k). \tag{7}$$

Here $v_{\text{expect}}(k)$ is the expected velocity depending on whether there is an upcoming straightaway or curve from the camera. We set up a faster velocity on straightaways and slower one on the curves. Substituting $e(k)$ into the Eq. 6, we obtain

$$\Delta u(k) = P[v(k-1) - v(k)] + P[v_{\text{expect}}(k) - v_{\text{expect}}(k-1)] + I[v_{\text{expect}}(k) - v(k)]. \quad (8)$$

When the car changes its expected velocity, this could lead to a more complicated hybrid dynamical system than we would design as shown in Supplementary Figure 5. This side effect is due to the abrupt switching and discretization. In practice, we normally neglect these subsystems in the modeling, analysis and design. The flow chart of the PI control algorithm is shown in Supplementary Figure 6.

Next, we demonstrate how IHYDE can help in the design process. In the first experiment, the autonomous car failed to drive through the track. We collected the experimental data and used IHYDE to discover the failed system. We compared the discovered system model with the to-be designed one and found an implementation error that led the system to failure. We expected a higher speed when the car is running in a straight line and a lower speed while it is running on a curve. The model from the failed experiments showed that the transition logistics should be reversed as shown in Supplementary Figure 7 and Supplementary Table 27. We fixed the bug and as a result the autonomous car was able to run through the track. Finally, as a validation, we collected the data shown in Supplementary Table 26 and repeated the modeling process in Supplementary Table 28 and Supplementary Table 29.

In summary, IHYDE successfully reverse engineered the control strategy of the CPS. Additionally, we deliberately swapped the straightway and curve speeds to mimic a software bug. The modeled system immediately pinpointed the location of the faulty software and yielded important information for debugging the system.

**Example 6: Chua's Circuit**

In this subsection and the next one, we shall apply IHYDE to data that is obtained from experiments shown in Supplementary Table 30 and Supplementary Table 31. We bulit a Chua's circuit (see Supplementary Figure 8) in our lab which is the simplest electronic circuit that exhibits classic chaotic behavior. It consists of an inductor, two capacitors, a passive resistor and an active nonlinear resistor as show in Supplementary Figure 9a which

fits the condition for chaos with the least components. The most important active nonlinear resistor is a conceptual component and the resistor can be built with operational amplifiers and linear resistors. The current-voltage characteristics of the nonlinear resistor are plotted in Supplementary Figure 9b.

By design, the current-voltage relationship can be described as follows:

$$i(V) = \begin{cases} aV + (b-a)(V-E), & V > E, \\ aV, & -E < V < E, \\ aV + (b-a)(V+E), & V < -E, \end{cases} \tag{9}$$

or equivalently

$$i(V) = bV + \frac{1}{2}(a-b)(|V+E| - |V-E|). \tag{10}$$

In both equations, $a$, $b$, $E$ are parameters depicted in Supplementary Figure 9b.

The nonlinear resistor can be built using the circuit realization as shown in Supplementary Figure 9c. From KCL and KVL, we obtain

$$\begin{aligned} \frac{C_1 dV_1}{dt} &= \frac{V_2 - V_1}{R} - i(V_1), \\ \frac{C_2 dV_2}{dt} &= \frac{V_1 - V_2}{R} + I_L, \\ -L\frac{dI_L}{dt} &= V_2, \end{aligned} \tag{11}$$

where

- $C_1$: Capacity of Capacitor 1. $C_2$: Capacity of Capacitor 2. $L$: Inductance of the Inductor.
- $V_1$ : Voltage through Capacitor 1. $V_2$: Voltage through Capacitor 2.
- $I_L$: Current through Inductor. $i$: Current through the nonlinear resistor.
- $a$: the slope of low voltage for the nonlinear resistor. $b$: the slope of high voltage for the nonlinear resistor.

Then we introduce a number of variables to simplify the above equations:

$$y_1 = \frac{V_1 \tau}{E},\ y_2 = \frac{V_2 \tau}{E},\ y_3 = \frac{I_L \tau}{E}, \tag{12}$$

where $E$ is the threshold voltage for the nonlinear resistor and $\tau$ is a threshold of the Chua's circuit, which equals to $E$ in this experiment. Let

$$\alpha = \frac{1}{RC_1},\ \beta = \frac{1}{L},\ f(y)|_{y=\frac{\tau}{E}x} = \frac{R\tau}{E} i(x), \tag{13}$$

we can obtain the following equations

$$\frac{dy_1}{dt} = \alpha[y_2 - y_1 - f(y_1)],$$
$$RC_2\frac{dy_2}{dt} = y_1 - y_2 + Ry_3, \tag{14}$$
$$\frac{dy_3}{dt} = -\beta y_1,$$

where

$$f(x) = \begin{cases} k_1x + b_1, & x < -\tau, & (15a) \\ k_0x, & -\tau < x < \tau, & (15b) \\ k_1x + b_2. & x > \tau, & (15c) \end{cases}$$

with

$$k_0 = Ra,\ k_1 = Rb,\ b_1 = R(a-b)\tau,\ b_2 = R(a-b)\tau.$$

The behavior of the system will be changed between chaos and non-chaos depending on the value of $R$. Each mode in Eq. (15a), Eq. (15b) and Eq. (15c), corresponds to subsystem 1, subsystem 2 and subsystem 3 respectively. We focus on the discovery of the first equation in Eq. (14) and only collect the value of $y_1$ and $y_2$. The output data from the Chua's circuit can be seen in Supplementary Figure 9d.

From the true parameters in Supplementary Table 32, we can compute the true coefficients of $f(x)$ to determine $\frac{dy_1}{dt}$:

$$10^{-5}\frac{dy_1}{dt} = \begin{cases} 1.0858y_2 - 0.2115y_1 - 0.5349, & y_1 < -1.5, & (16a) \\ 1.0858y_2 + 0.1451y_1, & -1.5 < y_1 < 1.5, & (16b) \\ 1.0858y_2 - 0.1994y_1 + 0.5168, & y_1 > 1.5. & (16c) \end{cases}$$

The algorithm accurately infers the form of Eq. (16c) from the data as shown in Supplementary Table 33.

**Example 7: Monitoring of Industrial Processes**

The next example illustrates how IHYDE can be used for fault detection in mechanical engineering. Experiments conducted on a wind turbine system experimental platform [5] shown in Supplementary Figure 10 are used to verify its effectiveness.

This system contains a power supply of 380V, an inverter, a motor, a gearbox, a power generator, and a load. The platform is used to simulate the process of air flow through wind

turbines to generate electricity. Specifically, the motor with a gear reducer of 20 : 1 ratio supplies the generator with mechanical power through the gearbox. In this experiment, we adopt the mode of gearbox inversion to simulate the operation of wind turbine system. The gearbox has been widely used to provide speed and torque conversions from a motor to generator in wind turbines [6]. This system has a gearbox with three shafts, i.e., shaft with low speed, shaft with intermediate speed and shaft with high speed. The load consumes the power generated by the generator. We can measure the root-mean-square current and voltage of the motor from the inverter. The current of generator can be captured by oscilloscope and its voltage is measured through multimeter. We measure the voltage of the load in the same way.

We perform experiments under normal and faulty conditions. Both experiments are performed in the situation where the generator speed is 200 revolutions per minute and the load is 1.5 KNm. One-third of the tooth width cut off from the gear tooth on the high-speed shaft is considered as the faulty condition. In the normal operation, the motor power is 383.01W and the generator power is 53.28W; the load voltage is 75V in the faulty condition.

Two sets of current data are measured at the frequency of 1000Hz connected in series for identification. The first dataset contains $19,995$ data points sampled under normal operating condition, the other has $20,000$ data points obtained from the faulty condition. Then, we down-sample at the period of 0.3s and denote as $i(k)$ in which $k = 1, \dots, 133$. Supplementary Table 34 shows the detailed information for this data.

As described in the main text, here we used an online monitoring scheme. We construct the output $\mathbf{y} \in R^{64\times 1}$, including 61 current measurements from 1.8s to 19.8s in the normal condition and 3 data points from 20.1s to 20.7s in the faulty condition when the mismatch is large. Specifically

$$\mathbf{y} = \begin{bmatrix} i(7) & i(8) & \dots & i(70) \end{bmatrix}^T .$$

With the candidate terms of the polynomial combinations of $i(k), \dots, i(k+5)$ up to second order, we construct a dictionary matrix $\mathbf{\Phi} \in R^{64\times 28}$ as follows:

$$\mathbf{\Phi} = \begin{bmatrix} 1 & i(6) & \cdots & i(1) & i^2(6) & \dots & i^2(1) \\ \vdots & \vdots & \vdots & \vdots & \vdots & \vdots & \vdots \\ 1 & i(69) & \dots & i(64) & i^2(69) & \dots & i^2(64) \end{bmatrix} .$$

It is worth mentioning that this experiment is a one-shot experiment. However, many state of the art machine learning methods [7] need (a large number of) historical data including its labels (healthy or faulty), while industrial data is often unlabeled and scarce. Therefore, these algorithms are not a good solution to this type of one-shot industrial problem. And, this example demonstrates the capability of IHYDE to the identification of the fault in industrial processes. Supplementary Figure 11 shows that the relative fitting error ratio is small. The identified results and details of the IHYDE are presented in Supplementary Table 35, which shows that the identified time for the fault occurrence is the same as the real fault time 68. We only use three fault points to realize the fault detection.

**Example 8: Power Grid Fault Detection**

The next example illustrates how IHYDE can be used in real-time monitoring applications. Consider the fault detection problem in a smart grid. The design of monitoring schemes to diagnose anomalies caused by unpredicted or sudden faults on power networks is of great importance.

Here we consider a benchmark power network, IEEE 14 bus test system. Suppose the line connecting buses 6 and 12 disconnects at time 31, changing the admittance between these two buses to zero. We simulate the data summarized in Supplementary Table 36 and only pass the data to IHYDE without other information. IHYDE can immediately detect the occurrence of this event and estimate the new admittance matrix using the next 10 measurements. The identified results and parameters are summarized in Supplementary Table 37. It successfully discovers two different subsystems from data and pinpoints the difference in the discovered subsystems which corresponds to the fault. Given the frequency at which PMUs sample voltage and current, IHYDE is able to locate the fault in a few hundred milliseconds after the event occurs, enabling the operators to detect the event, identify its location, and take remedial actions in near real-time.

**Example 9: Identification of Real-time Models for Smart Grid**

This example illustrates how the proposed IHYDE method can be used to solve the identification problem in smart grid, which contains two major parts, that is, smart infras-

tructure system and smart management system [8]. It is crucial to obtain real-time models for smart management system to achieve resilient and efficient operations. Accurate model information is not only necessary for daily operation and scheduling, but also critical for other advanced techniques such as state estimation and optimal power flow computation. However, such information is not always available in distribution systems due to frequent model changes. For example, the model of a distribution system connected with photovoltaic panels maybe change once every eight hours [9]. Furthermore, some unexpected events, such as line faults and unreported line maintenance, can lead to model changes. Moreover, network reconfiguration (such as switch action for balancing loads and avoiding voltage sag) happens frequently in distribution systems. Therefore, model identification in real-time is meaningful.

We apply IHYDE to identify network models in real-time and to infer transition logics for model changes using data from advanced metering infrastructure. The used data detailed in Supplementary Table 38 is generated with the 33-bus benchmark distribution system [10]. Consider the situation where the increase of loads at some remote nodes of a feeder causes the voltage sag, an operator then takes switch action for load balancing and voltage regulation. Supplementary Figure 12 depicts the switching topologies and the real transition logics. The detailed actions and switching time are shown in Supplementary Table 39. Measurements are generated via solving nonlinear power flow equations using MATPOWER toolbox [11] in MATLAB.

Suppose that we can measure all the active and reactive power consumption, voltage magnitudes and phases of the nodes, denoted by $\mathbf{Y}$ as follows

$$\mathbf{Y} = \begin{bmatrix} P_1(1) & Q_1(1) & V_1(1) & \delta_1(1) & \cdots & P_{33}(1) & Q_{33}(1) & V_{33}(1) & \delta_{33}(1) \\ \vdots & \vdots & \vdots & \vdots & \ddots & \vdots & \vdots & \vdots & \vdots \\ P_1(M) & Q_1(M) & V_1(M) & \delta_1(M) & \cdots & P_{33}(M) & Q_{33}(M) & V_{33}(M) & \delta_{33}(M) \end{bmatrix},$$

where $V_i(t)$, $\delta_i(t)$, $P_i(t)$ and $Q_i(t)$ are the voltage magnitude, voltage phase, active and reactive power of Bus $i$ at time instant $t$, respectively. The total sampling time $M$ is set to 180 in the following simulation. Supplementary Table 38 shows the detailed information of this data.

For each node, we apply IHYDE to identify the responding column of the admittance matrix. The output $\mathbf{y}_i \in R^{2M\times 1}$ of Bus $i$ is $\mathbf{y}_i = [P_i(1), Q_i(1), \cdots, P_i(M), Q_i(M)]^T$. The

quadratic terms for the voltages are chosen as the dictionary function based on Ohm's law and power factor; sine and cosine terms are also considered, since there are voltage angle differences for delivering power from one bus to another bus. The $j^{th}$ column of dictionary matrix $\mathbf{\Phi}^i \in R^{2M\times 66}$ is as follows:

$$\boldsymbol{\phi}_j = [V_i(1)V_j(1)\cos\delta_{ij}(1), V_i(1)V_j(1)\sin\delta_{ij}(1), \cdots, V_i(M)V_j(M)\cos\delta_{ij}(M), V_i(M)V_j(M)\sin\delta_{ij}(M)]^T,$$

where $\delta_{ij}(t) = \delta_i(t) - \delta_j(t)$ denotes the phase difference between nodal voltages of Bus $i$ and $j$ at time instant $t$.

Supplementary Table 40 shows the identified results and the detailed tuning parameters of the proposed algorithm. For example, at Bus 12, the maximum relative identification ratio of Base configuration and Changed configuration are 0.00057% and 0.00182%, respectively. The identified admittance matrices at time instants $31, 61, 91, 121, 151$ are very different from that of the previous moments, which indicates the model switching. The results demonstrate that IHYDE can identify the models accurately and pinpoint model switching time correctly. We add the difference of voltage magnitude between different times, denoted by $\Delta V = V(t) - V(t-1)$, into dictionary matrix for logic identification. Supplementary Table 41 indicates that the identified logic is consistent with the real logic with small error. Specifically, the result of $\mathcal{T}_{1\to 2}$ (switching from subsystem 1 to 2) reveals that the voltage drop of node 10 at feeder 3 are more than 0.0500 at time 30, subsequently, switch action is taken to avoid sharp voltage drop. The tie switch between Bus 12 and 22 is closed, while the sectionalizing switch between Bus 11 and 12 opens. This is consistent with our preset reason that loads at Bus $9, 10, 11$ increase rapidly at time 30. There are many indistinct physical phenomenons in actual power system and IHYDE can be utilized to help engineers understand the hidden mechanism behind it.

**Example 10: Discovery of Human Atrial Action Potential Models**

In this section, we apply IHYDE to a human atrial action potential (AP) model proposed in [12] to show the applicability of IHYDE to the discovery in biology. The parameters of the human atrial AP model are determined based on the data that is directly measured on human atrial cells and that is from AP model of guinea pig ventricular and rabbit atrial. The AP model can reproduce a variety of observed AP behaviors and provide potential

insights into its underlying ionic mechanisms. The human atrial AP and ionic currents that underlie its morphology are of great importance to our understanding and prediction of the electrical properties of atrial tissues under normal and pathological conditions.

Specifically, the cell membrane is modeled as a capacitor connected in parallel with variable resistances and batteries representing the ionic channels and driving forces. The AP model includes 21 differential equations and 163 parameters in total (see [12] for detailed information). The membrane potential formulation is $\frac{dV}{dt} = \frac{-(I_{\text{ion}}+I_{\text{st}})}{C}$, where $V$ is membrane potential, and $C$ is the constant total membrane capacitance. $I_{\text{ion}}$ and $I_{\text{st}}$ are the total ionic current and stimulus current flowing across the membrane, respectively.

Supplementary Figure 13 shows that the action potential generated by the AP model through voltage clamp method is a spike-and-dome morphology commonly observed in human atrial AP recordings. We apply the stimulation current with 2 ms pulses of 2 nA amplitude across the cell membrane every 1000 ms. To check the performance of the IHYDE method, we focus on two representative equations about gating variables $x_1$ and $x_2$ with time-varying parameters as follows:

$$\frac{dx_1}{dt} = \alpha_1 - (\alpha_1 + \rho_1)x_1, \tag{17}$$

$$\frac{dx_2}{dt} = \alpha_2 - (\alpha_2 + \rho_2)x_2, \tag{18}$$

where $x_1$ and $x_2$ are fast and slow inactivation gating variables for fast inward $Na^+$ current, respectively. For convenience, we present the time-varying parameters $\alpha_1, \alpha_2, \rho_1, \rho_2$:

$$\alpha_1 = \begin{cases} \alpha_{11} \triangleq 0.135\exp(-\frac{V+80}{6.8}), & V < -40, \\ \alpha_{12} \triangleq 0, & V \geq -40, \end{cases}$$

$$\alpha_2 = \begin{cases} \alpha_{21} \triangleq [-1.2714 \times 10^5 \exp(0.2444V) - \\ \quad 3.474 \times 10^{-5} \exp(-0.04391)]\frac{V+37.78}{1+\exp[0.311(V+79.23)]}, & V < -40, \\ \alpha_{22} \triangleq 0, & V \geq -40, \end{cases}$$

$$\rho_1 = \begin{cases} \rho_{11} \triangleq 3.56\exp(0.079V) + 3.1 \times 10^5 \exp(0.35V), & V < -40, \\ \rho_{12} \triangleq \{0.13[1 + \exp(-\frac{V+10.66}{11.1})]\}^{-1}, & V \geq -40, \end{cases}$$

$$\rho_2 = \begin{cases} \rho_{21} = 0.1212\frac{\exp(-0.01052V)}{1+\exp[-0.1378(V+40.14)]}, & V < -40, \\ \rho_{22} = 0.3\frac{\exp(-2.535\times10^{-7}V)}{1+\exp[-0.1(V+32)]}, & V \geq -40. \end{cases}$$

When the gating variables $x_1$ and $x_2$ are equal to 1, the fast inward $Na^{2+}$ current is inactive

completely. Supplementary Figure 14 depicts that they gradually rise to their resting values 0.9775 and 0.9649 after stimulus.

It is clearly observed that membrane voltage gradually returns to its stable resting potential −81mV after the stimulation from Supplementary Figure 13. During the process, the dynamics for gating variables $x_1$ and $x_2$ has been switched as shown in Supplementary Figure 14 when the membrane voltage $V$ goes through −40 mV. Supplementary Table 42 summarizes the data structure that is used for identification. We apply IHYDE to discover the different models and the transition logics only using measurements. The first-order differential values of $x_1$ and $x_2$ are considered as their output, respectively. For instance, we down-sample the differential value of $x_1$ during $120 - 500$ ms as its output

$$\mathbf{y}_1 = \left[\frac{dx_1(120)}{dt}, \frac{dx_1(120+h)}{dt}, \cdots, \frac{dx_1(499.8)}{dt}\right]^T \in R^{1267\times 1}.$$

The sampling period $h$ is set to 0.3 ms, and there are 1267 data points for each variable. The dictionary matrix of gating variables $x_1$ and $x_2$, denoted by $\mathbf{\Phi}^1$ and $\mathbf{\Phi}^2$, respectively, are established based on the terms of the above equations

$$\mathbf{\Phi}^1 = \begin{bmatrix} \exp(-\frac{V(t_1)+80}{6.8}) & x_1(t_1)\exp(-\frac{V(t_1)+80}{6.8}) & x_1(t_1)\rho_{11}(t_1) & x_1(t_1)\rho_{12}(t_1) \\ \vdots & \vdots & \vdots & \vdots \\ \exp(-\frac{V(t_M)+80}{6.8}) & x_1(t_M)\exp(-\frac{V(t_M)+80}{6.8}) & x_1(t_M)\rho_{11}(t_M) & x_1(t_M)\rho_{12}(t_M) \end{bmatrix},$$

$$\mathbf{\Phi}^2 = \begin{bmatrix} \alpha_{21}(t_1) & x_2(t_1)\alpha_{21}(t_1) & x_2(t_1)\frac{\exp(-0.01052V(t_1))}{1+\exp[-0.1378(V(t_1)+40.14)]} & x_2(t_1)\frac{\exp(-2.535\times 10^{-7}V(t_1))}{1+\exp[-0.1(V(t_1)+32)]} \\ \vdots & \vdots & \vdots & \vdots \\ \alpha_{21}(t_M) & x_2(t_M)\alpha_{21}(t_M) & x_2(t_M)\frac{\exp(-0.01052V(t_M))}{1+\exp[-0.1378(V(t_M)+40.14)]} & x_2(t_M)\frac{\exp(-2.535\times 10^{-7}V(t_M))}{1+\exp[-0.1(V(t_M)+32)]} \end{bmatrix},$$

where $t_1$ and $t_M$ are 120 and 499.8 ms, respectively.

The identified results and the detailed parameters are summarized in Supplementary Table 43. We can see that IHYDE identifies the subsystem and pinpoints the changing time correctly. The identified logic (see Supplementary Table 44 and Supplementary Table 45) for both gating variables are $V < -40.0093$, which is very close to the real logic $V \leq -40$.

Next, we repeat the modeling of this system with the assumption that the choice of dictionary functions is unclear and/or the domain knowledge is lacking. In such cases, we consider a canonical dictionary function, such as polynomials approximations. The results are summarized in Supplementary Table 46. IHYDE can still detect the transition points.

However, the nonlinear dynamics are different than the true ones: as expected, it identifies instead a polynomial approximation of the original nonlinear dynamics. While these dynamics can still be used for simulation and trajectory prediction, they are not in a form that reveals physical meaning. For an interpretable model, we require domain knowledge. Please see Supplementary Discussion 3 for another example on canonical dictionary functions.

**Example 11: Non-hybrid Dynamical Systems**

We also tested IHYDE on non-hybrid dynamical systems using datasets in [13] to illustrate the applicability of IHYDE. The details of simulation datasets in [13] are presented in Supplementary Table 47. The results are summarized in Supplementary Table 48, and Supplementary Table 49 shows the hyperparameters tuned for IHYDE. Overall, IHYDE unifies previous results for the discovery of non-hybrid dynamical systems, such as examples in [13, 14].

**Supplementary Note 4: User's Manual of the Code**

Identification of hybrid dynamical systems (IHYDE) is a open-source Matlab toolbox for automating the mechanistic modeling of hybrid dynamical systems from observed data. IHYDE has low computational complexity, enabling its application to real-world CPS problems. IHYDE implements the clustering-based algorithms described in the Data-driven Discovery of Cyber Physical Systems. It can also be used, potentially, for the creation of guidelines for designing new CPSs. IHYDE uses routines of the CVX [15] and SLR [16] toolboxes for constructing and solving disciplined convex programs (DCPs).

Download the latest version of IHYDE toolbox in a directory and add its path (and the path of the subdirectories) to the Matlab path. The IHYDE toolbox consists of directories listed in Supplementary Table 51. Supplementary Table 52, Supplementary Table 53, Supplementary Table 54 and Supplementary Table 55 give a brief introduction to IHYDE's API.

To quickly get familiar with IHYDE, examples are presented in the directory /CPSid. These .m files can also be used as templates for other experiments. We shall use the autonomous car example to explain the code briefly. First, we load the data:

```
addpath('./tools');
addpath('./data');
basis_function.work='off';
data=load('normal_car.mat');  %% load Data
index = 1000:1400;
flag = data.flag(index);    % 1:straghtway    0:curve
dy = data.dy(index);
v = data.v(index)/10;
```

```
polyorder = 4; % The highest order of the polynomial is 4 order
A= library(v,polyorder,memory,basis_function);%make a library
A = A(memory+2:end,:);
dy = dy(memory+2:end); %dpwm_{k}
flag = flag(memory+2:end); %flag_{k}
v_k1 = v(memory+1:end-1,:);   % v_{k-1}
v_k2 = v(memory:end-2,:);% v_{k-2}
v_k3 = v(memory-1:end-3,:);% v_{k-3}
v = v(memory+2:end,:);   % v_{k}
```

Then, we initialize the parameters and identify the systems by function `ihyde`.

```
parameter.MAXITER = 5;    %the iter for the sparsesolver algorithm
parameter.max_s = 20;              % the max number of subsystems
parameter.epsilon = [100  8];   %  the fist element in lambda is
    epsilon_z and the second is epsilon_w
parameter.Phi = A;      % the library
parameter.y = dy;   % dpwm
parameter.normalize_y = 1;  % normalize: 1; unnormalize:0
[result]=ihyde(parameter);        % inferring subsystems
```

Function `ihyde` will return a preliminary identified result which contains the details of subsystems. Since we want to get a better result based on the minimum error principle, we use function `finetuning` to fine-tune the results.

```
result.epsilon = parameter.epsilon(2);% use epsilon_w as the
```

```
    epsilon in finetuning
result.lambda = parameter.lambda(2);% use lambda_w as the epsilon
    in finetuning
result.threshold = [0.05];     %set a threshold for clustering
final_result  = finetuning(result);   % finetuning each subsystem
sys = final_result.sys;  % get the identified subsystems
idx_sys = final_result.idx;% get the index of each subsystem
```

The code for inferring transition logics between subsystems is shown below.

```
Phi2 = [ones(size(flag)) flag 1./v sin(v) cos(v) v.^2  v_k1./v_k2
    v_k3.^2 ];% library for inferring transition logic between
    subsystems.
para_log.idx_sys = idx_sys;
para_log.beta= 0.5;    % the tradeoff of l1-sparse logistic
    regression
para_log.y = dy;
para_log.Phi2 = Phi2;
[syslogic,labelMat,data] = ihydelogic(para_log);
```

The identified results are saved in `sys`, `idx_sys` and `syslogic`.

## SUPPLEMENTARY DISCUSSIONS

The IHYDE algorithm has been tested in a number of examples. As the number of dictionary functions and the amount of noise increase, the algorithm is eventually unable to identify the actual model. Although it can fit data very well, it usually obtains more complex models than the true ones. This is actually a typical problem in system identification [2]. When the data is not informative, it leads to non-identifiability issues, i.e., there will exist multiple hybrid dynamical systems that can produce the same data, which prevents the proposed IHYDE algorithm from finding the true system.

**Supplementary Discussion 1: Identifiability**

Consider the following linear system with unknown parameters $k_1$ and $k_2$

$$\begin{aligned} \frac{d}{dt}\begin{bmatrix} x_1 \\ x_2 \end{bmatrix} &= \begin{bmatrix} k_1 & 1+k_2 \\ 0 & k_1+k_2 \end{bmatrix}\begin{bmatrix} x_1 \\ x_2 \end{bmatrix} + \begin{bmatrix} 0 \\ 1 \end{bmatrix} u, \\ y &= \begin{bmatrix} 0 & 1 \end{bmatrix}\begin{bmatrix} x_1 \\ x_2 \end{bmatrix}. \end{aligned} \tag{19}$$

The observed output is plotted as follows in Supplementary Figure 15 (the system is stimulated by an impulse input, i.e., $u(t) = \delta(t)$ where $\delta(\cdot)$ is the Dirac delta function):

However, any $k_1, k_2$ with $k_1+k_2 = 0.8$ produces the same input-output data. For example, the actual system

$$\begin{aligned} \frac{d}{dt}\begin{bmatrix} x_1 \\ x_2 \end{bmatrix} &= \begin{bmatrix} 0.4 & 1.4 \\ 0 & 0.8 \end{bmatrix}\begin{bmatrix} x_1 \\ x_2 \end{bmatrix} + \begin{bmatrix} 0 \\ 1 \end{bmatrix} u, \\ y &= \begin{bmatrix} 0 & 1 \end{bmatrix}\begin{bmatrix} x_1 \\ x_2 \end{bmatrix}, \end{aligned} \tag{20}$$

and

$$\begin{aligned} \frac{d}{dt}\begin{bmatrix} x_1 \\ x_2 \end{bmatrix} &= \begin{bmatrix} 0.3 & 1.5 \\ 0 & 0.8 \end{bmatrix}\begin{bmatrix} x_1 \\ x_2 \end{bmatrix} + \begin{bmatrix} 0 \\ 1 \end{bmatrix} u, \\ y &= \begin{bmatrix} 0 & 1 \end{bmatrix}\begin{bmatrix} x_1 \\ x_2 \end{bmatrix}, \end{aligned} \tag{21}$$

are indistinguishable from the input-output data alone. Hence, without more information, the true parameters cannot be identified using any methods.

**Supplementary Discussion 2: Data Informativity**

The previous example demonstrates that, when the parameterization is not identifiable, no algorithm is able to identify the correct parameters. Next, we shall demonstrate another example where, even though the subsystem is identifiable, the data is not informative

enough. For example, some of the logic transitions never occur. Consider the following hybrid dynamical system in Supplementary Figure 16. If the system starts at initial condition $y(0) = 18$, then it always stays in subsystem 1. Hence, with the data generated, no algorithm is able to identify the complete hybrid dynamical system.

### Supplementary Discussion 3: Canonical dictionary functions

With an example, this section explores the effect of dictionary functions when the right choice of dictionary functions is unclear and/or domain knowledge is lacking. Consider a hybrid dynamical system with two subsystems: subsystem 1 follows $\dot{x} = -x^3$, and subsystem 2 with $\dot{x} = -\cos(x)$. This hybrid system switches every $0.5s$ during $t \in [0, 10]$. We set the initial condition to $x_0 = 0.99$ and the sampling period to $0.005s$. Then, 2000 simulated data points are obtained. We choose the first 1000 points as training data set, denoted by $\mathcal{I}_{\text{train}} = \{1, \cdots, 1000\}$, and the whole data as testing data set. Assume there is no prior knowledge about the function forms of the subsystems. Then, pick a canonical dictionary function consisting of polynomials up to fifth order, grid the hyperparameters using the initial grid set in Supplementary Method 1, and use the minimum error principle to search a best set of hyperparameters.

Supplementary Table 50 summarizes the identified results. IHYDE first correctly discovers one of the subsystems $\dot{x} = -x^3$ and then discovers a second subsystem with the form $\dot{x} = -1 + \frac{1}{2}x^2$, which is different from the true subsystem. On the other hand, this is consistent with the Taylor series expansion of $\cos(x) = 1 - \frac{1}{2}x^2 + O(x^4)$.

## SUPPLEMENTARY METHODS

### Supplementary Method 1: IHYDE Algorithm

When a hybrid dynamical system has a single subsystem, i.e., $K = 1$ in Eq. (1), it becomes a time-invariant nonlinear dynamical system. We start by briefly reviewing identification tools for this class of systems from [13, 14], since parts of our proposed algorithm are based on these tools. As explained before, our algorithm uses only time-series data to directly model the system. Hence, the first step is to collect time-course input-output data $(\mathbf{y}(t), \mathbf{u}(t))$

uniformly sampled at a number of discrete time indices $t = 1, 2, \ldots, M+1$. Let

$$\mathbf{Y} = \begin{bmatrix} | & | & & | \\ \mathbf{y}(1) & \mathbf{y}(2) & \ldots & \mathbf{y}(M) \\ | & | & & | \end{bmatrix}^T, \quad \mathbf{U} = \begin{bmatrix} | & | & & | \\ \mathbf{u}(1) & \mathbf{u}(2) & \ldots & \mathbf{u}(M) \\ | & | & & | \end{bmatrix}^T.$$

Note that $\mathbf{y}(t) \in \mathbb{R}^n$ and $\mathbf{u}(t) \in \mathbb{R}^m$, and so $\mathbf{Y} \in \mathbb{R}^{M\times n}$ and $\mathbf{U} \in \mathbb{R}^{M\times m}$. Next, we construct an overdetermined library $\mathbf{\Phi}(\mathbf{Y}, \mathbf{U})$ consisting of potential nonlinear functions that appear in $\mathbf{f}_k$ in Eq. (1). It is expected that the true nonlinearities are part of this library in order to recover the true dynamics. The choice of these functions is guided by the particular field of study. For example, the library would consist of sinusoidal functions in pendulums, and polynomial and sigmoidal functions in biochemical networks. As an illustration, a library consisting of constant or polynomials would result in the following dictionary matrix

$$\mathbf{\Phi}(\mathbf{Y}, \mathbf{U}) = \begin{bmatrix} \mathbf{1} & \mathbf{Y} & \mathbf{Y}^{P_2} & \cdots & \mathbf{U} & \mathbf{U}^{P_2} & \cdots \end{bmatrix}.$$

Here, higher polynomials are denoted as $\mathbf{Y}^{P_2}, \mathbf{Y}^{P_3}$, etc. For example, $\mathbf{Y}^{P_2}$ denotes the quadratic nonlinearities in the state variable $Y$, given by:

$$\mathbf{Y}^{P_2} = \begin{bmatrix} y_1^2(1) & y_1(1)y_2(1) & \cdots & y_n^2(1) \\ y_1^2(2) & y_1(2)y_2(2) & \cdots & y_n^2(2) \\ \vdots & \vdots & \ddots & \vdots \\ y_1^2(M) & y_1(M)y_2(M) & \cdots & y_n^2(M) \end{bmatrix}.$$

Basically, each column of $\mathbf{\Phi}(\mathbf{Y}, \mathbf{U})$ represents a candidate function for a nonlinearity in $\mathbf{f}$. The number of functions in the library may be very large. However, since only a very small number of these nonlinearities appear in each row of $\mathbf{\Phi}(\mathbf{Y}, \mathbf{U})$, we can set up a sparse regression problem to determine the sparse matrices of coefficients $\mathbf{W} = \begin{bmatrix} \mathbf{w}_1 & \mathbf{w}_2 & \ldots & \mathbf{w}_n \end{bmatrix}$, where $\mathbf{w}_i \in \mathbb{R}^{P\times 1}$ and $P$ is the total number of candidate functions in the library. The nonzero elements in $\mathbf{W}$ determine which nonlinearities are active [13, 14] and the corresponding parameters. Let

$$\bar{\mathbf{Y}} \triangleq \begin{bmatrix} y_1(2) & \ldots & y_n(2) \\ y_1(3) & \ldots & y_n(3) \\ \vdots & \ddots & \vdots \\ y_1(M+1) & \ldots & y_n(M+1) \end{bmatrix}.$$

This results in the overall model $\bar{\mathbf{Y}} = \mathbf{\Phi}(\mathbf{Y},\mathbf{U})\mathbf{W} + \Xi$, where $\Xi = \begin{bmatrix} \boldsymbol{\xi}_1 & \boldsymbol{\xi}_2 & \dots & \boldsymbol{\xi}_n \end{bmatrix}$ and $\boldsymbol{\xi}_i \in \mathbb{R}^{M\times 1}$ is zero-mean i.i.d. Gaussian noise with covariance matrix $\lambda\mathbf{I}$, for some $\lambda \geq 0$. The work in [14], developed methods based on Sparse Bayesian Learning for identifying each $\mathbf{w}_i$ in the above equation as the following optimization:

$$\mathbf{w}_i^* = \arg\min_{\mathbf{w}_i} \|\bar{\mathbf{y}}_i - \mathbf{\Phi}\mathbf{w}_i\|_{\ell_2}^2 + \lambda\|\mathbf{w}_i\|_{\ell_1}. \tag{22}$$

**Inferring Sub-systems**

When $K > 1$, we can use a similar formulation as above. However, the outstanding challenge is that there is no single $\mathbf{W}$ typically fits all the data due to the hybrid nature of the dynamical system. In addition, we have no information about which data point belongs to which subsystem. Next, we introduce a new method to tackle such a challenge.

Define $\mathbf{Z} = \bar{\mathbf{Y}} - \mathbf{\Phi}\mathbf{W} - \Xi$. The goal is to find a $\mathbf{Z}^* \triangleq \begin{bmatrix} \mathbf{z}_1^* & \mathbf{z}_2^* & \dots & \mathbf{z}_n^* \end{bmatrix} \triangleq \bar{\mathbf{Y}} - \mathbf{\Phi}\mathbf{W}^* - \Xi$ as sparse as possible, i.e.,

$$\begin{gathered} \mathbf{W}^* = \arg\min_{\mathbf{W}} \sum_{i=1}^{n} \|\mathbf{z}_i\|_{\ell_0}, \\ \text{subject to: } \mathbf{Z} = \bar{\mathbf{Y}} - \mathbf{\Phi}\mathbf{W} - \Xi. \end{gathered} \tag{23}$$

Correspondingly, we have $\mathbf{z}_i^* = \bar{\mathbf{y}}_i - \mathbf{\Phi}\mathbf{w}_i^* - \boldsymbol{\xi}_i$, where $\bar{\mathbf{y}}_i$ is the ith column of $\bar{\mathbf{Y}}$. The interpretation of this optimization is to find a $\mathbf{W}$ (or equivalently a subsystem) that fits most of the input-output data. As a result, the indexes of the zero entries of $\mathbf{Z}^*$ correspond to the indexes for input-output that can be fitted by a single subsystem. This initial idea was similar to those presented in [17] for noiseless switching subsystem identification, yet we now extend this idea to a robust Bayesian algorithm that works well for noisy data (for detailed comparison, please refer to the following part: Supplementary Comparison). To solve Eq. (23), assume, without loss of generality, that the dictionary matrix $\mathbf{\Phi}$ is full rank. Define a transformation matrix $\mathbf{\Theta} \in \mathbb{R}^{(M-P)\times M}$ whose rows $\{\mathbf{\Theta}[1,:], \dots, \mathbf{\Theta}[M-P,:]\}$ form a basis for the left null space of $\mathbf{\Phi}$. Then, it follows that $\mathbf{\Theta}\bar{\mathbf{Y}} = \mathbf{\Theta}\mathbf{Z} + \mathbf{\Theta}\Xi$. Using standard maximum likelihood estimate and an appropriate Lagrange multiplier $\frac{1}{2\lambda_{\mathbf{z}}}$, we now can rewrite the above problem as an unconstrained minimization:

$$\min_{\mathbf{Z}} \frac{1}{2} \left\| (\tilde{\bar{\mathbf{Y}}} - \mathbf{\Theta}\mathbf{Z})^T \mathbf{\Pi}^{-1} (\tilde{\bar{\mathbf{Y}}} - \mathbf{\Theta}\mathbf{Z}) \right\|_F^2 + \lambda_{\mathbf{z}} \sum_{i=1}^{n} \|\mathbf{z}_i\|_{\ell_0}, \tag{24}$$

where $\tilde{\bar{\mathbf{Y}}} \triangleq \mathbf{\Theta}\bar{\mathbf{Y}}$ and $\mathbf{\Pi} = \mathbf{\Theta}\mathbf{\Theta}^T$.

**Remark 1** *This is the key step in the later proposed algorithm; there is no* $\mathbf{W}$ *in this optimization after the transformation. Instead, we are optimizing over the residual* $\mathbf{Z}$.

However, this problem is known to be computationally expensive. Instead, we use the following convex relaxation

$$\mathbf{Z}^* = \arg\min_{\mathbf{Z}} \frac{1}{2} \left\| (\tilde{\bar{\mathbf{Y}}} - \mathbf{\Theta}\mathbf{Z})^T \mathbf{\Pi}^{-1} (\tilde{\bar{\mathbf{Y}}} - \mathbf{\Theta}\mathbf{Z}) \right\|_F^2 + \lambda_{\mathbf{z}} \sum_{i=1}^{n} \|\mathbf{z}_i\|_{\ell_1}.$$

We can decompose the above optimization to a number of smaller optimizations: for $i = 1, \ldots, n$

$$\mathbf{z}_i^* = \arg\min_{\mathbf{z}_i} \frac{1}{2} (\tilde{\bar{\mathbf{y}}}_i - \mathbf{\Theta}\mathbf{z}_i)^T \mathbf{\Pi}^{-1} (\tilde{\bar{\mathbf{y}}}_i - \mathbf{\Theta}\mathbf{z}_i) + \lambda_{\mathbf{z}} \|\mathbf{z}_i\|_{\ell_1}. \tag{25}$$

**Remark 2** *Specifically, we used a Bayesian formulation to replace the optimizations in Eq.* (25) *to achieve better empirical performance as detailed in the main text.*

Once this problem is solved, we consider the index set $\mathcal{I} = \{j | \, |\mathbf{z}_i^*[j]| \le \epsilon_{\mathbf{z}}\}$ and further identify the sparse coefficients $\mathbf{w}_i^*$ using the following optimization

$$\mathbf{w}_i^* = \arg\min_{\mathbf{w}_i} \frac{1}{2} \|\bar{\mathbf{Y}}[\mathcal{I}, i] - \mathbf{\Phi}[\mathcal{I}, :]\mathbf{w}_i\|_{\ell_2}^2 + \lambda_{\mathbf{w}} \|\mathbf{w}_i\|_{\ell_1}.$$

The variables $\mathbf{w}_i^*$ are the coefficients of the identified subsystem.

**Remark 3** *The reason to enforce* $\mathbf{w}_i^*$ *to be sparse is due to the constructed dictionary matrix* $\mathbf{\Phi}$ *usually has extra terms that are not in the true dynamics.*

We further define error $= \text{abs}(\bar{\mathbf{y}}_i - \mathbf{\Phi}\mathbf{w}_i^*)$ (here abs is an elementary-wise operator which returns the absolute value of every element of a vector) and we set the $j$th element of $\bar{\mathbf{y}}_i$: $\bar{\mathbf{Y}}[j, i] = 0$ and the $j$th row of $\mathbf{\Theta}$: $\mathbf{\Theta}[j, :] = \mathbf{0}$ if the $j$th element of error is less than $\epsilon_{\mathbf{w}}$, for some small $\epsilon_{\mathbf{w}} > 0$. This removes the data that has already been fitted by the subsystem. Once we have the new $\bar{\mathbf{Y}}$ and $\mathbf{\Theta}$, we can solve the same problem with the remaining time points (where the corresponding elements of $\bar{\mathbf{Y}}$ and the corresponding row of $\mathbf{\Theta}$ are nonzero) using the exact same procedure. The number of iterations gives the minimum number of subsystems. The proposed algorithm is summarized in Supplementary Algorithm 2. The code implementation is available at `https://github.com/HAIRLAB/CPSid` with Supplementary Note 4, User's Manual. In what follows, we shall briefly discuss extensions and variants of Supplementary Algorithm 2, which can empirically improve the performance of IHYDE.

**Remark 4** *When there is only one subsystem, we show that* $\mathbf{Z}^i$ *should be a zero matrix from the first optimization in Eq.* (25)*. Eq.* (26) *should be the same as Eq.* (22) *since* $\mathcal{I} = \{1, 2, \ldots, M+1\}$*, which recovers the results for time-invariant nonlinear system identification in [13, 14]. As a result, IHYDE provides a unified point of view to the subsystem identification problem for any* $K \in \{1, 2, \ldots\}$.

**Algorithm 2** Sub-systems Identification Algorithm

**Input:** Collect input-output data $\mathbf{u}(t)$ and $\mathbf{y}(t)$ for $t = 1, 2, \ldots, M + 1$. Two pre-specified thresholds $\epsilon_{\mathbf{z}}$ and $\epsilon_{\mathbf{w}}$, two tuning parameters $\lambda_{\mathbf{z}}$ and $\lambda_{\mathbf{w}}$, the upper bound of the number of subsystems $K_{\max}$

**Output:** Return $\{\mathbf{W}^i\}$ for $i = 1, \ldots, K$ and the number of subsystems $K$

Construct dictionary matrix $\mathbf{\Phi}(\mathbf{Y}, \mathbf{U})$ based on prior knowledge of the system

**for** $j = 1, \ldots, n$ **do**

  **for** $i = 1, \ldots, K_{\max}$ **do**

    Compute $\mathbf{\Theta}$ in which all column span the left null space of $\mathbf{\Theta}$: $\mathbf{\Theta\Phi} = \mathbf{0}$

    Solve for $\mathbf{z}_j^i$ from Algorithm 1

    **if** $\mathbf{z}_j^i = \mathbf{0}$ **then**

      $K = i$, Break

    **end if**

    $h = 1$ and $\mathcal{I} = [\;]$

    **for** $l = 1 \ldots, M$ **do**

      **if** the $l$th element of $\mathbf{z}_j^i$, i.e., $\text{abs}(\mathbf{z}_j^i[l]) \le \epsilon_{\mathbf{z}}$ **then**

        Set $\mathcal{I}[h] = l$ and $h \rightarrow h + 1$

      **end if**

    **end for**

    Solve the following convex optimization

$$\mathbf{w}_j^i = \arg\min_{\mathbf{w}_j} \frac{1}{2} \|\bar{\mathbf{Y}}[\mathcal{I}, j] - \mathbf{\Phi}[\mathcal{I}, :]\mathbf{w}_j\|_{\ell_2}^2 + \lambda_{\mathbf{w}} \|\mathbf{w}_j\|_{\ell_1} \tag{26}$$

    $\text{error} = \text{abs}(\bar{\mathbf{Y}}[:, j] - \mathbf{\Phi}\mathbf{w}_j^i)$

    **for** $l = 1 \ldots, M$ **do**

      **if** the $l$th element of error, i.e., $\text{error}[l] \le \epsilon_{\mathbf{w}}$ **then**

        Set $\bar{\mathbf{Y}}[l, j] = 0$ and $\mathbf{\Theta}[l, :] = 0$

      **end if**

    **end for**

  **end for**

**end for**

Return nonzero $\mathbf{W}^i \triangleq \begin{bmatrix} \mathbf{w}_1^i & \ldots & \mathbf{w}_n^i \end{bmatrix}$ for $i = 1, \ldots, K$ and the number of subsystems $K$

*Comparison to [17] in the subsystems identification procedure*

Bako proposed a nice algorithm that novelly uses sparsity for identifying switching systems [17]. A general framework is proposed for noiseless setting and Section 3.4 of [17] suggests two ways to deal with the identification of subsystems with noisy data. The first method in [17] sets up an upper bound for the noise, and therefore this method is not practical for Gaussian noise as we considered in this paper. The second method in [17] formulates an optimization problem that tradeoffs the residual (mismatch between data and prediction from the model) and the energy of the noise. Next, we will show that the second method could be viewed as a special case of our framework. What is more, our method includes several iterations that considerably improves the results, as can be seen in the numerical examples below.

The identification of switching linear systems can be formulated as:

$$\bar{\mathbf{y}}_i = \mathbf{\Phi}\mathbf{w}_i + \mathbf{z}_i + \boldsymbol{\xi}_i, \quad i = 1, \cdots, n, \tag{27}$$

where each column of $\mathbf{\Phi} \in \mathbb{R}^{M \times P}$ is a candidate function. Note that residual $\mathbf{z}_i$ is sparse, and $\boldsymbol{\xi}_i$ is Gaussian noise. Reference [17] searches the subsystems as follows:

$$\min_{\mathbf{z}_i, \boldsymbol{\xi}_i} \lambda \|\mathbf{z}_i\|_{\ell_1} + \frac{1}{2}\|\boldsymbol{\xi}_i\|_{\ell_2}^2. \tag{28}$$

Next, we show that, since the objective function is convex with respect to $\mathbf{w}_i$, it yields the following form (where $\mathbf{\Phi}^+$ is the pseudo inverse of $\mathbf{\Phi}$)

$$\min_{\mathbf{z}_i, \mathbf{w}_i} \lambda \|\mathbf{z}_i\|_{\ell_1} + \frac{1}{2}\|\bar{\mathbf{y}}_i - \mathbf{\Phi}\mathbf{w}_i - \mathbf{z}_i\|_{\ell_2}^2 \Longleftrightarrow \min_{\mathbf{z}_i} \lambda \|\mathbf{z}_i\|_{\ell_1} + \frac{1}{2}\|(\mathbf{I} - \mathbf{\Phi}\mathbf{\Phi}^+)(\bar{\mathbf{y}}_i - \mathbf{z}_i)\|_{\ell_2}^2. \tag{29}$$

Let $\mathbf{Q} \triangleq \mathbf{I} - \mathbf{\Phi}\mathbf{\Phi}^+$, and $rank(\mathbf{\Phi}) = k \, (k \leq \min(M, P))$. Using singular value decomposition, $\mathbf{\Phi}$ can be written as follows:

$$\mathbf{\Phi} = \mathbf{A}\mathbf{S}\mathbf{V}^T = \begin{bmatrix} \mathbf{A}_{1\, M \times k} & \mathbf{A}_{2\, M \times (M-k)} \end{bmatrix} \begin{bmatrix} \mathbf{S}_{1\, k \times k} & 0 \\ 0 & 0 \end{bmatrix} \begin{bmatrix} \mathbf{V}_{1\, k \times P}^T \\ \mathbf{V}_{2\, (P-k) \times P}^T \end{bmatrix} = \mathbf{A}_1\mathbf{S}_1\mathbf{V}_1^T.$$

Therefore, the explicit form of $\mathbf{\Phi}^+$ is $\mathbf{\Phi}^+ = \mathbf{V}_1\mathbf{S}_1^{-1}\mathbf{A}_1^T$. Since $\mathbf{A}$ and $\mathbf{V}$ are unitary matrices, one has

$$\mathbf{Q} = \mathbf{I}_M - \mathbf{A}_1\mathbf{S}_1\mathbf{V}_1^T\mathbf{V}_1\mathbf{S}_1^{-1}\mathbf{A}_1^T = \mathbf{A}_2\mathbf{A}_2^T.$$

**Remark 5** *Rather than having the derivation we had above, [17] gives the orthogonal projection matrix as,* $\mathbf{Q} = \mathbf{I}_M - \mathbf{\Phi}(\mathbf{\Phi}^T\mathbf{\Phi})^{-1}\mathbf{\Phi}^T$. *Note that it is only true by assuming that* $\mathbf{\Phi}$ *has full rank (which is usually not the case in our numerical examples).*

One can rewrite Eq. (29) as,

$$\min_{\mathbf{z}_i} \lambda\|\mathbf{z}_i\|_{\ell_1} + \frac{1}{2}\|\mathbf{Q}(\bar{\mathbf{y}}_i - \mathbf{z}_i)\|_{\ell_2}^2 \Longleftrightarrow \min_{\mathbf{z}_i} \lambda\|\mathbf{z}_i\|_{\ell_1} + \frac{1}{2}(\bar{\mathbf{y}}_i - \mathbf{z}_i)^T\mathbf{A}_2\mathbf{A}_2^T(\bar{\mathbf{y}}_i - \mathbf{z}_i).$$

In contrast, IHYDE is based on Bayesian calculus. As stated before that the transformation matrix $\mathbf{\Theta}$ is the orthogonal left null space of matrix $\mathbf{\Phi}$, namely, $\mathbf{\Theta\Phi} = \mathbf{0}$. Left multiply Eq. (27) by matrix $\mathbf{\Theta}$ gives

$$\mathbf{\Theta}\bar{\mathbf{y}}_i \triangleq \tilde{\bar{\mathbf{y}}}_i = \mathbf{\Theta}\mathbf{z}_i + \mathbf{\Theta}\boldsymbol{\xi}_i.$$

To get an estimate of $\mathbf{z}_i$, we use Bayesian modeling to treat all unknowns as stochastic variables with certain probability distributions [18]. Given the characteristics of the noise $\boldsymbol{\xi}_i$, $\mathbf{\Theta}\boldsymbol{\xi}_i$ is Gaussian distributed with covariance matrix $\lambda\mathbf{\Theta}\mathbf{\Theta}^T$, i.e., $\mathbf{\Theta}\boldsymbol{\xi}_i \sim \mathcal{N}(\mathbf{0}, \lambda\mathbf{\Theta}\mathbf{\Theta}^T)$. In such a case, using the properties of Gaussian distributions, the likelihood of the output $\tilde{\bar{\mathbf{y}}}_i$ given the parameter $\mathbf{z}_i$ is

$$p(\tilde{\bar{\mathbf{y}}}_i|\mathbf{z}_i) = \mathcal{N}(\tilde{\bar{\mathbf{y}}}_i|\mathbf{\Theta}\mathbf{z}_i, \lambda\mathbf{\Theta}\mathbf{\Theta}^T) \propto \exp\left[-\frac{1}{2\lambda}(\tilde{\bar{\mathbf{y}}}_i - \mathbf{\Theta}\mathbf{z}_i)^T(\mathbf{\Theta}\mathbf{\Theta}^T)^{-1}(\tilde{\bar{\mathbf{y}}}_i - \mathbf{\Theta}\mathbf{z}_i)\right]. \quad (30)$$

Hence, using maximum likelihood estimation, we have the following optimization

$$\mathbf{z}_i = \arg\min_{\mathbf{z}_i}(\tilde{\bar{\mathbf{y}}}_i - \mathbf{z}_i)^T\mathbf{\Theta}^T(\mathbf{\Theta}\mathbf{\Theta}^T)^{-1}\mathbf{\Theta}(\tilde{\bar{\mathbf{y}}}_i - \mathbf{z}_i). \quad (31)$$

Next, we introduce sparse priors [19]. In Bayesian models, a prior density $p(\mathbf{z}_i)$ is defined as $p(\mathbf{z}_i) = \prod_{j=1}^{M} p(\mathbf{Z}[j,i])$. Then, we can look at the first iteration, which yields

$$\mathbf{z}_i = \arg\min_{\mathbf{z}_i} \lambda\|\mathbf{z}_i\|_{\ell_1} + \frac{1}{2}(\tilde{\bar{\mathbf{y}}}_i - \mathbf{z}_i)^T\mathbf{\Theta}^T(\mathbf{\Theta}\mathbf{\Theta}^T)^{-1}\mathbf{\Theta}(\tilde{\bar{\mathbf{y}}}_i - \mathbf{z}_i). \quad (32)$$

One can get the orthogonal left null space matrix $\mathbf{\Theta} = \mathbf{A}_2^T$ using singular value decomposition. Therefore, Eq. (32) can be rewritten as,

$$\begin{aligned} &\min_{\mathbf{z}_i} \lambda\|\mathbf{z}_i\|_{\ell_1} + \frac{1}{2}(\tilde{\bar{\mathbf{y}}}_i - \mathbf{z}_i)^T\mathbf{A}_2(\mathbf{A}_2^T\mathbf{A}_2)^{-1}\mathbf{A}_2^T(\tilde{\bar{\mathbf{y}}}_i - \mathbf{z}_i) \\ \Longleftrightarrow &\min_{\mathbf{z}_i} \lambda\|\mathbf{z}_i\|_{\ell_1} + \frac{1}{2}(\tilde{\bar{\mathbf{y}}}_i - \mathbf{z}_i)^T\mathbf{A}_2\mathbf{A}_2^T(\tilde{\bar{\mathbf{y}}}_i - \mathbf{z}_i). \quad (33) \end{aligned}$$

Note that our method includes the methods in [17] as special cases by setting the number of iterations to 1.

Next, we illustrate the performance differences between the second method in [17] and the IHYDE on the Continuous Hysteresis Loop data, with 6% noise. For both methods,

500 samples are used for training. Our method consists of an iterative algorithm; we are going to compare two scenarios. First, we set the number of iterations in our method to 1, and carefully tune the hyperparameters for both algorithms in order to make a fair comparison. Second, we set the number of iterations to 5 (which is a default setting for IHYDE). Supplementary Table 1 shows the parameters and summarizes the identification results.

It can be seen that both method with the number of iterations set to 1 efficiently distinguish the number of subsystems. In theory, these two methods should have equivalent performance. However, in practice, our present method identifies the sparse dynamics describing two subsystems; while the method in [17] does not. The reason for this could be Eq. (28) of [17] optimizes over two variables $\mathbf{z}_i, \boldsymbol{\xi}_i$. While our method in Eq. (32) optimizes over one variable $\mathbf{z}_i$. Since these optimizations are solved by first-order optimization methods, such as variants of gradient descent, such searches are prone to stuck in local minimum when there are a larger number of variables. More importantly, our method with 5 iterations (default setup) accurately identifies the dynamics describing two subsystems, showing an improved performance. In addition, the methods in [17] do not recover the transition rules, while IHYDE does, as shown below.

**Inferring Transition Logics**

Once the subsystems have been identified, we can assign every input-output data point $(\mathbf{u}(t), \mathbf{y}(t))$ to a specific subsystem as shown in Supplementary Figure 1. The next step is to identify the transition logics between different subsystems. We first convert the problem of identifying the transition logics to a standard sparse logistic regression problem which can be efficiently solved by many methods in the literature. The scheme is illustrated in Supplementary Figure 2.

To proceed, we define $\eta_i(t)$ as the set membership which equals to 1 only if the subsystem $i$ is active at discrete-time $t$ or otherwise it equals to 0. The goal is to identify the transition rules $\mathcal{T}_{i \to j}$ between any subsystems $i, j$. These functions are known from the information in the subsystem identification above. Define step$(x)$, which equals 1 if $x \geq 0$, and 0 otherwise. Mathematically, we are searching for a nonlinear function $g$, such that step$(g(\mathbf{y}(t), \mathbf{u}(t)))$ specifies the membership. Due to non-differentiability of step functions at 0, we alternatively

relax the step function to a sigmoid function, i.e., $\eta_j(t+1) \approx \frac{1}{1+e^{-g(\mathbf{y}(t),\mathbf{u}(t))}}$, where $j$ is a potential subsystem that we can jump to at time $t+1$. Assuming we are in subsystem $i$ at time $t$, the fitness function to jump to subsystem $j$ at time $t+1$ is then

$$\sum_{t=1}^{M} \eta_i(t) \left\| \eta_j(t+1) - \frac{1}{1+e^{-g(\mathbf{y}(t),\mathbf{u}(t))}} \right\|_{\ell_2}^2. \tag{34}$$

To solve the optimization in (34), we can parameterize $g(\mathbf{y}(t), \mathbf{u}(t))$ as a linear combination of over-determined dictionary matrix, i.e., $g(\mathbf{y}(t), \mathbf{u}(t)) \triangleq \mathbf{\Psi}(\mathbf{Y}, \mathbf{u})[t, :]\mathbf{v}$, in which $\mathbf{\Psi}$ can be constructed similarly as $\mathbf{\Phi}$ in the previous section and $v$ is a vector of to-be-discovered parameters. The cost function only takes non-zero value when $\eta_i(t) = 1$. Let $\mathcal{D} \triangleq \{t|\eta_i(t) = 1, t = 1, \cdots, M\}$, then

$$\sum_{t=1}^{M} \eta_i(t) \left\| \eta_j(t+1) - \frac{1}{1+e^{-g(\mathbf{y}(t),\mathbf{u}(t))}} \right\|_{\ell_2}^2 = \sum_{t\in\mathcal{D}} \left\| \eta_j(t+1) - \frac{1}{1+e^{-\mathbf{\Psi}[t,:]\mathbf{v}}} \right\|_{\ell_2}^2. \tag{35}$$

After this transformation, the minimization of Eq. (35) is known as the logistic regression. Hence, we can use standard gradient descent method to solve the logistic regression [20].

Similarly, we can also add an $\ell_1$ regularizer in the optimization, i.e., we minimize the following expression

$$\sum_{t\in\mathcal{D}} \left\| \eta_j(t+1) - \frac{1}{1+e^{-\mathbf{\Psi}[t,:]\mathbf{v}}} \right\|_{\ell_2}^2 + \beta\|\mathbf{v}\|_{\ell_1}, \tag{36}$$

where $\beta$ is a predefined parameter. There are many Matlab codes for sparse linear logistic regression. Here, we adopt the implementation framework proposed in [16].

---

**Algorithm 3** Transition Logics Identification Algorithm

---

1: **Input:** Input-output data $\mathbf{y}(t), \mathbf{u}(t)$ and $\eta_i(t)$, $i = 1, 2 \ldots, K$ and $t = 1, 2, \ldots, M$

2: **Output:** Transition logics $\mathcal{T}_{i\to j}(\mathbf{y}(t), \mathbf{u}(t))$ for any pair $i, j$

3: **for** $i = 1, \ldots, K$ **do**

4:     **for** $j \neq i$ **do**

5:         Construct the dictionary matrix $\mathbf{\Psi}$ from prior knowledge as described in the main text

6:         The solution to the logistic regression in Eq. (36) gives the transition model for $\mathcal{T}_{i\to j}$

7:     **end for**

8: **end for**

9: Return all transition logics mapping $\mathcal{T}$

---

**Principles for Parameter Tuning**

We tune hyperparameters based on minimum error principle described below. For a set of determined parameters $\lambda_{\mathbf{z}}, \lambda_{\mathbf{w}}, \epsilon_{\mathbf{z}}, \epsilon_{\mathbf{w}}$, we compute the fitting error for each subsystem on test data points based on an Akaike information criterion (AIC) type algorithm given by

$$\mathrm{err} = 2\mu + 2\sum_{i=1}^{n} \sqrt{\sum_{r=1}^{M} \min_{j\in\{1,\cdots,K\}} (\bar{\mathbf{Y}}[r,i] - \mathbf{\Phi}[r,:]\mathbf{w}_i^j)^2}, \tag{37}$$

where $\mu$ represents the number of non-zeros terms in all identified subsystems, and $K$ represents the number of identified subsystems. To search for hyperparameters, we empirically set an initial grid and search the optimal hyperparameters to minimize the AIC-type error. The initial grid is divided into 210 combinations as follows:

$$\begin{aligned}
\lambda_{\mathbf{z}} &\in \{10^{-7+m} | m = 1,2,3,4,5,6,7\}, \\
\lambda_{\mathbf{w}} &\in \{10^{-4+m} | m = 1,2,3\}, \\
\epsilon_{\mathbf{z}} &\in \{10^{-5+m} | m = 1,2\}, \\
\epsilon_{\mathbf{w}} &\in \{0.001m \|\bar{\mathbf{Y}}[\mathcal{I}_{\mathrm{train}}, i]\|_2 | m = 1,3,5,7,9; i = 1,\ldots,n\}.
\end{aligned}$$

Next, we detail this test strategy and illustrate it on four examples. The data contains 4000 samples with 6% noise. Of those, 500 samples, denoted by $\mathcal{I}_{\mathrm{train}} = \{1,2,\ldots,500\}$, are used for training, and all the samples, denoted by $\mathcal{I}_{\mathrm{test}} = \{1,2,\ldots,4000\}$, are used for testing. Supplementary Table 2 summarizes the optimal hyperparameters and the identified subsystems obtained from the proposed minimum error principle. These examples illustrate that this initial grid and the minimum error principle can be useful for hyper-parameter tuning. Indeed, the identified parameters of all subsystem are good approximations of the true model. Note that, in general, this initial grid can be extended until the algorithm achieves good performance with low residuals.

## SUPPLEMENTARY FIGURES

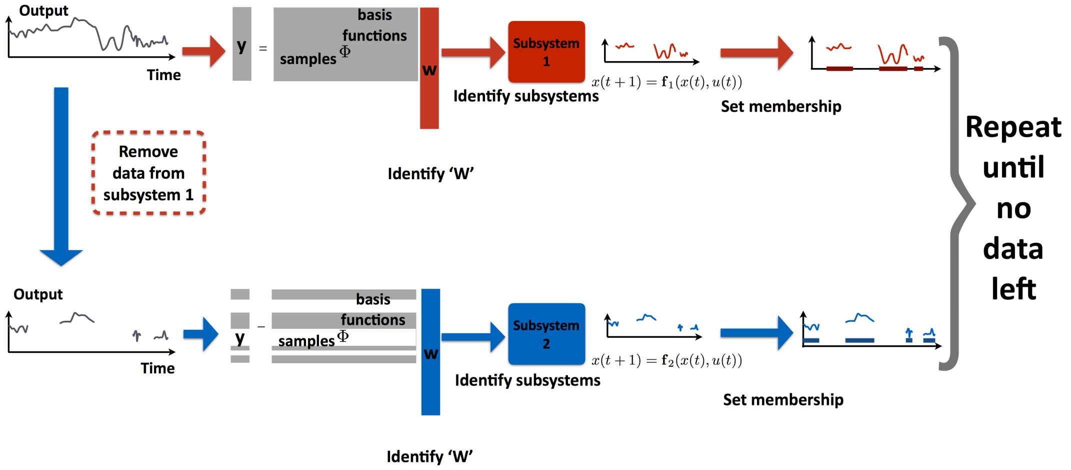


Supplementary Figure 1. Schematics of the proposed subsystems identification algorithm. We construct a library of nonlinear functions $\mathbf{\Phi}$. We formulate an iterative convex optimization method to infer the number of subsystems and the underlying system models for every subsystem. More specifically, we first identify a best model that fits the majority of data, then we remove the fitted data and re-do the identification until no data are left.

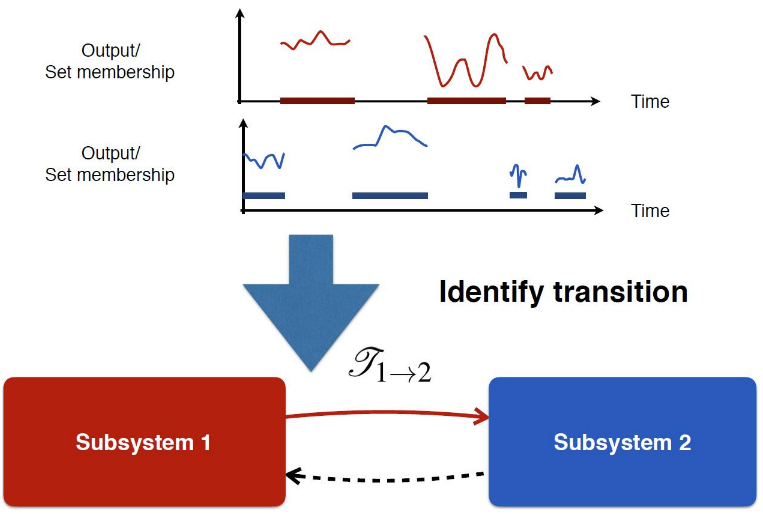


Supplementary Figure 2. Illustration of the proposed Algorithm to identify transition logics. Using the membership of every classified data point, we apply logistic regression to infer the logic between every pair of identified subsystems, i.e., $\mathcal{T}_{i \to i'}$ for every $i$ and $i'$.

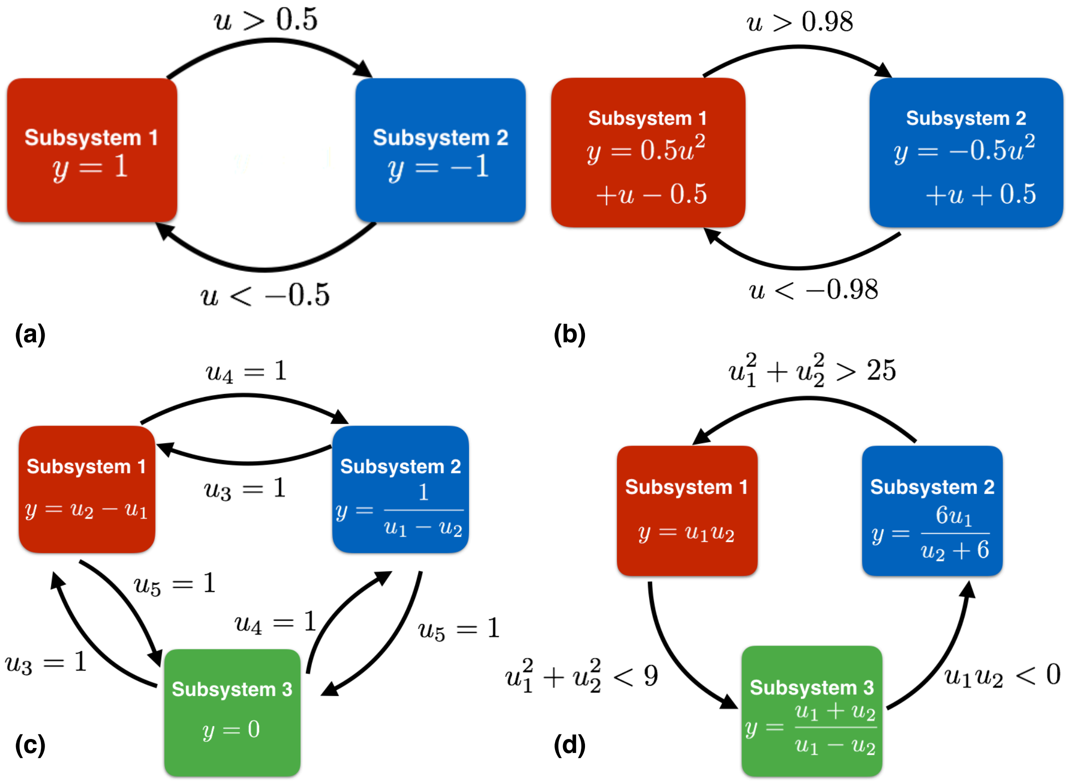


Supplementary Figure 3. The hybrid dynamical system model with the measured input-output $\boldsymbol{u}$ and $\boldsymbol{y}$. (a) Hysteresis Relay system. (b) Continuous Hysteresis Loop system. (c) Phototaxis Robot system. (d) The nonlinear hybrid dynamical system.

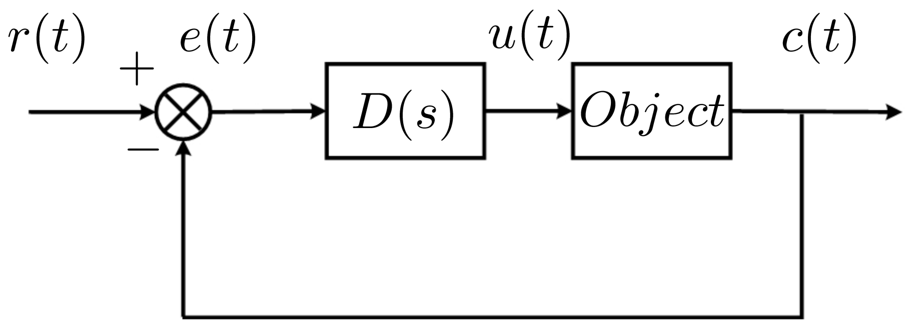


Supplementary Figure 4. The position PI controller structure for the autonomous car.

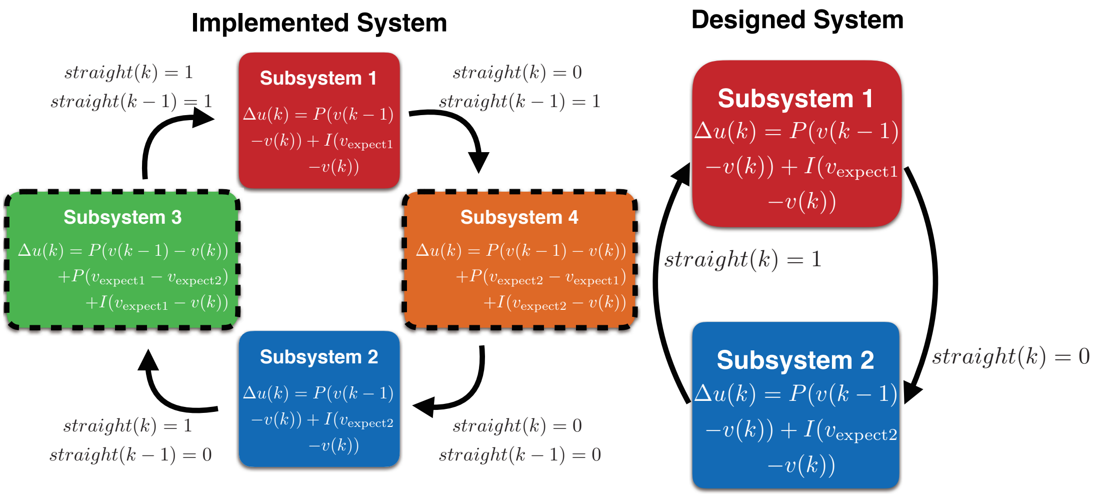


Supplementary Figure 5. Left: a more complicated hybrid dynamical system model due to discretization and switching. Right: the correct hybrid dynamical system model that we would like to design.

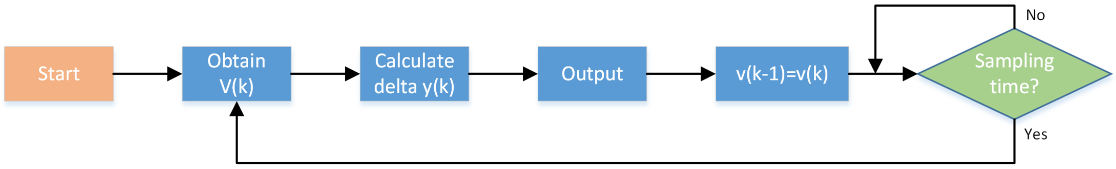


Supplementary Figure 6. The flow chart of the PI algorithm.

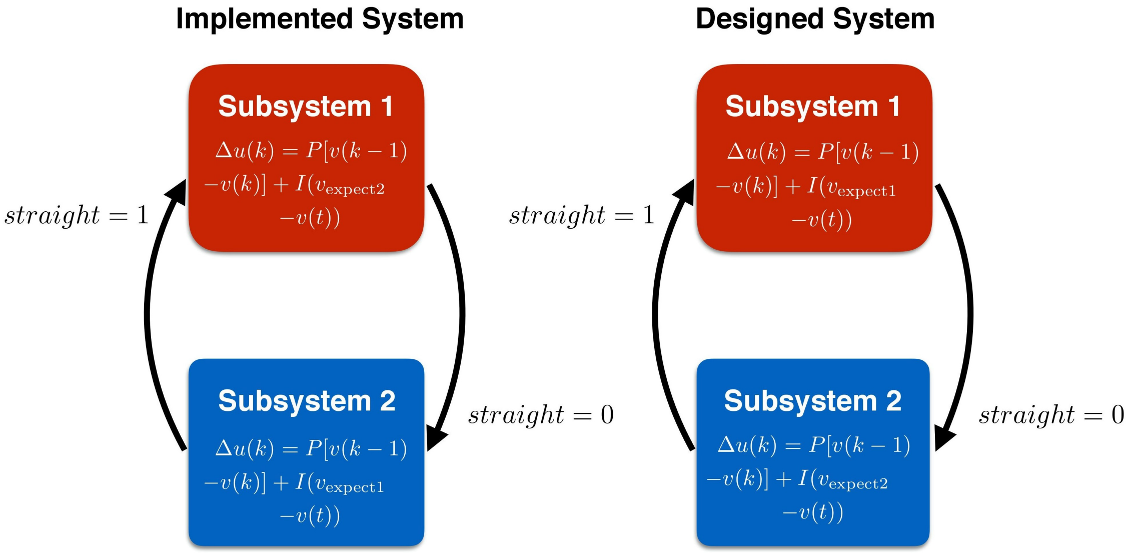


Supplementary Figure 7. The IHYDE pinpoints the implementation error that leads to a failure in the autonomous car experiment. Left: the identified model from experimental data. Right: the designed system. The two subsystems are swapped around due to a design bug.

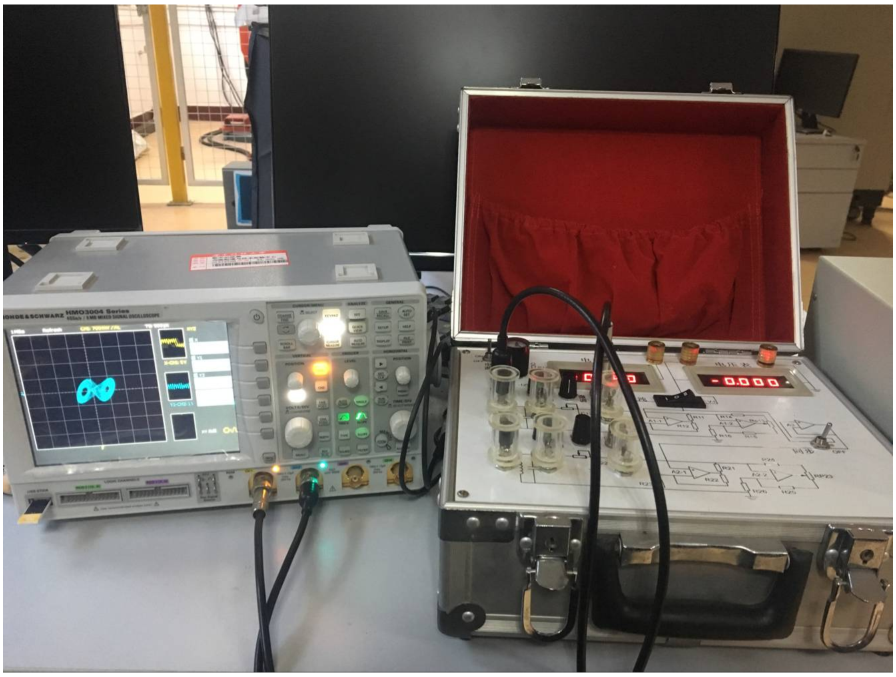

Supplementary Figure 8. The experiment platform of Chua's circuit built in the lab.

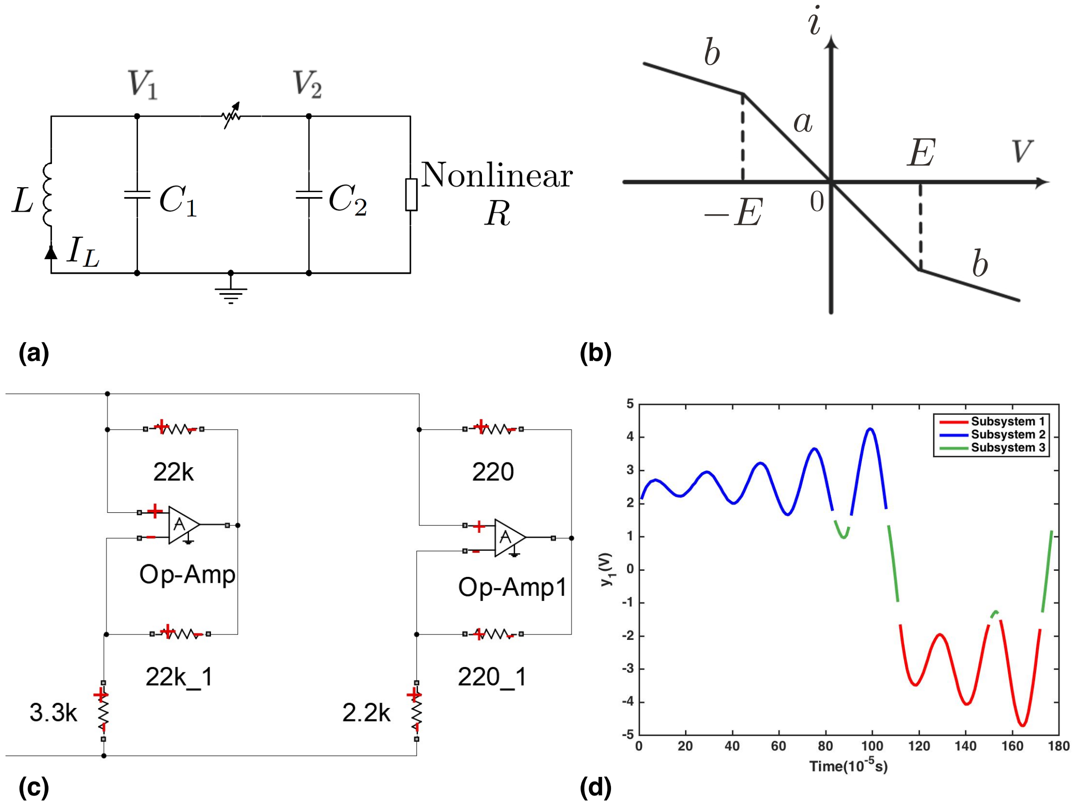


Supplementary Figure 9. Experiments of Chua's circuit. (a) the circuit structure. (b) the current-voltage characteristics of the nonlinear resistor. (c) the circuit structure of nonlinear resistor with specified current-voltage realization. (d) The output trajectory associated with different colors generated by different subsystems.

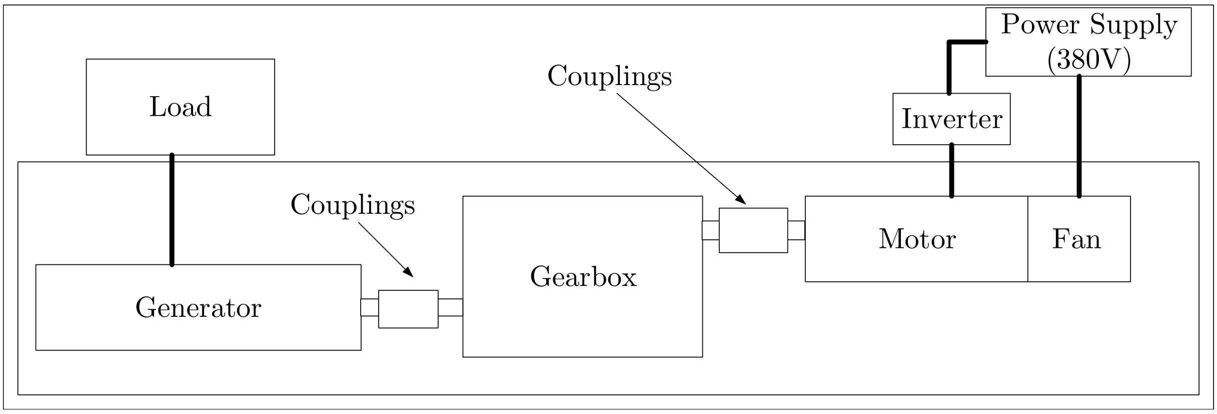


Supplementary Figure 10. The corresponding schematic diagram of the wind turbine system platform.

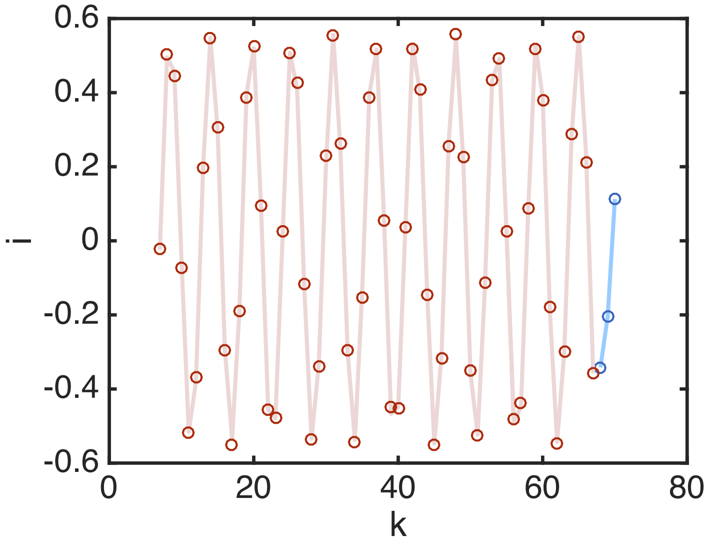


Supplementary Figure 11. The data fitting curve using data obtained from the wind turbine platform using IHYDE. The original time-series data (lines connecting the dots) is plotted in different colors associated with its subsystems. The fitted data from the identified models (dots), and the detected time of the switching (changes in colors) are illustrated.

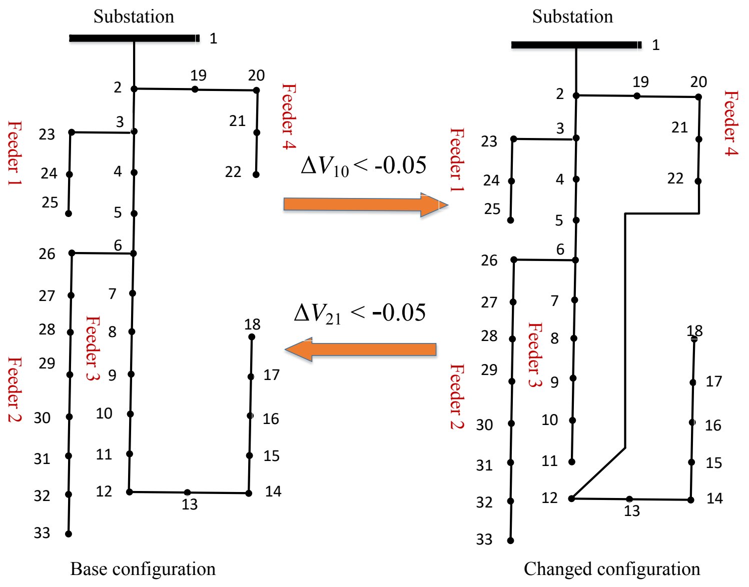


Supplementary Figure 12. Subsystem models and transition logic of the smart grid example.

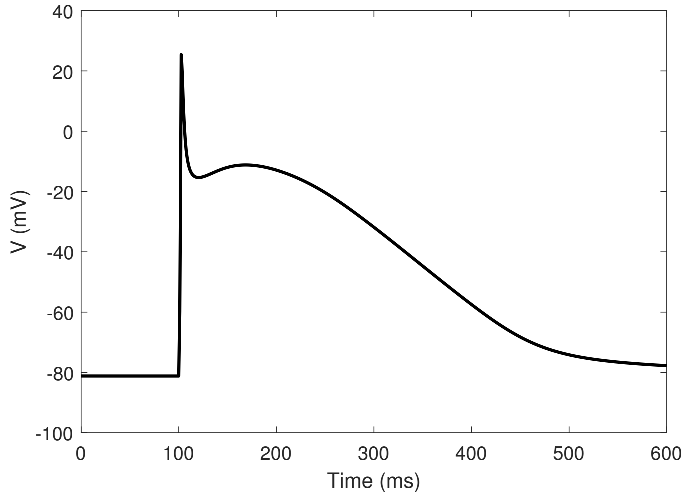


Supplementary Figure 13. Model action potential $V$ during stimulation at the frequency of 1 Hz.

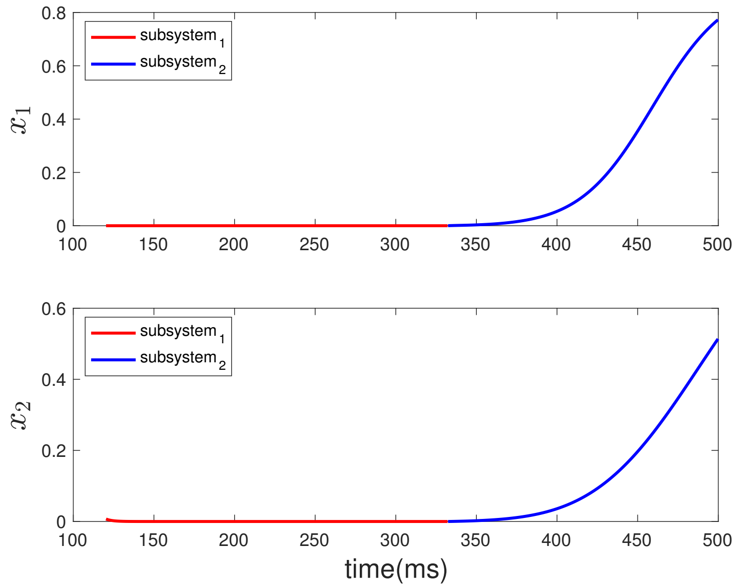


Supplementary Figure 14. The value of gating variable $x_1$ and $x_2$. Different colors denote data that are produced from different subsystems.

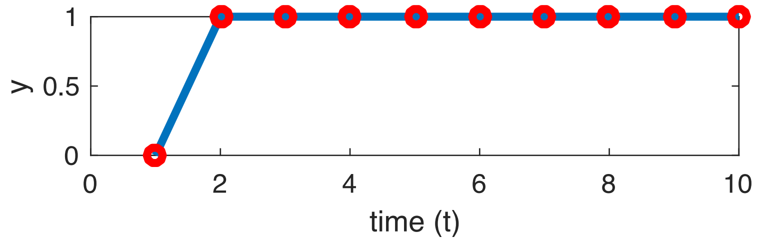


Supplementary Figure 15. The observed output of Eq. (19) when a Dirac delta function is applied to stimulate the system.

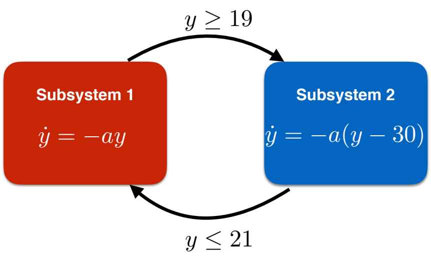


Supplementary Figure 16. A counterexample of a constructed hybrid dynamical system that is not able to be identified from data.

## SUPPLEMENTARY TABLES

Supplementary Table 1. The identified results of method in [17] and IHYDE

| Metrics | # of Iterations=1 | | # of iterations=5 |
|---|---|---|---|
| | Method [17] | IHYDE | IHYDE |
| parameters | $\lambda = 0.015,$<br>$\epsilon = 0.1$ | $\lambda_{\mathbf{z}} = 0.015, \epsilon_{\mathbf{z}} = 0.25,$<br>$\lambda_{\mathbf{w}} = 0.015, \epsilon_{\mathbf{w}} = 0.2$ | $\lambda_{\mathbf{z}} = 0.03, \epsilon_{\mathbf{z}} = 1e-4,$<br>$\lambda_{\mathbf{w}} = 0.008, \epsilon_{\mathbf{w}} = 0.1$ |
| Number<br>of Systems | 2 | 2 | 2 |
| Number of misclassified points | 96 | 75 | 41 |
| Dictionary | $1 \quad u \quad u^2 \quad u^3 \quad u^4 \quad u^5$ | | |
| Actual Subsystems 1 | $y = 0.5u^2 + u - 0.5$ | | |
| Identified<br>Subsystems 1 | $y = 1.0371u^5 - 0.1238u^4$<br>$-0.6507u^3 + 0.5462u^2$<br>$+1.0627u - 0.5005$ | $y = 0.0753u^4 + 0.3866u^2$<br>$+1.0079u - 0.4844$ | $y = 0.4841u^2$<br>$+0.9978u - 0.4984$ |
| Actual Subsystems 2 | $y = -0.5u^2 + u + 0.5$ | | |
| Identified<br>Subsystems 2 | $y = -0.6749u^5 + 0.2908u^4$<br>$+0.8616u^3 - 0.7386u^2$<br>$+0.8132u + 0.4322$ | $y = -0.5417u^2$<br>$+1.0613u + 0.4829$ | $y = -0.4806u^2$<br>$+0.9995u + 0.4882$ |

Supplementary Table 2. The identified subsystems and the selected hyperparameters based on the minimum error principle.

| Data Set | $\lambda_{\mathbf{z}}$ | $\lambda_{\mathbf{w}}$ | $\epsilon_{\mathbf{z}}$ | $\epsilon_{\mathbf{w}}$ | Mode ($m_k$) | Actual subsystem | Identified subsystem | Dictionary |
|---|---|---|---|---|---|---|---|---|
| Hysteresis Relay | 0.1 | 0.01 | $1e-4$ | 0.0224 | 1 | $y=1$ | $y=1.0020$ | polynomials in $u$ up to $5^{th}$ order |
| | | | | | 2 | $y=-1$ | $y=-1.0014$ | |
| Continuous Hysteresis Loop | 0.1 | 0.1 | $1e-4$ | 0.1184 | 1 | $y=0.5u^2 + u-0.5$ | $y=0.4275u^2 + 0.9954u-0.4802$ | polynomials in $u$ up to $5^{th}$ order |
| | | | | | 2 | $y=-0.5u^2 +u+0.5$ | $y=-0.5226u^2 + 1.0190u+0.4999$ | |
| Phototaxis Robot | $1e-3$ | 0.1 | $1e-4$ | 0.1619 | 1 | $y=u_2-u_1$ | $y=0.9947u_2 - 0.9947u_1$ | $1, u_1-u_2, \frac{1}{u_1-u_2}, u_1^2, u_2^2$ |
| | | | | | 2 | $y=\frac{1}{u_1-u_2}$ | $y=\frac{0.9707}{u_1-u_2}$ | |
| | | | | | 3 | $y=0$ | $y=0.0062u_1 - 0.0062u_2$ | |
| Nonlinear-Hybrid-System | $1e-4$ | 0.01 | $1e-4$ | 1.2036 | 1 | $y=u_1u_2$ | $y=0.9951u_1u_2$ | $\frac{u_1+u_2}{u_1-u_2}, \frac{u_1}{6+u_2}, u_1u_2, u_1, u_2, \sin(u_1), \sin(u_2), u_1^2, u_2^2$ |
| | | | | | 2 | $y=\frac{6u_1}{6+u_2}$ | $y=\frac{5.9567u_1}{6+u_2}$ | |
| | | | | | 3 | $y=\frac{u_1+u_2}{u_1-u_2}$ | $y=0.9958\frac{u_1+u_2}{u_1-u_2}$ | |

Supplementary Table 3. A summary of datasets used for IHYDE.

| Data Set | Modes | Subsystem models | # of Points | Next Mode | Transition logics | No. of Transitions |
|---|---|---|---|---|---|---|
| Hysteresis Relay | 1 | y=1 | 1004 | 2 | $u > 0.5$ | 33 |
| | 2 | y=-1 | 996 | 1 | $u < -0.5$ | 32 |
| Continuous Hysteresis Loop | 1 | $y = 0.5u^2 + u - 0.5$ | 999 | 2 | $u > 0.98$ | 21 |
| | 2 | $y = -0.5u^2 + u + 0.5$ | 1001 | 1 | $u < -0.98$ | 21 |
| Phototaxis Robot | 1 | $y = u_2 - u_1$ | 654 | 2 | $u_4 = 1$ | 10 |
| | | | | 3 | $u_5 = 1$ | 18 |
| | 2 | $y = \frac{1}{u_1 - u_2}$ | 585 | 1 | $u_3 = 1$ | 14 |
| | | | | 3 | $u_5 = 1$ | 11 |
| | 3 | $y = 0$ | 761 | 1 | $u_3 = 1$ | 15 |
| | | | | 2 | $u_4 = 1$ | 14 |
| Nonlinear Hybrid System | 1 | $y = u_1 u_2$ | 605 | 3 | $u_1^2 + u_2^2 < 9$ | 157 |
| | 2 | $y = \frac{6u_1}{6+u_2}$ | 738 | 1 | $u_1^2 + u_2^2 > 25$ | 158 |
| | 3 | $y = \frac{u_1+u_2}{u_1-u_2}$ | 657 | 2 | $u_1 u_2 < 0$ | 157 |
| Autonomous Car (experimental data) | 1 | $\Delta u(k) = 9.5(380 - v(k))$ $+48(v(k-1) - v(k))$ | 147 | 2 | $straight = 0$ | 2 |
| | 2 | $\Delta u(k) = 9.5(280 - v(k))$ $+48(v(k-1) - v(k))$ | 249 | 1 | $straight = 1$ | 2 |
| Chua's Circuit (experimental data) | 1 | $10^{-5}\frac{dy_1}{dt} = 1.0858y_2$ $-0.2115y_1 - 0.5349$ | 57 | 2 | $y_1 > -1.5$ | 2 |
| | 2 | $10^{-5}\frac{dy_1}{dt} = 1.0858y_2$ $+0.1451y_1$ | 21 | 1 | $y_1 < -1.5$ | 2 |
| | | | | 3 | $y_1 > 1.5$ | 1 |
| | 3 | $10^{-5}\frac{dy_1}{dt} = 1.0858y_2$ $-0.1994y_1 + 0.5168$ | 99 | 2 | $y_1 < 1.5$ | 2 |
| Wind turbine (experimental data) | 1 | Normal | 61 | 2 | $k > 67$ | 1 |
| | 2 | Fault | 3 | | | 0 |
| Power Grid Fault Detection | 1 | Normal | 30 | 2 | $t > 30$ | 1 |
| | 2 | Fault | 10 | 1 | | 0 |
| Smart Grid | 1 | Topology1 | 180 | 2 | $\Delta V_{10} < -0.05$ | 3 |
| | 2 | Topology2 | 180 | 1 | $\Delta V_{21} < -0.05$ | 2 |
| Human Atrial Action Potential Models | 1 | Normal | 707 | 2 | $V < -40$ | 1 |
| | 2 | Disease | 560 | 1 | | 0 |

Supplementary Table 4. The detailed information of Hysteresis Relay data.

| | |
|---|---|
| Original data points | 2000 |
| Sampling rate | 1 |
| Used data points | 2000 |
| Number of points (subsystem 1) | 1004 |
| Number of points (subsystem 2) | 996 |
| Number of transitions (S1 to S2) | 33 |
| Number of transitions (S2 to S1) | 32 |

Supplementary Table 5. The identified result and details of Hysteresis Relay.

| Noise | $N_p = 0$ | $N_p = 2\%$ | $N_p = 4\%$ | $N_p = 6\%$ |
|---|---|---|---|---|
| Library $\mathbf{\Phi}$ | 1 | | | |
| Identified subsystem 1 | $y = 0.9999$ | $y = 1.0013$ | $y = 1.0027$ | $y = 1.0040$ |
| Identified subsystem 2 | $y = -0.9999$ | $y = -1.0004$ | $y = -1.0009$ | $y = -1.0014$ |
| $\lambda_{\mathbf{z}}$ | $1e-3$ | | | |
| $\epsilon_{\mathbf{z}}$ | $1e-4$ | | | |
| $\lambda_{\mathbf{w}}$ | 0.05 | | | |
| $\epsilon_{\mathbf{w}}$ | 0.2 | | | |
| Number of misclassified points | 0 | | | |

Supplementary Table 6. The identified result and details of Hysteresis Relay with redundant dictionary functions.

| Noise | $N_p = 0$ | $N_p = 2\%$ | $N_p = 4\%$ | $N_p = 6\%$ |
|---|---|---|---|---|
| Library $\mathbf{\Phi}$ | $1\ u\ u^2\ u^3\ u^4\ u^5$ | | | |
| Identified subsystem 1 | $y = 0.9999$ | $y = 1.0013$ | $y = 1.0027$ | $y = 1.0038$ |
| Identified subsystem 2 | $y = -0.9999$ | $y = -1.0004$ | $y = -1.0009$ | $y = -1.0014$ |
| $\lambda_{\mathbf{z}}$ | $1e - 3$ | | | |
| $\epsilon_{\mathbf{z}}$ | $1e - 4$ | | | |
| $\lambda_{\mathbf{w}}$ | 0.05 | | | |
| $\epsilon_{\mathbf{w}}$ | 0.2 | | | |
| Number of misclassified points | 0 | | | |

Supplementary Table 7. The identified transition logics of Hysteresis Relay.

| Systems | Subsystem 1 | Subsystem 2 |
|---|---|---|
| Subsystem 1 | | $u > 0.4995$ |
| Subsystem 2 | $u < -0.4987$ | |
| Library $\mathbf{\Psi}$ | $1 \quad u$ | |
| $\beta$ | 0.1 | |

Supplementary Table 8. The identified transition logics of Hysteresis Relay when existing redundant dictionary functions.

| Systems | Subsystem 1 | Subsystem 2 |
|---|---|---|
| Subsystem 1 | | $u > 0.4995$ |
| Subsystem 2 | $u < -0.4987$ | |
| Library $\mathbf{\Psi}$ | $1 \quad u \quad e^{(10(\sin(u^2))+10)} \quad \frac{\log(\lvert u \rvert)}{\sin(u)} \quad u^2 \quad u^4$ | |
| $\beta$ | 0.1 | |

Supplementary Table 9. The detailed information of Continuous Hysteresis Loop data.

| | |
|---|---|
| Original data points | 2000 |
| Sampling rate | 1 |
| Used data points | 2000 |
| Number of points (subsystem 1) | 999 |
| Number of points (subsystem 2) | 1001 |
| Number of transitions (S1 to S2) | 21 |
| Number of transitions (S2 to S1) | 21 |

Supplementary Table 10. The identified systems of Continuous Hysteresis Loop for both noiseless and noisy datasets. We also present the tuning parameters for different noise levels.

| Noise | $N_p = 0$ | $N_p = 2\%$ | $N_p = 4\%$ | $N_p = 6\%$ |
|---|---|---|---|---|
| Library $\mathbf{\Phi}$ | polynomials in $u$ up to second order | | | |
| Identified subsystem 1 | $y = 0.4998u^2$ $+1.0000u$ $-0.4999$ | $y = 0.4989u^2$ $+1.0003u$ $-0.4996$ | $y = 0.4920u^2$ $+0.9999u$ $-0.4982$ | $y = 0.4842u^2$ $+0.9980u$ $-0.4961$ |
| Identified subsystem 2 | $y = -0.5042u^2$ $+1.0021u$ $+0.5008$ | $y = -0.5072u^2$ $+1.0031u$ $+0.5015$ | $y = -0.5172u^2$ $+1.0055u$ $+0.5032$ | $y = -0.5133u^2$ $+0.9966u$ $+0.5027$ |
| $\lambda_{\mathbf{z}}$ | 0.005 | 0.005 | 0.008 | 0.03 |
| $\epsilon_{\mathbf{z}}$ | $1e-4$ | | | |
| $\lambda_{\mathbf{w}}$ | 0.005 | 0.001 | 0.005 | 0.008 |
| $\epsilon_{\mathbf{w}}$ | 0.04 | 0.04 | 0.08 | 0.105 |
| Number of misclassified points | 0 | 17 | 45 | 88 |

Supplementary Table 11. The identified result and details of Continuous Hysteresis Loop with redundant dictionary functions.

| Noise | $N_p = 0$ | $N_p = 2\%$ | $N_p = 4\%$ | $N_p = 6\%$ |
|---|---|---|---|---|
| Library $\mathbf{\Phi}$ | $1\ u\ u^2\ e^{-u}\ \frac{u^3}{e^u}\ \frac{\cos(2u)}{\sin(u)^3}$ | | | |
| Identified subsystem 1 | $y = 0.4999u^2$ $+1.0000u$ $-0.4999$ | $y = 0.5001u^2$ $+1.0001u$ $-0.4998$ | $y = 0.4919u^2$ $+0.9995u$ $-0.4979$ | $y = 0.4811u^2$ $+0.9994u$ $-0.4956$ |
| Identified subsystem 2 | $y = -0.5010u^2$ $+0.9995u$ $+0.5002$ | $y = -0.4979u^2$ $+0.9990u$ $+0.5001$ | $y = -0.5123u^2$ $+1.0000u$ $+0.5034$ | $y = -0.5275u^2$ $+0.9999u$ $+0.5047$ |
| $\lambda_{\mathbf{z}}$ | 0.005 | 0.005 | 0.008 | 0.03 |
| $\epsilon_{\mathbf{z}}$ | $1e-4$ | | | |
| $\lambda_{\mathbf{w}}$ | 0.005 | 0.001 | 0.005 | 0.008 |
| $\epsilon_{\mathbf{w}}$ | 0.04 | 0.04 | 0.08 | 0.105 |
| Number of misclassified points | 0 | 11 | 45 | 94 |

Supplementary Table 12. The identified transition logics of the Continuous Hysteresis Loop without redundant dictionary functions using noiseless data.

| System | Subsystem 1 | Subsystem 2 |
|---|---|---|
| Subsystem 1 | | $u > 0.9803$ |
| Subsystem 2 | $u < -0.9799$ | |
| Library $\Psi$ | $1\quad u$ | |
| $\beta$ | 10 | |

Supplementary Table 13. The identified transition logics of the Continuous Hysteresis Loop with noiseless data and redundant dictionary functions.

| System | Subsystem 1 | Subsystem 2 |
|---|---|---|
| Subsystem 1 | | $u > 0.9803$ |
| Subsystem 2 | $u < -0.9799$ | |
| Library $\mathbf{\Psi}$ | $1 \quad u \quad \frac{1}{u^2} \quad \frac{\cos u}{\sin u^3}$ | |
| $\beta$ | 10 | |

Supplementary Table 14. The detailed information of Phototaxis Robot data.

| | |
|---|---|
| Original data points | 2000 |
| Sampling rate | 1 |
| Used data points | 2000 |
| Number of points (subsystem 1) | 654 |
| Number of points (subsystem 2) | 585 |
| Number of points (subsystem 3) | 761 |

Supplementary Table 15. The transition distribution for Phototaxis Robot example.

| System | Subsystem 1 | Subsystem 2 | Subsystem 3 |
|---|---|---|---|
| Subsystem 1 | | 14 | 15 |
| Subsystem 2 | 10 | | 14 |
| Subsystem 3 | 18 | 11 | |

Supplementary Table 16. The identified result and details of tuning parameters in the Phototaxis Robot example without reduntant dictionary functions.

| Noise | $N_p = 0$ | $N_p = 2\%$ | $N_p = 4\%$ | $N_p = 6\%$ |
|---|---|---|---|---|
| Library $\mathbf{\Phi}$ | $u_1 - u_2 \quad \frac{1}{u_1-u_2}$ | | | |
| Identified subsystem 1 | $y = -0.9980 (u_1 - u_2)$ | $y = -0.9978 (u_1 - u_2)$ | $y = -0.9964 (u_1 - u_2)$ | $y = -0.9955 (u_1 - u_2)$ |
| Identified subsystem 2 | $y = \frac{0.9908}{u_1-u_2}$ | $y = \frac{0.9947}{u_1-u_2}$ | $y = \frac{0.9820}{u_1-u_2}$ | $y = \frac{0.9821}{u_1-u_2}$ |
| Identified subsystem 3 | $y = 0$ | $y = 0$ | $y = 0.0068(u_1 - u_2)$ | $y = 0.0095(u_1 - u_2)$ |
| $\lambda_{\mathbf{z}}$ | $5e-4$ | $5e-4$ | $5e-4$ | $0.001$ |
| $\epsilon_{\mathbf{z}}$ | $1e-4$ | | | |
| $\lambda_{\mathbf{w}}$ | 0.05 | 0.05 | 0.1 | 0.1 |
| $\epsilon_{\mathbf{w}}$ | 0.05 | 0.06 | 0.2 | 0.2 |
| Number of misclassified points | 0 | 11 | 28 | 47 |

Supplementary Table 17. The identified result and details of tuning parameters in the Phototaxis Robot example with redundant dictionary functions.

| Noise | $N_p = 0$ | $N_p = 2\%$ | $N_p = 4\%$ | $N_p = 6\%$ |
|---|---|---|---|---|
| Library $\mathbf{\Phi}$ | $1 \quad u_1 - u_2 \quad \frac{1}{u_1-u_2} \quad u_1^2 \quad u_2^2$ | | | |
| Identified subsystem 1 | $y = -1.0000$ $(u_1 - u_2)$ | $y = -0.9957$ $(u_1 - u_2)$ | $y = -0.9944$ $(u_1 - u_2)$ | $y = -0.9941$ $(u_1 - u_2)$ |
| Identified subsystem 2 | $y = \frac{0.9998}{u_1-u_2}$ | $y = \frac{0.9891}{u_1-u_2}$ | $y = \frac{0.9727}{u_1-u_2}$ | $y = \frac{0.9689}{u_1-u_2}$ |
| Identified subsystem 3 | $y = 0$ | $y = -0.0002u_2^2$ | $y = 0.0046(u_1$ $-u_2) + 0.0014u_1^2$ | $y = 0.0064(u_1$ $-u_2) + 0.0019u_1^2$ |
| $\lambda_{\mathbf{z}}$ | $1e-4$ | $1e-4$ | $5e-4$ | $1e-3$ |
| $\epsilon_{\mathbf{z}}$ | $1e-4$ | | | |
| $\lambda_{\mathbf{w}}$ | $1e-3$ | 0.1 | 0.15 | 0.15 |
| $\epsilon_{\mathbf{w}}$ | 0.005 | 0.06 | 0.2 | 0.2 |
| Number of misclassified points | 0 | 11 | 28 | 48 |

Supplementary Table 18. The identified result and details of tuning parameters in the Phototaxis Robot example.

| System | Subsystem 1 | Subsystem 2 | Subsystem 3 |
|---|---|---|---|
| Subsystem 1 | | $u_3 < 0.4986u_4$ | $u_3 < 0.5085u_5$ |
| Subsystem 2 | $u_4 < 0.4553u_3$ | | $u_4 < 0.5055u_5$ |
| Subsystem 3 | $u_5 < 0.5242u_3$ | $u_5 < 0.4543u_4$ | |
| Library $\mathbf{\Psi}$ | $1 \quad u_3 \quad u_4 \quad u_5$ | | |
| $\beta$ | 0.5 | | |

Supplementary Table 19. The identified transition logics for the Phototaxis Robot example.

| System | Subsystem 1 | Subsystem 2 | Subsystem 3 |
|---|---|---|---|
| Subsystem 1 | | $u_3 < 0.4986u_4$ | $u_3 < 0.5085u_5$ |
| Subsystem 2 | $u_4 < 0.4553u_3$ | | $u_4 < 0.5055u_5$ |
| Subsystem 3 | $u_5 < 0.5242u_3$ | $u_5 < 0.4543u_4$ | |
| Library $\mathbf{\Psi}$ | $1 \quad u_1^{-1} \quad u_2 \quad \sin(u_1) \quad \cos(u_2) \quad e^{u_1u_2} \quad u_3 \quad u_4 \quad u_5$ | | |
| $\beta$ | 0.5 | | |

Supplementary Table 20. The detailed information of Nonlinear Hybrid System data.

| | |
|---|---|
| Original data points | 2000 |
| Sampling rate | 1 |
| Used data points | 2000 |
| Number of points (subsystem 1) | 605 |
| Number of points (subsystem 2) | 738 |
| Number of points (subsystem 3) | 657 |

Supplementary Table 21. The transition distribution for Nonlinear Hybrid System example.

| System | Subsystem 1 | Subsystem 2 | Subsystem 3 |
|---|---|---|---|
| Subsystem 1 | | | 157 |
| Subsystem 2 | 158 | | |
| Subsystem 3 | | 157 | |

Supplementary Table 22. The identified result and details of Nonlinear Hybrid System.

| Noise | $N_p = 0$ | $N_p = 2\%$ | $N_p = 4\%$ | $N_p = 6\%$ |
|---|---|---|---|---|
| Library $\mathbf{\Phi}$ | $u_1u_2 \quad \frac{6u_1}{6+u_2} \quad \frac{u_1+u_2}{u_1-u_2}$ | | | |
| Identified subsystem 1 | $y = 0.9998u_1u_2$ | $y = 0.9958u_1u_2$ | $y = 0.9962u_1u_2$ | $y = 0.9953u_1u_2$ |
| Identified subsystem 2 | $y = 0.9983\frac{6u_1}{6+u_2}$ | $y = 0.9961\frac{6u_1}{6+u_2}$ | $y = 0.9947\frac{6u_1}{6+u_2}$ | $y = 0.9939\frac{6u_1}{6+u_2}$ |
| Identified subsystem 3 | $y = 0.9990\frac{u_1+u_2}{u_1-u_2}$ | $y = 0.9945\frac{u_1+u_2}{u_1-u_2}$ | $y = 0.9901\frac{u_1+u_2}{u_1-u_2}$ | $y = 0.9949\frac{u_1+u_2}{u_1-u_2}$ |
| $\lambda_{\mathbf{z}}$ | $1e-6$ | $1.5e-4$ | $1.5e-4$ | $1.5e-4$ |
| $\epsilon_{\mathbf{z}}$ | $1e-4$ | | | |
| $\lambda_{\mathbf{w}}$ | 0.03 | | | |
| $\epsilon_{\mathbf{w}}$ | 0.6 | 2 | 2 | 2 |
| Number of misclassified points | 0 | 63 | 129 | 177 |

Supplementary Table 23. The identified result and details of Nonlinear Hybrid Systems when there exists redundant dictionary functions.

| Noise | $N_p = 0$ | $N_p = 2\%$ | $N_p = 4\%$ | $N_p = 6\%$ |
|---|---|---|---|---|
| Library $\mathbf{\Phi}$ | $u_1u_2 \quad \frac{6u_1}{6+u_2} \quad \frac{u_1+u_2}{u_1-u_2} \quad u_1 \quad u_2 \quad \sin(u_1) \quad \sin(u_2) \quad u_1^2 \quad u_2^2$ | | | |
| Identified subsystem 1 | $y = 0.9997u_1u_2$ | $y = 0.9999u_1u_2$ | $y = 0.9944u_1u_2$ | $y = 0.9953u_1u_2$ |
| Identified subsystem 2 | $y = 0.9983\frac{6u_1}{6+u_2}$ | $y = 0.9960\frac{6u_1}{6+u_2}$ | $y = 0.9960\frac{6u_1}{6+u_2}$ | $y = 0.9963\frac{6u_1}{6+u_2}$ |
| Identified subsystem 3 | $y = 0.9990\frac{u_1+u_2}{u_1-u_2}$ | $y = 0.9975\frac{u_1+u_2}{u_1-u_2}$ | $y = 0.9898\frac{u_1+u_2}{u_1-u_2}$ | $y = 0.9877\frac{u_1+u_2}{u_1-u_2}$ |
| $\lambda_{\mathbf{z}}$ | $1e-5$ | $5e-5$ | $1.5e-4$ | $1.5e-4$ |
| $\epsilon_{\mathbf{z}}$ | $1e-4$ | | | |
| $\lambda_{\mathbf{w}}$ | 0.03 | 0.03 | 0.032 | 0.0282 |
| $\epsilon_{\mathbf{w}}$ | 0.6 | 0.8 | 2 | 2 |
| Number of misclassified points | 0 | 67 | 129 | 175 |

Supplementary Table 24. The identified transition logics of Nonlinear Hybrid System.

| System | Subsystem 1 | Subsystem 2 | Subsystem 3 |
|---|---|---|---|
| Subsystem 1 | | | $u_1^2 + u_2^2 < 8.9993$ |
| Subsystem 2 | $u_1^2 + u_2^2 > 24.9803$ | | |
| Subsystem 3 | | $u_1u_2 < -0.013$ | |
| Library $\mathbf{\Psi}$ | $1 \quad u_1u_2 \quad u_1^2 + u_2^2$ | | |
| $\beta$ | 0.01 | | |

Supplementary Table 25. The identified transition logics of Nonlinear Hybrid System when there are redundant dictionary functions.

| System | Subsystem 1 | Subsystem 2 | Subsystem 3 |
|---|---|---|---|
| Subsystem 1 | | | $34.6143u_1^2 + 35.8259u_2^2 < 316.4377$ |
| Subsystem 2 | $11.8852u_1^2 + 11.8905u_2^2 > 296.5977$ | | |
| Subsystem 3 | | $u_1u_2 < -0.013$ | |
| Library $\mathbf{\Psi}$ | $1 \quad u_1 \quad u_2 \quad e^{u_1+u_2} \quad u_1u_2 \quad u_1^2 \quad u_2^2$ | | |
| $\beta$ | 0.01 | | |

Supplementary Table 26. The detailed information of experiment data from autonomous car example.

| | |
|---|---|
| Original data points | 400 |
| Used data points | 396 |
| Number of points (Straightway) | 147 |
| Number of points (curve) | 249 |
| Number of transitions (straightway to curve) | 2 |
| Number of transitions (curve to straightway) | 2 |

Supplementary Table 27. The identified transition logics of autonomous car testbed.

| System | Straightaway | Curve |
|---|---|---|
| Straightaway | | straight $< 0.3318$ |
| Curve | straight $> 0.6072$ | |
| Library $\mathbf{\Psi}$ | 1 straight $\sin(v(k))$ $\cos(v(k))$ $\tan(v(k))$ $\frac{v(k-1)-v(k-4)}{v(k-2)}$ $v(k-1)\tan(v(k-3))$ | |
| $\beta$ | 0.001 | |

Supplementary Table 28. The identified result and details of autonomous car testbed.

| Library $\mathbf{\Phi}$ | $1 \quad v(k) \quad v(k-1)$ | |
|---|---|---|
| Speed control strategy | Straightaway | Curve |
| True strategy | $\Delta u(k) = 9.5(380 - v(k))$ $+48(v(k-1) - v(k))$ | $\Delta u(k) = 9.5(280 - v(k))$ $+48(v(k-1) - v(k))$ |
| True times | 147 | 249 |
| Identified strategy | $\Delta u(k) = 9.4957(379.9550 - v(k))$ $+47.9742(v(k-1) - v(k))$ | $\Delta u(k) = 9.4960(279.9462 - v(k))$ $+47.9888(v(k-1) - v(k))$ |
| Identified switching times | 147 | 249 |
| $\lambda_{\mathbf{z}}$ | 0.01 | |
| $\epsilon_{\mathbf{z}}$ | 100 | |
| $\lambda_{\mathbf{w}}$ | $1e-5$ | |
| $\epsilon_{\mathbf{w}}$ | 8 | |

Supplementary Table 29. The identified result and details of autonomous car testbed with redundant dictionary functions.

| Library $\mathbf{\Phi}$ | all the polynomial combinations of $v(k), \ldots, v(k-4)$ to fourth order | |
|---|---|---|
| Speed control strategy | Straightaway | Curve |
| True strategy | $\Delta u(k) = 9.5(380 - v(k))$ $+48(v(k-1) - v(k))$ | $\Delta u(k) = 9.5(280 - v(k))$ $+48(v(k-1) - v(k))$ |
| True times | 147 | 249 |
| Identified strategy | $\Delta u(k) = 9.4957(379.9554 - v(k))$ $+47.9741v(k-1) - v(k))$ | $\Delta u(k) = 9.4959(279.9465 - v(k))$ $+47.9888(v(k-1) - v(k))$ |
| Identified switching times | 147 | 249 |
| $\lambda_{\mathbf{z}}$ | 0.01 | |
| $\epsilon_{\mathbf{z}}$ | 100 | |
| $\lambda_{\mathbf{w}}$ | $1e-5$ | |
| $\epsilon_{\mathbf{w}}$ | 8 | |

Supplementary Table 30. The detailed information of experiment data from Chua's circuit.

| | |
|---|---|
| Original data points | 120000 |
| Sampling rate | $5 \times 10^6$Hz |
| Down sampling | 1:50:120000 |
| Used data points | 177 |
| Number of points (subsystem 1) | 57 |
| Number of points (subsystem 2) | 21 |
| Number of points (subsystem 3) | 99 |

Supplementary Table 31. The transition distribution of experiment data from Chua's circuit.

| System | Subsystem 1 | Subsystem 2 | Subsystem 3 |
|---|---|---|---|
| Subsystem 1 | | 2 | |
| Subsystem 2 | 2 | | 1 |
| Subsystem 3 | | 2 | |

Supplementary Table 32. True parameters of the built Chua's circuit.

| Item | Value | Item | Value |
|---|---|---|---|
| $a$ | $-1.2309e-3$ | $c_1$ | $0.01\mu F$ |
| $b$ | $-8.743e-4$ | $c_2$ | $0.1\mu F$ |
| $b'$ | $-8.864e-4$ | $dt$ | $10^{-5}s$ |
| $\tau$ | 1.5 | $L$ | $6.8mH$ |
| $R$ | 921 | $E$ | $1.5V$ |

Supplementary Table 33. The identified subsystems and transition logics of Chua's circuit which contains all subsystems with redundant dictionary functions.

| | |
|---|---|
| Library $\mathbf{\Phi}$ | $1\ y_1\ y_2\ e^{y_1}\ \frac{y_1}{y_2}\ \frac{\cos(0.1y_1)^2}{1+y_2^2}\ \cos(y_1+y_2)^2$ |
| Identified subsystems | $10^{-5}\frac{dy_1}{dt} = 1.0758y_2 - 0.2028y_1 - 0.5405 \quad y_1 < -1.4348$<br>$10^{-5}\frac{dy_1}{dt} = 1.0793y_2 + 0.1576y_1 \quad -1.5627 < y_1 < 1.3137$<br>$10^{-5}\frac{dy_1}{dt} = 1.0869y_2 - 0.2127y_1 + 0.4859 \quad y_1 > 1.4627$ |
| $\lambda_{\mathbf{z}}$ | 0.05 |
| $\epsilon_{\mathbf{z}}$ | 0.012 |
| $\lambda_{\mathbf{w}}$ | 0.01 |
| $\epsilon_{\mathbf{w}}$ | 0.044 |
| Library $\mathbf{\Psi}$ | $1\quad y_1\quad y_2\quad \sin(y_2)\cos(y_1)\quad \frac{\frac{dy_1}{dt}}{\sin(y_1)+\frac{dy_1}{dt}}\quad \frac{\frac{dy_1}{dt}}{y_2}\quad \frac{dy_1}{dt}$ |
| $\beta$ | 0.01 |

Supplementary Table 34. The detailed information of experiment data from wind turbine system platform.

| | |
|---|---|
| Original data points | 20701 |
| Sampling rate | 1000Hz |
| Down sampling | 1:300:20701 |
| Used data points | 64 |
| Number of points (normal) | 61 |
| Number of points (fault) | 3 |
| Number of transitions | 1 |

Supplementary Table 35. The identified result and details of the gearbox broken tooth fault detection with redundant dictionary functions.

| System | Gearbox |
|---|---|
| True fault time | 68 |
| Identified fault time | 68 |
| $\lambda_{\mathbf{z}}$ | 1.5 |
| $\epsilon_{\mathbf{z}}$ | $1e-4$ |
| $\lambda_{\mathbf{w}}$ | $5e-5$ |
| $\epsilon_{\mathbf{w}}$ | 0.026 |
| Library $\mathbf{\Phi}$ | all the polynomial combinations of $y(k)\cdots y(k-5)$ to second order |
| Number of misclassified points | 0 |
| Library $\mathbf{\Psi}$ | 1 $k$ |
| $\beta$ | 0.1 |

Supplementary Table 36. The detailed information of data sampled during power system line fault detection.

| | |
|---|---|
| Original data points | 40 |
| Used data points | 40 |
| Number of points (normal) | 30 |
| Number of points (fault) | 10 |
| Normal to fault | 1 |

Supplementary Table 37. The identified result and details of power system fault detection.

| Bus | Bus 6 and bus 12 | Other bus except bus 1 | Bus 1 |
|---|---|---|---|
| True time for fault occurance | 31 | None | None |
| Identified time for fault occurance | 31 | None | None |
| $\lambda_{\mathbf{z}}$ | $1e-3$ | $1e-3$ | $1e-3$ |
| $\epsilon_{\mathbf{z}}$ | 0.008 | 0.008 | 0.008 |
| $\lambda_{\mathbf{w}}$ | $1e-6$ | $1e-6$ | $1e-9$ |
| $\epsilon_{\mathbf{w}}$ | 0.05 | 0.05 | 0.05 |
| Library $\mathbf{\Psi}$ | 1 $t$ | | |
| $\beta$ | 0.01 | None | None |

Supplementary Table 38. The detailed information of smart gird data.

| | |
|---|---|
| Original data points | 180 |
| Used data points | 180 |
| Number of points (subsystem 1) | 90 |
| Number of points (subsystem 2) | 90 |
| Number of transitions (S1 to S2) | 3 |
| Number of transitions (S2 to S1) | 2 |

Supplementary Table 39. The detailed parameters of switch operators.

| Transition rules | Time | Opened switch | Closed switch | Bus of load change |
|---|---|---|---|---|
| $\mathcal{T}_{1\to 2}$ | $31, 91, 151$ | $11 - 12$ | $12 - 22$ | $9, 10, 11$ |
| $\mathcal{T}_{2\to 1}$ | $61, 121$ | $12 - 22$ | $11 - 12$ | $20, 21, 22$ |

Supplementary Table 40. The identified result and detailed parameters.

| System model | Subsystem 1 | Subsystem 2 |
|---|---|---|
| True switching time | $31, 91, 151$ | $61, 121$ |
| Identified switching time | $31, 91, 151$ | $61, 121$ |
| $\lambda_{\mathbf{z}}$ | $5e-3$ | |
| $\epsilon_{\mathbf{z}}$ | $1.5e-2$ | |
| $\lambda_{\mathbf{w}}$ | $1e-6$ | |
| $\epsilon_{\mathbf{w}}$ | $5e-2$ | |
| Number of misclassified points | 0 | |

Supplementary Table 41. The identified transition logics for the model switching in smart grid.

| System | Subsystem 1 | Subsystem 2 |
|---|---|---|
| Subsystem 1 | | $\Delta V_{10} < -0.0499$ |
| Subsystem 2 | $\Delta V_{21} < -0.0472$ | |
| Library $\mathbf{\Psi}$ | $1 \;\; \Delta V_1 \cdots \Delta V_{33}$ | |
| $\beta$ | $5.8e-5$ | |

Supplementary Table 42. The detailed information of data sampled from AP model.

| | |
|---|---|
| Original data points | 120001 |
| Sampling rate | 200Hz |
| Down sampling | 24000:60:100000 |
| Used data points | 1267 |
| Number of points (subsystem 1) | 707 |
| Number of points (subsystem 2) | 560 |
| Number of transitions (S1 to S2) | 1 |

Supplementary Table 43. The identified results and detailed parameters of AP model.

| Gating variable | $x_1$ | $x_2$ |
|---|---|---|
| Actual subsystem 1 | $\dot{x}_1 = -\rho_{12}x_1$ | $\dot{x}_2 = -0.3x_2\frac{\exp(-2.535\times10^{-7}V)}{1+\exp[-0.1(V+32)]}$ |
| Actual subsystem 2 | $\dot{x}_1 = 0.135\exp(-\frac{V+80}{6.8}) -$ $0.135x_1\exp(-\frac{V+80}{6.8}) - \rho_{11}x_1$ | $\dot{x}_2 = \alpha_{21} - \alpha_{21}x_2 -$ $0.1212x_2\frac{\exp(-0.01052V)}{1+\exp[-0.1378(V+40.14)]}$ |
| Actual change time | 332.10 ms | 332.10 ms |
| Identified subsystem 1 | $\dot{x}_1 = -0.9999\rho_{12}x_1$ | $\dot{x}_2 = -0.3000x_2\frac{\exp(-2.535\times10^{-7}V)}{1+\exp[-0.1(V+32)]}$ |
| Identified subsystem 2 | $\dot{x}_1 = 0.1349\exp(-\frac{V+80}{6.8}) -$ $0.1349\exp(-\frac{V+80}{6.8})x_1 -$ $0.9987\rho_{11}x_1$ | $\dot{x}_2 = 1.0000\alpha_{21} - 1.0000\alpha_{21}x_2 -$ $0.1212x_2\frac{\exp(-0.01052V)}{1+\exp[-0.1378(V+40.14)]}$ |
| Identified change time | 332.10 ms | 332.10 ms |
| $\lambda_{\mathbf{z}}$ | 1e-4 | 1e-4 |
| $\epsilon_{\mathbf{z}}$ | 3e-5 | 3e-5 |
| $\lambda_{\mathbf{w}}$ | 3e-5 | 1e-5 |
| $\epsilon_{\mathbf{w}}$ | 5e-5 | 5e-5 |
| Library $\mathbf{\Phi}$ | $\exp(-\frac{V+80}{6.8})$ $\exp(-\frac{V+80}{6.8})x_1$ $\rho_{11}x_1$ $\rho_{12}x_1$ | $\alpha_{21}$ $\alpha_{21}x_2$ $x_2\frac{\exp(-0.01052V)}{1+\exp[-0.1378(V+40.14)]}$ $x_2\frac{\exp(-2.535\times10^{-7}V)}{1+\exp[-0.1(V+32)]}$ |
| Number of misclassified points | 0 | 0 |

Supplementary Table 44. The identified transition logics for gating variable $x_1$.

| gating variable $x_1$ | Subsystem 1 | Subsystem 2 |
|---|---|---|
| Subsystem 1 | | $V < -40.0093$ |
| Library $\boldsymbol{\Psi}$ | 1 $V$ | |
| $\beta$ | 1e-6 | |

Supplementary Table 45. The identified transition logics for gating variable $x_2$.

| gating variable $x_2$ | Subsystem 1 | Subsystem 2 |
|---|---|---|
| Subsystem 1 | | $V < -40.0093$ |
| Library $\boldsymbol{\Psi}$ | 1 $V$ | |
| $\beta$ | 1e-6 | |

Supplementary Table 46. The detailed parameters and identified results of AP model with polynomial dictionary functions.

| Gating variable | $x_1$ | $x_2$ |
|---|---|---|
| Actual subsystem 1 | $\dot{x}_1 = -\rho_{12}x_1$ | $\dot{x}_2 = -0.3x_2\frac{\exp(-2.535\times10^{-7}V)}{1+\exp[-0.1(V+32)]}$ |
| Actual subsystem 2 | $\dot{x}_1 = 0.135\exp(-\frac{V+80}{6.8}) - 0.135x_1\exp(-\frac{V+80}{6.8}) - \rho_{11}x_1$ | $\dot{x}_2 = \alpha_{21} - x_2\alpha_{21} - 0.1212x_2\frac{\exp(-0.01052V)}{1+\exp[-0.1378(V+40.14)]}$ |
| Actual change time | 332.10 ms | 332.10 ms |
| Identified subsystem 1 | $\dot{x}_1 = 0.0006 - 0.0003V - 2.1791x_1 - 0.0608x_1V - 0.2323V^2 - 0.0004x_1V^2 - 0.0032x_1^2V - 0.0256V^3$ | $\dot{x}_2 = 0.0610x_2 - 0.0218x_2^2$ |
| Identified subsystem 2 | $\dot{x}_1 = -1.0506x_1^2 + 0.0762x_1V - 5.9332\times10^{32}x_1^3 - 0.0033x_1V^2 + 3.9061\times10^{31}x_1^2V + 7.9368\times10^{64}x_1^3$ | $\dot{x}_2 = -0.2525x_2 + 0.0003x_2^3$ |
| Identified change time | 331.80 ms | 331.80 ms |
| $\lambda_{\mathbf{z}}$ | 1e-4 | 1e-5 |
| $\epsilon_{\mathbf{z}}$ | 3e-5 | 3e-5 |
| $\lambda_{\mathbf{w}}$ | 3e-8 | 1e-4 |
| $\epsilon_{\mathbf{w}}$ | 5e-5 | 5e-5 |
| Library $\mathbf{\Phi}$ | polynomials of $V, x_1$ up to third order | polynomials of $V, x_2$ up to third order |
| Number of misclassified points | 1 | 1 |

Supplementary Table 47. The details of simulation datasets in [13].

| Systems | Form | Data used to train [13] | | Data used to train IHYDE | |
|---|---|---|---|---|---|
| | | time | points | time | points |
| Linear 2D | $\frac{d}{dt}\begin{bmatrix} x \\ y \end{bmatrix} = \begin{bmatrix} -0.1 & 2 \\ -2 & -0.1 \end{bmatrix}\begin{bmatrix} x \\ y \end{bmatrix}$ | $t \in [0, 25]$ | 2501 | $t \in [0, 10]$ | 1001 |
| Cubic 2D | $\frac{d}{dt}\begin{bmatrix} x \\ y \end{bmatrix} = \begin{bmatrix} -0.1 & 2 \\ -2 & -0.1 \end{bmatrix}\begin{bmatrix} x^3 \\ y^3 \end{bmatrix}$ | $t \in [0, 25]$ | 2501 | $t \in [0, 10]$ | 1001 |
| Linear 3D | $\frac{d}{dt}\begin{bmatrix} x \\ y \\ z \end{bmatrix} = \begin{bmatrix} -0.1 & 2 & 0 \\ -2 & -0.1 & 0 \\ 0 & 0 & -0.3 \end{bmatrix}\begin{bmatrix} x \\ y \\ z \end{bmatrix}$ | $t \in [0, 50]$ | 5001 | $t \in [0, 10]$ | 1001 |
| Logistic map | $x_{k+1} = \mu_k x_k(1 - x_k)$ <br> $\mu_{k+1} = \mu_k$ | | 9990 | | 990 |
| Lorenz system | $\dot{x} = 10y - 10x$ <br> $\dot{y} = 28x - xz - y$ <br> $\dot{z} = xy - 2.6667z$ | $t \in [0.001, 100]$ | 100000 | $t \in [0.01, 10]$ | 1000 |
| Lorenz TVDiff | $\dot{x} = 10y - 10x$ <br> $\dot{y} = 28x - xz - y$ <br> $\dot{z} = xy - 2.6667z$ | $t \in [0.001, 50]$ | 48002 | $t \in [0.001, 50]$ <br> downsampling=25 | 1921 |
| Hopf TVDiff | $\dot{x} = ux - y - x^3 - xy^2$ <br> $\dot{y} = x + uy - yx^2 - y^3$ <br> $\dot{u} = 0$ | | 399014 | downsampling=100 | 3991 |

Supplementary Table 48. The identified results using datasets in [13]. Seven prototypical systems are examined, IHYDE successfully discovers all of them. We highlight the number of data points required is much less than [13] shown in Supplementary Figure 47.

| | Identified systems by [13] | Identified systems by IHYDE |
|---|---|---|
| Linear 2D | $\frac{d}{dt}\begin{bmatrix} x \\ y \end{bmatrix} = \begin{bmatrix} -0.1015 & 2.0027 \\ -1.9990 & -0.0994 \end{bmatrix}\begin{bmatrix} x \\ y \end{bmatrix}$ | $\frac{d}{dt}\begin{bmatrix} x \\ y \end{bmatrix} = \begin{bmatrix} -0.0993 & 2.0054 \\ -2.0004 & -0.1048 \end{bmatrix}\begin{bmatrix} x \\ y \end{bmatrix}$ |
| Cubic 2D | $\frac{d}{dt}\begin{bmatrix} x \\ y \end{bmatrix} = \begin{bmatrix} -0.0996 & 1.9970 \\ -1.9994 & -0.0979 \end{bmatrix}\begin{bmatrix} x^3 \\ y^3 \end{bmatrix}$ | $\frac{d}{dt}\begin{bmatrix} x \\ y \end{bmatrix} = \begin{bmatrix} -0.1015 & 2.0005 \\ -2.0010 & -0.1002 \end{bmatrix}\begin{bmatrix} x^3 \\ y^3 \end{bmatrix}$ |
| Linear 3D | $\frac{d}{dt}\begin{bmatrix} x \\ y \\ z \end{bmatrix} = \begin{bmatrix} -0.0996 & 2.0005 & 0 \\ -1.9997 & -0.0994 & 0 \\ 0 & 0 & -0.3003 \end{bmatrix}\begin{bmatrix} x \\ y \\ z \end{bmatrix}$ | $\frac{d}{dt}\begin{bmatrix} x \\ y \\ z \end{bmatrix} = \begin{bmatrix} -0.0992 & 2.0002 & 0 \\ -1.9999 & -0.0991 & 0 \\ 0 & 0 & -0.2983 \end{bmatrix}\begin{bmatrix} x \\ y \\ z \end{bmatrix}$ |
| Logistic map | $x_{k+1} = \mu_k x_k(0.9993 - 0.9989x_k)$<br>$\mu_{k+1} = 1.0000\mu_k$ | $x_{k+1} = \mu_k x_k(1.0005 - 1.0006x_k)$<br>$\mu_{k+1} = 1.0000\mu_k$ |
| Lorenz system | $\dot{x} = 9.9998y - 9.9996x$<br>$\dot{y} = 27.9980x - 0.9999xz - 0.9997y$<br>$\dot{z} = 1.0000xy - 2.6665z$ | $\dot{x} = 10.0060y - 9.9968x$<br>$\dot{y} = 27.9480x - 0.9957xz - 0.9954y$<br>$\dot{z} = 1.0010xy - 2.6673z$ |
| Lorenz TVDiff | $\dot{x} = 9.9999y - 9.9856x$<br>$\dot{y} = 27.7382x - 0.9949xz - 0.8763y$<br>$\dot{z} = 1.0000xy - 2.6618z$ | $\dot{x} = 10.0087y - 10.0227x$<br>$\dot{y} = 27.6620x - 0.9934xz - 0.8461y$<br>$\dot{z} = 0.9993xy - 2.6640z$ |
| Hopf TVDiff | $\dot{x} = 0.9269ux - 0.9920y - 0.9208x^3$<br>$-0.9211xy^2$<br>$\dot{y} = 0.9914x + 0.9294uy - 0.9244yx^2$<br>$-0.9252y^3$<br>$\dot{u} = 0$ | $\dot{x} = 0.9193ux - 0.9921y - 0.9109x^3$<br>$-0.9179xy^2$<br>$\dot{y} = 0.9911x + 0.9164uy - 0.9127yx^2$<br>$-0.9130y^3$<br>$\dot{u} = 0$ |

Supplementary Table 49. The tuning parameters are presented for these prototypical examples.

| | Noise | $\lambda_{\mathbf{z}}$ | $\epsilon_{\mathbf{z}}$ | $\lambda_{\mathbf{w}}$ | $\epsilon_{\mathbf{w}}$ |
|---|---|---|---|---|---|
| Linear 2D | 0.05 | 1 | $1e-4$ | $2e-3$ | 0.2 |
| Cubic 2D | 0.05 | 1 | $1e-4$ | $2e-3$ | 0.2 |
| Linear 3D | 0.01 | 1 | $1e-4$ | $2e-3$ | 0.05 |
| Logistic map | 0.01 | 1 | $1e-4$ | $2e-3$ | 0.05 |
| Lorenz system | 1 | 1 | $1e-4$ | $1e-3$ | 4 |
| Lorenz TVDiff | 0.01 | 1 | $1e-4$ | $4e-3$ | 3 |
| Hopf TVDiff | 0.005 | 1 | $1e-4$ | $3e-3$ | 0.1 |

Supplementary Table 50. The selected hyperparameters and the identified subsystems for discussions in the choice of canonical dictionary functions.

| Library $\mathbf{\Phi}$ | polynomials of $x$ up to fifth order |
|---|---|
| Actual subsystem 1 | $\dot{x} = -x^3$ |
| Identified subsystem 1 | $\dot{x} = -0.9975x^3$ |
| Actual subsystem 2 | $\dot{x} = -\cos(x)$ |
| Identified subsystem 2 | $\dot{x} = -0.9960 + 0.4651x^2$ |
| $\lambda_{\mathbf{z}}$ | $1e-6$ |
| $\epsilon_{\mathbf{z}}$ | $1e-2$ |
| $\lambda_{\mathbf{w}}$ | $1e-4$ |
| $\epsilon_{\mathbf{w}}$ | 0.1826 |

Supplementary Table 51. Directories in the IHYDE toolbox.

| Directories | Description |
|---|---|
| /CPSid | main functions and examples |
| /CPSid/data | the used data sets |
| /CPSid/tools | functions for IHYDE |
| /CPSid/EX-grid-search | examples of grid search |
| /CPSid/EX-nonhybrid | examples of nonhybrid systems |
| /CPSid/SLR_dev | functions for sparse logistic regression |
| /CPSid/comparison | comparison with reference [17] |

Supplementary Table 52. The introduction of function library which constructs dictionary matrix for identification.

| Function library | Description |
|---|---|
| yin | an $M$ by $n$ matrix which contains time-course input-output data. In here, $M$ is the sample number, and $n$ is the number of variables. |
| memory | the historical data (previous memory time instants) is used in yin. |
| polyorder | used to construct the polynomial of the highest order (up to fifth order). |
| basis_function | add more dictionary functions. It can be turned off, if basis_function.work set as 'off'. |
| yout | constructed dictionary matrix $\mathbf{\Phi}$. |

Supplementary Table 53. The introduction of function ihyde. The ihyde can be used to identify each subsystem.

| Function ihyde | Description |
|---|---|
| parameter.y | the output data. |
| parameter.normalize_y | set to 1 if $\mathbf{y}$ need to be normalized. |
| parameter.max_s | the max number of subsystems that could be identified by IHYDE. |
| parameter.epsilon | a 2-dimensional parameter vector $[\epsilon_{\mathbf{z}}, \epsilon_{\mathbf{w}}]$. |
| parameter.lambda | a 2-dimensional parameter vector $[\lambda_{\mathbf{z}}, \lambda_{\mathbf{w}}]$. |
| parameter.Phi | the constructed matrix $\mathbf{\Phi}$. |
| parameter.MAXITER | the max number of iterations that the sparsesolver function solves. |
| result. idx_sys | the index of each subsystem. |
| result.sys | the model of each subsystem. |
| result.theta | $\mathbf{z}$ of each identified subsystem. |
| result.error | the fitting error. |

Supplementary Table 54. The introduction of function finetuning. Based on the minimum error principle, it finetunes the result from ihyde and outputs the final result.

| Function finetuning | Description |
|---|---|
| result.lambda | the trading-off parameter $\lambda$ of the `sparsesolver` function. Parameter.lamdba(2) is set as the default value. |
| result.epsilon | the threshold in finetuning. Parameter.epsilon(2) is set as the default value. |
| result.threshold | the threshold for subsystem clustering. |
| final_result.idx | the index of each subsystem. |
| final_result.sys | the model of each subsystem. |
| final_result.allerror | the error which compared with the true output. |

Supplementary Table 55. The introduction of function ihydelogic.

| Function ihydelogic | Description |
|---|---|
| para_log.Phi2 | constructed dictionary matrix $\boldsymbol{\Psi}$ for inferring transition logics of each subsystem. |
| para_log.idx_sys | the index of each subsystem. |
| para_log.beta | the tradeoff parameter in the $\ell_1$ regularized sparse logistic regression. |
| para_log.y | the output data. |
| para_log.normalize | set to 1 if $\boldsymbol{\Psi}$ need to be normalized. |

## SUPPLEMENTARY REFERENCES


[1] Lygeros, J., Tomlin, C. & Sastry, S. *Hybrid Systems: Modeling, Analysis and Control* (UC Berkeley / ETH Zurich lecture notes, 2008).

[2] Ljung, L. *System identification: theory for the user* (PTR Prentice Hall, Upper Saddle River, NJ 1999).

[3] Ly, D. L. & Lipson, H. Learning symbolic representations of hybrid dynamical systems. *J. Mach. Learn. Res.* **13**, 3585-3618 (2012).

[4] Reger, B. D., Fleming, K. M., Sanguineti, V., Alford, S. & Mussa-Ivaldi, F. A. Connecting brains to robots: an artificial body for studying the computational properties of neural tissues. *Artif. Life* **6**, 307 (2000).

[5] He, Q., Guo, Y., Wang, X., Ren, Z. & Li, J. Gearbox fault diagnosis based on RB-SSD and MCKD. *China Mechanical Engineering* **28**, 1528-1534 (2017).

[6] Hameed, Z., Hong, T. & Cho, Y. Condition monitoring and fault detection of wind turbines and related algorithms. *Renew. Sust. Energ. Rev.* **13**, 1-39 (2009).

[7] Yuan, Y. et al. Artificial intelligent diagnosis and monitoring in manufacturing. Preprint at `https://arxiv.org/abs/1901.02057` (2019).

[8] Fang, X., Misra, S., Xue, G. & Yang, D. Smart grid the new and improved power grid: A survey. *IEEE Commun. Surv. Tutor.* **14**, 944-980 (2012).

[9] Jabr, R. Minimum loss operation of distribution networks with photovoltaic generation. *IET Renew. Power Gener.* **8**, 33-44 (2014).

[10] Baran, M. & Wu, F. Network reconfiguration in distribution systems for loss reduction and load balancing. *IEEE Trans. Power Deliv.* **4**, 1401-1407 (1989).

[11] Zimmerman, R., Murillo-Sanchez, C. & Thomas, R. MATPOWER: Steady-State Operations, Planning, and Analysis Tools for Power Systems Research and Education. *IEEE Trans. Power Syst.* **26**, 12-19 (2011).

[12] Courtemanche, M., Ramirez, R. & Nattel, S. Ionic mechanisms underlying human atrial action potential properties: insights from a mathematical model. *Am. J. Physiol.-Heart Circul. Physiol.* **275**, 301-321 (1998).

[13] Brunton, S. L., Proctor, J. L. & Kutz, J. N. Discovering governing equations from data by sparse identification of nonlinear dynamical systems. *Proc. Natl. Acad. Sci. USA* **113**, 3932-3937 (2016).

[14] Pan, W., Yuan, Y., Goncalves, J. & Stan, G. B. Reconstruction of arbitrary biochemical reaction networks: A compressive sensing approach. In *Proceedings of the 51st IEEE Conference on Decision and Control*, 2334-2339 (2012).

[15] Grant, M. & Boyd, S. CVX: Matlab software for disciplined convex programming, version 2.0 beta. `http://cvxr.com/cvx` (2013).

[16] Yamashita, O., Sato, M. A., Yoshioka, T., Tong, F. & Kamitani, Y. Sparse estimation automatically selects voxels relevant for the decoding of fmri activity patterns. *Neuroimage* **42**, 1414-1429 (2008).

[17] Bako, L. Identification of switched linear systems via sparse optimization. *Automatica* **47**, 668-677 (2011).

[18] Wipf, D. P., Rao, B. D. & Nagarajan, S. Latent variable Bayesian models for promoting sparsity. *IEEE Trans. Inf. Theory* **57**, 6236-6255 (2011).

[19] Wipf, D. P. & Rao, B. D. Sparse Bayesian learning for basis selection. *IEEE Trans. Signal Process.* **52**, 2153-2164 (2004).

[20] Murphy, K.P. *Machine learning: A probabilistic perspective* (MIT Press, 2012).
