# Supplementary material for: Data driven discovery of cyber physical systems
Source: Nat Commun. 2019 Oct 25;10:4894. doi: 10.1038/s41467-019-12490-1 (PMC6814766; doi:10.1038/s41467-019-12490-1)
Supplement: Supplementary file 2 — Description of Additional Supplementary Files [file 41467_2019_12490_MOESM2_ESM.pdf]

Description of Additional Supplementary Files

File Name: Supplementary Movie 1

Description: IHYDE successfully reverse engineered the model of designed autonomous car from data and discovered the software bug
